# Global, Regional, and National Trends in the Burden of Anxiety Disorders From 1992 to 2021: An Age–Period–Cohort Analysis Based on the Global Burden of Disease Study 2021

**DOI:** 10.1155/da/4178541

**Published:** 2025-07-12

**Authors:** Jiali Zhou, Shuting Li, Yuan Song, Jiayao Ying, Zeyu Luo, Shiyi Shan, Liying Zhou, Jindian Zha, Xin Wang, Peige Song, Jianzhong Yang

**Affiliations:** ^1^Department of Psychiatry, The First Affiliated Hospital, Zhejiang University School of Medicine, Hangzhou, Zhejiang, China; ^2^School of Public Health, Zhejiang University School of Medicine, Hangzhou, Zhejiang, China; ^3^School of Nursing and Health, Zhengzhou University, Zhengzhou, Henan, China; ^4^School of Health Management, Anhui Medical University, Hefei, Anhui, China; ^5^Department of Psychiatry, The Second Affiliated Hospital of Kunming Medical University, Kunming, Yunnan, China

**Keywords:** age–period–cohort, anxiety disorders, burden, trend analysis

## Abstract

**Background:** Anxiety disorders pose a considerable global health challenge, ranking as the most prevalent type of mental illness. This study delineates the trends in incidence, prevalence, and years lived with disability (YLDs) for anxiety disorders at global, regional, and national levels between 1992 and 2021, with an emphasis on the independent effects of age, period, and birth cohort.

**Methods:** Incidence, prevalence, and YLDs were selected as burden indicators of anxiety disorders, following the standardized methodologies of the Global Burden of Diseases, Injuries, and Risk Factors Study (GBD) 2021, with data presented as numerical counts and age-standardized rates (ASRs) per 100,000 population. An age-period-cohort (APC) model was applied to estimate the overall annual percentage change (net drift), annual percentage change within each age group (local drift), and the relative risks associated with age, period, and cohort.

**Results:** From 1992 to 2021, the global age-standardized incidence rate (ASIR), age-standardized prevalence rate (ASPR) and age-standardized YLDs rate (ASYR) for anxiety disorders showed an overall increase. The APC model estimated a global net drift of 0.16% (95% confidence interval [CI]: 0.03%, 0.30%) for incidence, 0.07% (95% CI: 0.02%, 0.12%) for prevalence, and 0.07% (95% CI: 0.01%, 0.12%) for YLDs. Regionally, the highest ASIR, ASPR, and ASYR were recorded in the high SDI region in 2021, with the Region of the Americas (AMR) showing the highest rates across all three metrics. Among 204 countries/territories, Portugal, Brazil, Lebanon, Iran, and Paraguay ranked as the top five highest for ASIR, ASPR, and ASYR. The age effects on incidence, prevalence, and YLDs followed similar global and regional patterns, with risk initially increasing before declining in older age. The most substantial increase in the period risk of incidence from 1992 to 2021 occurred in high SDI and AMR countries, while prevalence and YLDs saw the largest rise in low-middle SDI region and AMR. A steady increase in the risk of incidence, prevalence, and YLDs was observed across successive birth cohorts globally and regionally.

**Conclusion:** The global burden of anxiety disorders demonstrated an overall upward trend, with considerable regional, demographic, and temporal variations. These findings provide critical insights for optimizing resource allocation and developing tailored public health strategies to address anxiety disorders.

## 1. Introduction

Anxiety disorders, characterized by excessive fear or worry, include conditions such as panic disorder, generalized anxiety disorder, and specific phobias [1, 2]. Recognized by the World Health Organization (WHO) as the most prevalent mental health condition, these disorders affected 301 million individuals globally in 2019 [1, 2]. The Global Burden of Diseases, Injuries, and Risk Factors Study (GBD) 2021 reported a 16.7% increase in the age-standardized disability-adjusted life years rate for anxiety disorders between 2010 and 2021 [3]. Beyond impairing daily functioning and diminishing quality of life, anxiety disorders impose substantial economic burdens, including increased healthcare costs and lost productivity [4, 5]. In 2019, it accounted for the second-largest proportion of disability-adjusted life years rate among mental disorders, highlighting the substantial health and economic impact [6–8]. Despite the availability of effective prevention and treatment options, many individuals with anxiety disorders lack access to necessary care, presenting challenges worldwide [9, 10]. As mental health gains priority on global health agendas, addressing the burden of anxiety disorders is crucial to guiding public health interventions and informing policy decisions [11, 12].

Previous studies have examined trends in the burden of anxiety disorders; however, many relied on relatively older data [6, 13, 14], analyzed only a single metric of burden (i.e., prevalence or incidence) [15], focused on specific regions [16], targeted particular populations [16, 17] or lacked in-depth insights into the temporal trends [18]. Consequently, there remains limited understanding of the distinct age, period, and cohort effects that shape these trends [14]. Differentiating these effects is pivotal for developing targeted public health strategies. Age effects describe variations in disease rates across different age groups, suggesting that certain age groups may be more susceptible to anxiety disorders. Period effects reflect changes occurring during specific timeframes that impact all age groups and cohorts uniformly, often driven by socioeconomic, environmental, or cultural shifts. Cohort effects capture differences in risk among individuals born within the same time period, independent of aging, which may be attributed to shared environmental exposures or societal changes. Understanding these effects is essential for evaluating the impact of past policy interventions and identifying target populations to reduce the future burden of anxiety disorders.

To fill these knowledge gaps, our study utilized data from the GBD 2021, which incorporates the impact of the Coronavirus Disease 2019 (COVID-19) pandemic—a considerable global event with profound implications for mental health. According to WHO estimates, the COVID-19 pandemic led to a substantial 25.6% increase in cases of anxiety disorders within 1 year [19]. The GBD 2021 provides a robust tool for comprehensively assessing the incidence, prevalence, and years lived with disability (YLDs) due to anxiety disorders. By employing an age–period–cohort (APC) analysis, this study aimed to (1) systematically delineate the trends of the incidence, prevalence, and YLDs for anxiety disorders at global, regional, and national levels across 204 countries and territories for the period 1992–2021; and (2) evaluate the independent effects of age, period, and birth cohort on the burden of anxiety disorders.

## 2. Methods

### 2.1. Data Source

The GBD 2021 study (available at https://vizhub.healthdata.org/gbd-results/) [20] estimated the burden of 371 diseases, injuries, and impairments, as well as 88 risk factors, across different age groups, sexes, and 204 countries and territories [21, 22]. Uncertainty was addressed by propagating 1000 draws through all estimation stages, with 95% uncertainty intervals (UIs) defined as the 2.5th and 97.5th percentiles [20]. Detailed descriptions of data sources and their validation processes are systematically reviewed and accessible via the Global Health Data Exchange (GHDx) web tool (http://ghdx.healthdata.org/) [23]. This study presents the numbers and age-standardized rates (ASRs), along with their 95% UIs for incidence, prevalence, and YLDs related to anxiety disorders from 1992 to 2021, disaggregated by sex, age, region, and country [24].

Additionally, this study employed the sociodemographic index (SDI), a composite measure of fertility rates under 25, education levels for adults over 15, and income per capita [25], scaled from zero (denoting minimal development relevant to health) to one (denoting maximal development), to reflect social and economic conditions for health outcomes. Countries/territories were divided into five SDI quintiles (high SDI [>0.810], high–middle SDI [0.712–0.810], middle SDI [0.619–0.712], low–middle SDI [0.466–0.619], and low SDI [<0.466] [25]) and six WHO regions (the African Region [AFR], the Region of the Americas [AMR], the Eastern Mediterranean Region [EMR], the European Region [EUR], the South-East Asia Region [SEAR], and the Western Pacific Region [WPR]) [26].

### 2.2. Disease Definition

According to the Institute for Health Metrics and Evaluation (IHME), anxiety disorders, as defined by The Diagnostic and Statistical Manual of Mental Disorders, fourth edition, text revision (DSM-IV-TR) and the International Statistical Classification of Diseases and Related Health Problems 10th Revision (ICD-10) diagnostic criteria [27, 28], are characterized by intense fear, distress, and physiological symptoms that result in disability. Specific anxiety disorders are classified under DSM-IV-TR codes 300.0–300.3, 208.3, 309.21, 309.81, and ICD-10 codes F40-42, F43.0, F43.1, F93.0-93.2, and F93.8 [27, 28]. This study evaluated the overall burden of anxiety disorders collectively, rather than analyzing individual subtypes.

### 2.3. Statistical Analysis

#### 2.3.1. Burden Description

Using the GBD world population age standard, direct standardization was applied to individuals aged 1 year and older with anxiety disorders to account for population age structure and allow for consistent comparisons of disease burden across years and locations. The age-standardized incidence rate (ASIR) denotes the number of incident cases per 100,000 population, the age-standardized prevalence rate (ASPR) indicates the number of existing cases per 100,000 population, and the age-standardized YLDs rate (ASYR) represents the YLDs per 100,000 population. This study reported the distribution of ASIR, ASPR, and ASYR by sex across the globe, five SDI regions, six WHO regions, and 204 countries/territories from 1992 to 2021.

#### 2.3.2. APC Analysis

This study employed an APC model to estimate the independent effects of age, period, and birth cohort on the incidence, prevalence, and YLD rates of anxiety disorders [29, 30]. The APC model, a robust improvement over conventional analyses, separates the influences of biological aging, technological advancements, and social factors on disease trends [31]. As a substantial advancement over traditional epidemiological analyses, the APC model disentangles the influences of biological aging, technological progress, and social changes on disease trends. It quantifies the additive effects of these three components and addresses the statistical identification challenge (age = period-birth cohort) by generating estimable parameters without imposing arbitrary constraints [31, 32]. The net drift, expressed as the annual percentage change of ASRs (% per year), reflects the combined trends attributable to calendar time and successive birth cohorts. The local drifts, expressed as annual percentage change of age-specific ASRs (% per year), capture cohort-specific trends by illustrating the log-linear relationship between period and cohort effects within each age group. Besides, the model results demonstrated the longitudinal age curves, the period rate ratio (RR), and the cohort RR. To enhance interpretability, ASRs and population data were aggregated into six consecutive 5-year periods, spanning 1992–1996 (midyear, 1994.5) to 2017–2021 (midyear, 2019.5). The longitudinal age curves depict the fitted longitudinal ASRs for the reference cohort, adjusted for period deviations, across 20 age groups ranging from 0 to 4 years old to 95+ years old in 5-year intervals. The period RR refers to the relative risk in a period relative to the reference period, while the cohort RR represents the relative risk of a birth cohort compared to the reference birth cohort, both adjusted for age and nonlinear period effects. This analysis incorporated 25 consecutive 5-year birth cohorts, from 1897 to 1901 (midyear, 1899) to 2017–2021 (midyear, 2019). The central calendar period (2004.5, 2002–2006) served as the reference for calculating the period RR, while the central birth cohort (1959, 1957–1961) was used as the reference for the cohort RR [32]. Furthermore, we disaggregated these effects by sex, SDI regions, and WHO regions.

All analyses and visualizations were conducted in R version 4.3.2 (https://www.r-project.org). A two-sided *p* of less than 0.05, or a 95% UI or confidence interval (CI) that did not cross 0.00 were considered statistically significant.

## 3. Results

### 3.1. Trends in Burden of Anxiety Disorders From 1992 to 2021

Globally, the total cases of incidence, prevalence, and YLDs for anxiety disorders increased from 1992 to 2021. Simultaneously, the ASIR of anxiety disorders rose slightly from 559.73 per 100,000 population (95% UI: 465.56, 682.02) in 1992 to 563.61 per 100,000 population (95% UI: 468.74, 687.84) in 2019, followed by a marked rise to 678.25 per 100,000 population (95% UI: 565.15, 832.44) in 2021. The ASPR declined marginally from 3730.30 per 100,000 population (95% UI: 3225.25, 4339.52) in 1992 to 3709.41 per 100,000 population (95% UI: 3190.77, 4335.18) in 2019, followed by a notable increase to 4421.87 per 100,000 population (95% UI: 3768.28, 5182.08) in 2021. The ASYR also decreased slightly from 442.05 per 100,000 population (95% UI: 305.31, 601.07) in 1992 to 440.22 per 100,000 population (95% UI: 304.57, 600.89) in 2019, before rising substantially to 524.33 per 100,000 population (95% UI: 363.06, 716.25) in 2021. In terms of sex disparities, females exhibited higher numbers and ASRs compared to their male counterparts. From 1992 to 2021, the APC model estimated a global net drift in anxiety disorders, with annual increases of 0.16% (95% CI: 0.03%, 0.30%) for incidence, 0.07% (95% CI: 0.02%, 0.12%) for prevalence, and 0.07% (95% CI: 0.01%, 0.12%) for YLDs (Table 1). The annual percentage change in incidence, prevalence, and YLDs for each age group, as determined by the local drift from the APC model, is presented in Table S1. The incidence of anxiety disorders exhibited an increasing trend across nearly all age groups, while the prevalence demonstrated an upward trend from the 0–4 to 40–44 age groups, followed by a decline. YLDs also trended upward for most age groups until 65 years, followed by a decrease thereafter.

In 2021, across the five SDI regions, the high SDI region had the highest ASIR (841.56 [95% UI: 702.07, 1030.93] per 100,000 population), ASPR (5639.51 [95% UI: 4807.41, 6672.65] per 100,000 population), and ASYR (668.99 [95% UI: 463.37, 917.34] per 100,000 population). From 1992 to 2021, the low–middle SDI region showed the most notable increases, with annual increases of 0.26% (95% CI: 0.03%, 0.49%) for incidence, 0.33% (95% CI: 0.20%, 0.45%) for prevalence, and 0.34% (95% CI: 0.21%, 0.48%) for YLDs (Table 1). In 2021, among the six WHO regions, the ASIR, ASPR, and ASYR were all highest in the AMR (ASIR: 957.80 [95% UI: 782.34, 1193.78] per 100,000 population; ASPR: 6699.11 [95% UI: 5739.46, 7910.15] per 100,000 population; and ASYR: 790.11 [95% UI: 543.79, 1074.42] per 100,000 population). From 1992 to 2021, the most notable increases were observed in the EUR, with annual increases of 0.31% (95% CI: 0.22%, 0.40%) for incidence, 0.29% (95% CI: 0.26%, 0.33%) for prevalence, and 0.30% (95% CI: 0.27%, 0.34%) for YLDs (Table 1).

**Figure 1:**
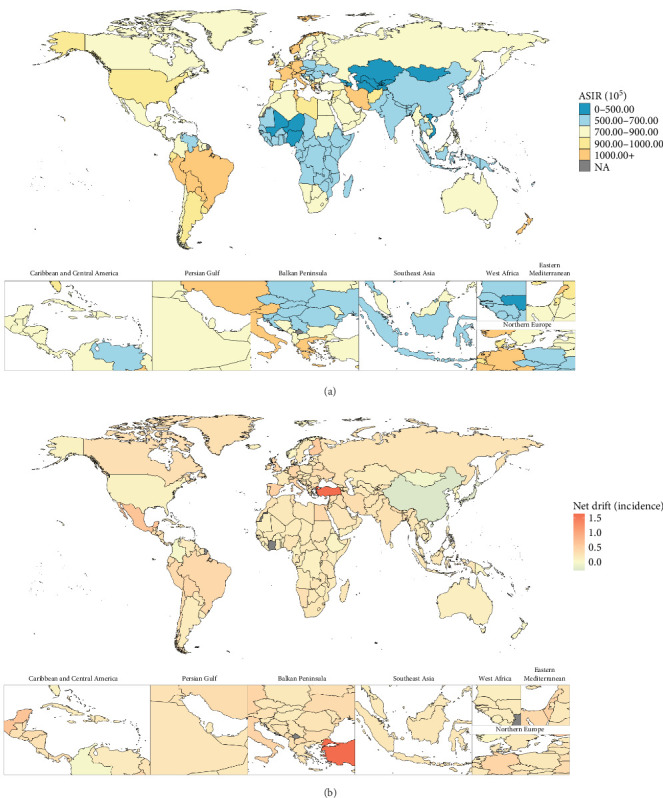
Global distribution for age-standardized incidence rate of anxiety disorders in 2021 as well as its net drift from 1992 to 2021. Note: (A) Global map for age-standardized incidence in 2021. (B) Global map for net drift of incidence from 1992 to 2021. Source: Institute for Health Metrics and Evaluation. Used with permission. All rights reserved.

**Figure 2:**
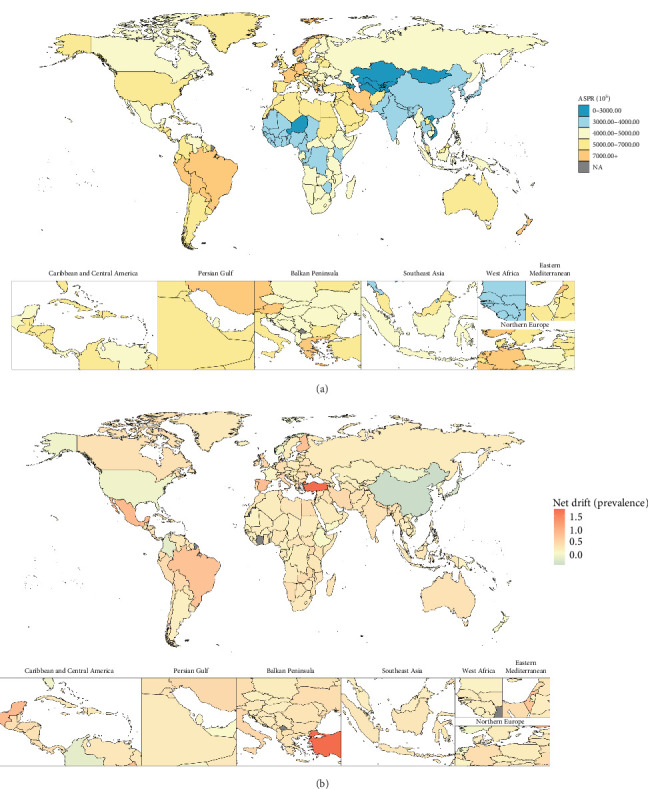
Global distribution for age-standardized prevalence rate of anxiety disorders in 2021 as well as its net drift from 1992 to 2021. Note: (A) Global map for age-standardized prevalence in 2021. (B) Global map for net drift of prevalence from 1992 to 2021. Source: Institute for Health Metrics and Evaluation. Used with permission. All rights reserved.

**Figure 3:**
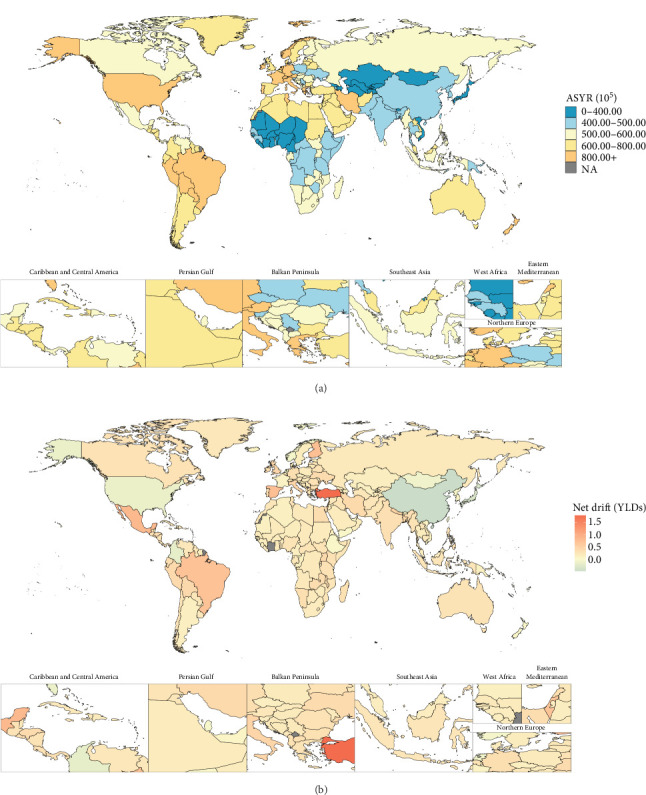
Global distribution for age-standardized years lived with disability rate of anxiety disorders in 2021 as well as its net drift from 1992 to 2021. Note: (A) Global map for age-standardized YLDs in 2021. (B) Global map for net drift of YLDs from 1992 to 2021. YLDs, years lived with disability. Source: Institute for Health Metrics and Evaluation. Used with permission. All rights reserved.

In 2021, the 204 countries/territories with the highest ASIR were Portugal (1290.64 [95% UI: 929.74, 1775.32] per 100,000 population), Brazil (1209.77 [95% UI: 993.15, 1503.98] per 100,000 population), Lebanon (1168.05 [95% UI: 824.02, 1570.70] per 100,000 population), Iran (1142.02 [95% UI: 938.58, 1401.35] per 100,000 population), and Paraguay (1123.41 [95% UI: 804.57, 1552.53] per 100,000 population) (Figure 1). The top five for ASPR and ASYR per 100,000 population, ranked from highest to lowest, were Portugal (ASPR: 9712.44 [95% UI: 6864.89, 13062.42]; ASYR: 1156.75 [95% UI: 692.93, 1723.82]), Brazil (ASPR: 9007.36 [95% UI: 7692.54, 10470.78]; ASYR: 1058.67 [95% UI: 732.51, 1442.26]), Paraguay (ASPR: 8390.01 [95% UI: 6086.82, 11223.56]; ASYR: 992.12 [95% UI: 637.27, 1514.56]), Lebanon (ASPR: 8274.66 [95% UI: 5766.56, 11065.29]; ASYR: 981.03 [95% UI: 592.83, 1442.83]), and Iran (ASPR: 8198.38 [95% UI: 7057.54, 9426.24]; ASYR: 973.70 [95% UI: 659.07, 1345.76]) (Figures 2 and 3). Between 1992 and 2021, net drifts estimated from the APC model indicated increasing trends in the incidence, prevalence, and YLDs of anxiety disorders, with over 190 of the 204 countries and territories showing rises across all three metrics (Figures 1–3). For detailed country-specific data, please refer to Table S2, Figures S1–S3.

### 3.2. Age, Period, and Birth Cohort Effects on the Burden of Anxiety Disorders

**Figure 4:**
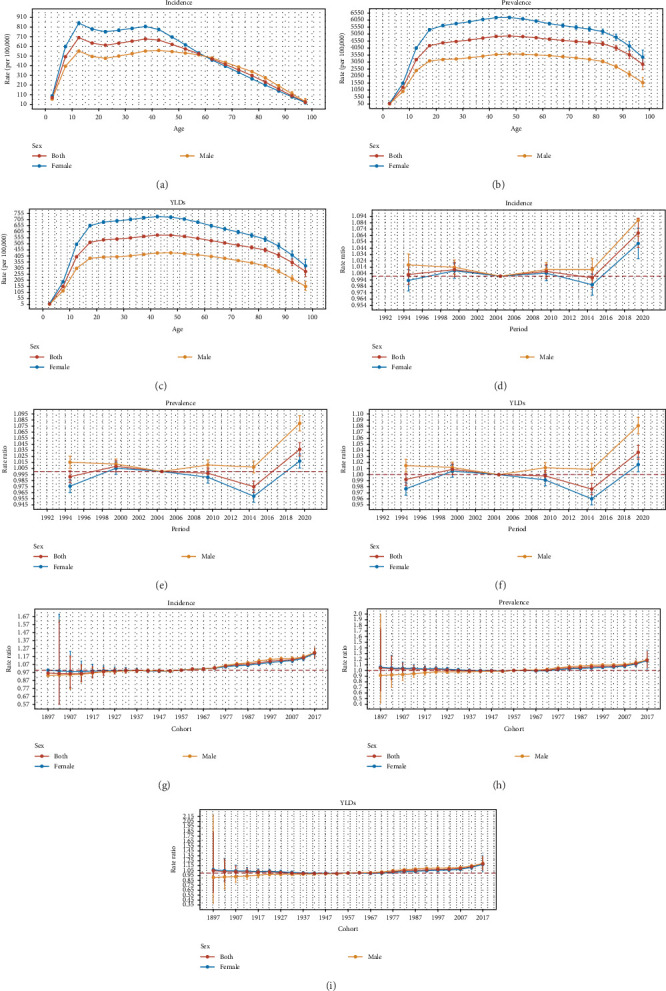
Age, period and cohort effects on incidence, prevalence and years lived with disability of anxiety disorders by sex from 1992 to 2021. (A) Age effects are shown by the fitted longitudinal age curves of incidence rates (per 100,000 person-years) adjusted for period deviations. (B) Age effects are shown by the fitted longitudinal age curves of prevalence rates (per 100,000 person-years) adjusted for period deviations. (C) Age effects are shown by the fitted longitudinal age curves of YLDs (per 100,000 person-years) adjusted for period deviations. (D) Period effects are shown by the relative risk of incidence and computed as the ratio of age-specific rates from 1992–1996 to 2017–2021, with the reference period set at 2002–2006. (E) Period effects are shown by the relative risk of prevalence and computed as the ratio of age-specific rates from 1992–1996 to 2017–2021, with the reference period set at 2002–2006. (F) Period effects are shown by the relative risk of YLDs and computed as the ratio of age-specific rates from 1992–1996 to 2017–2021, with the reference period set at 2002–2006. (G) Cohort effects are shown by the relative risk of incidence and computed as the ratio of age specific rates from the 1897 birth cohort to the 2017 cohort, with the reference cohort set at 1957. (H) Cohort effects are shown by the relative risk of prevalence and computed as the ratio of age specific rates from the 1897 birth cohort to the 2017 cohort, with the reference cohort set at 1957. (I) Cohort effects are shown by the relative risk of YLDs and computed as the ratio of age specific rates from the 1897 birth cohort to the 2017 cohort, with the reference cohort set at 1957. The data points and error bars denote incidence, prevalence, and YLDs or rate ratios and their corresponding 95% CIs. CI, confidence interval; YLDs, years lived with disability. Source: Institute for Health Metrics and Evaluation. Used with permission. All rights reserved.

The age, period, and birth cohort effects derived from the APC model are illustrated in Figure 4, Tables S3–S11, Figures S4–S9. Globally, and across most SDI regions and WHO regions, the age effects on incidence demonstrated a sharp increase during childhood, peaking in adolescence or early adulthood, followed by a steady decline. Females consistently exhibited higher rates than males before 60–70 years. However, this pattern differed in low–middle and low SDI regions, AMR, and SEAR. In terms of prevalence and YLDs, global and regional age effects revealed a gradual increase beginning in childhood, peaking in individuals aged 40–49, and subsequently declining. Notably, in high–middle and high SDI regions, as well as in AFR, EMR, EUR, and WPR, the peak occurred earlier. This pattern was consistent across both sexes, with females exhibiting higher prevalence rates than males at all ages (Figure 4A–C, Tables S3, S6, and S9, Figures S4–S9).

Overall, period effects revealed an initial decline followed by an increase in the risk of incidence across different SDI regions and WHO regions, with the exception of the high SDI region, AMR, and WPR. High SDI region and AMR consistently exhibited lower period risks throughout the study period, while other regions generally experienced higher risks. The most pronounced increase in period risk was observed in high SDI region and AMR between 1992 and 2021. This increase was more significant among males than females, except in the high SDI region and EMR. In terms of prevalence and YLDs, period effects demonstrated an overall upward trend at global and regional levels, except for WPR. The most notable rise in period risk occurred in the low–middle SDI region and AMR between 1992 and 2021. This increase was more considerable among males than females, except in high SDI region and EMR (Figure 4D–F, Tables S4, S7, and S10, Figures S4–S9).

Regarding birth cohort effects, there was a gradual increase in the risk of incidence, prevalence, and YLDs across SDI regions and most WHO regions, with each successive cohort experiencing progressively worse outcomes, except in the WPR. This effect was particularly pronounced in the EUR, where the rise in incidence, prevalence, and YLDs was more marked (Figure 4G–I, Tables S5, S8 and S11, Figure S4–S9).

## 4. Discussion

In 2021, the WHO proposed the Comprehensive Mental Health Action Plan 2013–2030, outlining a framework to promote mental well-being, prevent mental disorders, and enhance access to care [33]. As the most prevalent mental illness globally, anxiety disorders represent a substantial public health challenge and are directly aligned with this initiative. A thorough understanding of the anxiety disorders burden is essential for guiding effective interventions and informing policy decisions. This study provides the most detailed analysis to date of global, regional, and national estimates of anxiety disorder incidence, prevalence, and YLDs from 1992 to 2021. By incorporating APC models, it examines global temporal trends and enables regional comparisons. Our analysis revealed increasing absolute cases of anxiety disorders spanning three decades, alongside an overall increase in ASRs. Compared with the prior GBD 2019 publication, this analysis leverages updated data to generate deeper insights into disease trends, yielding meaningful public health implications. By disentangling age, period, and cohort effects across SDI and WHO regions, this study offers critical insights into the trends of incidence, prevalence, and YLD rates, which highlights the effectiveness of anxiety disorder-related health care services.

Globally, the incidence of anxiety disorders was estimated at 53.92 million cases in 2021, with a total of 359.21 million people affected. Consistent with previous studies [3, 14], our analysis showed an overall increase in the incidence, prevalence, and YLDs for anxiety disorders over the past three decades, reflecting constant growth in disease burden. This growth may be attributed to population expansion [6, 14], heightened health awareness [34], and improved diagnostic practices [35, 36], resulting in higher reporting rates. Additionally, our findings aligned with previous research highlighting sex disparities in the burden of anxiety disorders [6, 14]. Females consistently exhibited higher incidence, prevalence, and YLDs than their male counterparts, with the 2021 ASYR for females reaching 652.17 per 100,000 population compared to 396.91 per 100,000 population for males. This pattern suggested that biological factors, such as estrogen, along with societal roles and greater susceptibility, may considerably influence the burden of anxiety disorders [37–39]. These rising trends and sex patterns underscore the urgent need for global health systems to adapt to the evolving mental health landscape.

Our regional and national analysis from 1992 to 2021 demonstrated significant disparities in the burden of anxiety disorders, reflecting the varied impacts of healthcare systems, socioeconomic conditions, and cultural influences. High SDI region and the AMR recorded the highest ASRs, likely due to heightened social and economic pressures in more advanced societies. Additionally, improved diagnostic capabilities, better access to mental health services, and greater awareness of seeking medical care have facilitated the detection and reporting of anxiety disorders [14, 34, 35, 40]. Over the past three decades, the most notable increases in anxiety disorder burden were observed in the low–middle SDI region, primarily driven by rapid urbanization, economic transitions, and social changes [41, 42]. Similarly, the EUR experienced significant increases, attributed to prolonged economic uncertainty, and widening social inequality [43, 44]. Recent global crises, including the COVID-19 pandemic, migration, and economic challenges, have further exacerbated the mental health burden, with work-related stress and rising living costs contributing to the growing prevalence of anxiety disorders in Europe [43, 45]. In 2021, Portugal reached the highest level for ASIR, ASPR, and ASYR, as a highly developed country, which may be related to its high level of economic development, social pressures, cultural environment, aging population, as well as improved diagnostic capabilities and greater public awareness [46]. This complex pattern highlights the need for region-specific strategies that account for local cultural, economic, and social contexts to mitigate the global mental health burden and improve outcomes.

The analysis of local drift and age effects revealed the temporal trends in the incidence, prevalence, and YLDs of anxiety disorders across different age groups. Consistent with previous studies [14, 37, 47], the incidence of anxiety disorders increased across nearly all age groups. The prevalence and YLDs showed an upward trend from young age to middle age, then declined. These trends likely reflect the early onset, chronic nature, and frequent comorbidity of anxiety disorders with somatic conditions. Anxiety disorders often emerge in childhood or adolescence, peaking in middle age, as younger and middle-aged individuals face increased exposure to stressors such as career demands, financial pressures, family responsibilities, and economic uncertainty, all of which heighten the risk of anxiety disorders [43, 44, 48]. In older adults, despite a rising incidence, anxiety disorders may be underdiagnosed due to cognitive decline or the overshadowing presence of other chronic health conditions, potentially explaining the observed decrease in prevalence and YLDs. Additionally, as people age—particularly after middle age—reduced work obligations, improved coping strategies, and more effective stress management through activities like community engagement contribute to lower anxiety levels [48, 49]. Thus, early identification of high-risk individuals and timely interventions—particularly psychological support for younger and middle-aged adults—are critical for effective management [18, 50].

Our findings also indicated the presence of unfavorable period and cohort effects in anxiety disorders. From 1992 to 2021, the greatest increase in incidence was seen in high-SDI region and AMR countries, while low–middle SDI region and AMR experienced the most pronounced rise in prevalence and YLDs, underscoring regional disparities in the burden of anxiety disorders. In recent years, period-related risks have continued to escalate, likely driven by the cumulative impact of factors such as the enduring effects of the COVID-19 pandemic, widespread unemployment, accelerated work pace, and heightened competition [6, 43, 48, 51]. Regarding birth cohort effects, individuals born in more recent years faced a higher overall risk of anxiety disorders than those born earlier, particularly after 2019, potentially due to increased societal pressures, including rising competition, economic instability, and a rapidly evolving job market [6, 43, 48, 51]. Furthermore, while strong social connections typically serve as a buffer against stress, contemporary younger populations appear to struggle more with forming and maintaining these connections, further exacerbating their vulnerability to anxiety disorders [52–54]. These trends underscore a marked deterioration and the urgent need for enhanced disease control strategies.

This study has several prominent merits. To the best of our knowledge, it represents the most comprehensive and up-to-date investigation of the incidence, prevalence, and YLDs for anxiety disorders over the past three decades, which advances our understanding of its trends at global, regional, and national levels. The application of the APC model, a robust analytical framework, allows for the disentangling of age-related biological factors, period-specific socioeconomic influences, and cohort-specific environmental exposures driving disease trends. This multidimensional approach offers a deeper understanding of the underlying drivers of anxiety disorders, surpassing the limitations of traditional epidemiological metrics, and enhancing insights into health system responses. Lastly, the use of GBD 2021, the most extensive and current global health database, provides critical evidence to guide public health policy and resource allocation worldwide. Despite these strengths, several limitations must be acknowledged. First, reliance on GBD data introduces potential inconsistencies due to variability in data collection methods and periodic updates. Since the GBD database primarily relies on national and regional reports rather than direct submissions, gaps in data completeness, quality, and timeliness may affect accuracy and comparability. Second, variations in disease management practices, including diagnosis, reporting, and recording, across countries and regions may further compromise data reliability. Last, this analysis does not include estimates for specific anxiety disorder subtypes or assessments of symptom severity, as the GBD database lacks the necessary granularity for such evaluations.

The findings of our study provide valuable insights into the epidemiology of anxiety disorders and carry important policy implications. This study offers the most up-to-date and comprehensive estimates of the incidence, prevalence, and YLDs for anxiety disorders at global, regional, and national levels over the past three decades. By incorporating APC models, it systematically disentangles the independent effects of age, period, and cohort, providing a deeper understanding of the temporal trends and distinct drivers of the burden of anxiety disorders. For instance, age effects identify the most vulnerable groups requiring targeted interventions, period effects capture the influence of socioeconomic or cultural changes, and cohort effects reveal risks linked to shared environmental exposures. In addition, by comparing pre-COVID-19 and COVID-19 periods, this study highlights a substantial increase in the global and regional burden of anxiety disorders following the onset of the pandemic, which underscores the urgent need for region-specific and demographically tailored mental health strategies to address the heightened burden in the postpandemic era. Furthermore, this study highlights substantial geographic and socioeconomic variability in the burden of anxiety disorders, which provides evidence to guide resource allocation and inform the development of tailored prevention and management strategies. Despite the availability of effective prevention and treatment options, significant gaps remain in the treatment, monitoring, and intervention of anxiety disorders worldwide, presenting challenges in both low-income and high-income countries. As mental health rises on the global health agenda, understanding the burden of anxiety disorders is essential for shaping effective public health responses. These findings offer actionable evidence to inform policy decisions and resource allocation aimed at reducing the burden of anxiety disorders at global, regional, and national levels.

## 5. Conclusions

This study leveraged data from GBD 2021 to comprehensively delineate the burden of anxiety disorders at global, regional, and national levels, assessing trends over a period from 1992 to 2021. As a major public health issue, the global burden of anxiety disorders demonstrated an overall upward trend during this period. Furthermore, this study identified disparities in anxiety disorder burden across different regions and countries, as well as variations by age, sex, period, and cohort. These results collectively underscored the substantial challenges in the control and management of anxiety disorders, which may be instructive for better formulating public health policy and allocating medical resources. A coordinated effort by governments and the global health community is urgently warranted to address the current and future gaps in the prevention and treatment of anxiety disorders.

## Figures and Tables

**Table 1:** The number and age-standardized rate of incidence, prevalence, year live with life disability of anxiety disorders, and its trends from 1992 to 2021.

	Incidence							Prevalence							YLDs						
	1992		2019		2021		1992–2021	1992		2019		2021		1992–2021	1992		2019		2021		1992–2021
	Number	ASR	Number	ASR	Number	ASR	Net drift	Number	ASR	Number	ASR	Number	ASR	Net drift	Number	ASR	Number	ASR	Number	ASR	Net drift
	No. (95% UI)	No. (95% UI)	No. (95% UI)	No. (95% UI)	No. (95% UI)	No. (95% UI)	(% per year)	No. (95% UI)	No. (95% UI)	No. (95% UI)	No. (95% UI)	No. (95% UI)	No. (95% UI)	(% per year)	No. (95% UI)	No. (95% UI)	No. (95% UI)	No. (95% UI)	No. (95% UI)	No. (95% UI)	(% per year)
Global																					
	31,086,760 (25,760,376, 38,402,133)	559.73 (465.56, 682.02)	44,048,160 (36,627,315, 53,668,008)	563.61 (468.74, 687.84)	53,917,371 (44,990,958, 65,978,475)	678.25 (565.15, 832.44)	0.16 (0.03, 0.30)	198,887,797 (170,074,950, 23,3354,849)	3730.30 (3225.25, 4339.52)	295,114,826 (254,462,839, 344,488,878)	3709.41 (3190.77, 4335.18)	359,213,270 (307,185,425, 419,966,577)	4421.87 (3768.28, 5182.08)	0.07 (0.02, 0.12)	23,709,063 (16,342,920, 32,524,132)	442.05 (305.31, 601.07)	34,973,486 (24,213,274, 47,623,948)	440.22 (304.57, 600.89)	42,509,645 (29,396,723, 57,729,820)	524.33 (363.06, 716.25)	0.07 (0.01, 0.12)
Sex																					
Male	13,245,499 (10,995,890, 16095405)	478.31 (400.99, 577.90)	19,146,884 (16,081,771, 23,068,106)	485.12 (407.42, 586.63)	23,187,398 (19,477,816, 28,113,826)	576.46 (486.27, 701.84)	0.19 (0.04, 0.34)	75,053,691 (64,099,776, 88,335,844)	2798.32 (2408.94, 3266.60)	111,652,939 (95,503,347, 130,323,344)	2808.58 (2408.5, 3289.58)	134,403,655 (114,699,149, 157,908,850)	3308.52 (2830.39, 3887.81)	0.17 (0.11, 0.24)	9,042,577 (6,203,914, 12,539,968)	334.87 (231.07, 459.13)	13,392,072 (9,173,200, 18,432,352)	336.88 (231.16, 464.46)	16,113,657 (11,067,291, 22,164,952)	396.91 (272.28, 547.66)	0.18 (0.11, 0.25)
Female	17,841,261 (14,679,616, 22,037,215)	644.82 (535.09, 789.32)	24,901,276 (20,649,635, 30,431,243)	645.64 (536.94, 791.83)	30,729,973 (25,426,580, 37,775,899)	784.53 (649.26, 966.02)	0.13 (−0.01, 0.27)	123,834,106 (106,034,785, 145,287,882)	4652.83 (4005.32, 5416.94)	183,461,886 (157,946,318, 214,076,056)	4607.57 (3952.3, 5395.59)	224,809,615 (192,230,867, 263,052,153)	5535.61 (4730.42, 6495.30)	0.03 (−0.02, 0.09)	14,666,486 (10,179,688, 19,963,668)	548.51 (381.47, 742.60)	21,581,414 (15,055,457, 29,270,660)	543.57 (379.37, 737.02)	26,395,988 (18,390,434, 35,710,763)	652.17 (453.44, 887.15)	0.03 (−0.03, 0.09)
SDI region																					
Low SDI region	2,584,481 (2,117,793, 3,217,548)	517.30 (423.35, 644.36)	5,529,957 (4,528,075, 6,874,455)	523.99 (430.26, 651.6)	6,788,499 (5,512,876, 8,440,444)	603.86 (496.22, 752.22)	0.18 (−0.02, 0.39)	14,571,963 (12,047,299, 17,800,179)	3279.90 (2753.69, 3935.69)	31,589,908 (26,014,143, 38,658,058)	3321.74 (2783.88, 3968.33)	38,513,782 (31,398,707, 47,545,721)	3782.31 (3110.34, 4583.98)	0.19 (0.11, 0.26)	1,728,868 (1,167,627, 2,402,952)	384.19 (262.26, 523.13)	3,781,203 (2,560,749, 5,281,499)	392.48 (267.68, 535.31)	4,603,364 (3,027,207, 6,403,847)	446.36 (296.14, 609.76)	0.22 (0.14, 0.30)
Low–middle SDI region	6,158,086 (5,094,075, 7,623,457)	526.33 (434.17, 643.25)	10,257,515 (8,473,777, 12,737,660)	536.95 (446.02, 657.28)	12,828,953 (10,543,452, 15,898,000)	649.40 (534.77, 801.33)	0.26 (0.03, 0.49)	35,851,099 (30,210,865, 42,649,472)	3355.84 (2855.82, 3935.65)	63,642,464 (54,121,757, 75,877,628)	3459.07 (2967.45, 4040.33)	78,736,534 (66,913,314, 93,335,234)	4110.28 (3502.94, 4813.65)	0.33 (0.20, 0.45)	4,261,038 (2,922,065, 5,900,151)	394.18 (272.50, 534.93)	7,562,052 (5,209,738, 10,339,964)	408.29 (284.17, 555.19)	9,340,395 (6,459,975, 12,701,317)	484.76 (337.96, 655.59)	0.34 (0.21, 0.48)
Middle SDI region	10,318,351 (8,498,958, 12,664,373)	567.72 (473.17, 686.46)	14,234,140 (11,891,133, 17,173,775)	579.21 (484.45, 698.9)	17,436,716 (14,457,652, 21,191,691)	700.88 (582.42, 859.40)	0.16 (−0.06, 0.37)	63,969,933 (54,790,312, 74,649,521)	3712.78 (3223.09, 4285.30)	95,413,825 (82,433,723, 109,923,970)	3779.92 (3273.44, 4360.01)	117,094,164 (100,351,714, 136,044,666)	4556.61 (3889.42, 5294.11)	0.14 (0.05, 0.23)	7,681,100 (5,306,897, 10,575,772)	441.52 (304.94, 600.50)	11,339,688 (7,765,403, 15,437,398)	450 (309.61, 615.59)	13,889,164 (9,600,906, 18,924,071)	541.94 (374.72, 741.61)	0.14 (0.05, 0.23)
Middle–high SDI region	6,138,479 (5,091,073, 7,497,320)	555.03 (464.33, 672.85)	7,015,421 (5,884,282, 8,437,104)	553.9 (465, 668.02)	8,423,075 (6,984,883, 10,298,263)	669.84 (553.81, 827.68)	0.11 (−0.08, 0.30)	41,102,436 (35,657,329, 47,795,288)	3673.72 (3183.40, 4246.32)	49,545,075 (42,925,029, 57,209,661)	3579.01 (3089.06, 4158.68)	59,670,393 (50,848,624, 69,798,383)	4325.31 (3667.44, 5079.84)	0.02 (−0.05, 0.09)	4,907,145 (3,391,512, 6,689,840)	438.00 (304.72, 599.36)	5,864,105 (4,045,917, 7,994,666)	428.3 (297.83, 587.97)	7,053,070 (4,756,266, 9,536,400)	517.28 (350.45, 711.25)	0.03 (−0.05, 0.11)
High SDI region	5,857,860 (4,863,158, 7,142,708)	658.31 (547.99, 803.18)	6,974,556 (5,806,158, 8,449,562)	685.85 (570.82, 845.96)	8,394,781 (6,982,548, 10,139,111)	841.56 (702.07, 1030.93)	0.15 (0.03, 0.27)	43,198,692 (37,455,291, 50,150,242)	4548.81 (3935.26, 5264.90)	54,671,491 (47,097,603, 63,438,959)	4682.56 (4016.58, 5521.13)	64,890,613 (55,471,021, 75,492,082)	5639.51 (4807.41, 6672.65)	0.02 (−0.04, 0.07)	5,107,893 (3,532,942, 6,924,071)	540.66 (373.12, 739.68)	6,396,589 (4,410,854, 8,631,221)	555.77 (383.16, 761.01)	7,587,224 (5,295,258, 10,197,786)	668.99 (463.37, 917.34)	0.00 (−0.05, 0.06)
WHO region																					
African Region	2,778,120 (2,269,785, 3,455,445)	528.82 (433.34, 662.52)	5,816,466 (4,752,992, 7,242,883)	527.2 (432.42, 660.98)	7,062,991 (5,786,800, 8,762,663)	601.06 (491.75, 746.53)	0.16 (−0.07, 0.39)	15,847,973 (13,047,547, 19,311,885)	3391.95 (2840.25, 4056.24)	33,661,312 (27,815,162, 41,126,667)	3388.16 (2836.06, 4064.3)	40,643,979 (33,495,353, 50,077,443)	3826.01 (3187.85, 4643.71)	0.17 (0.09, 0.24)	1,889,525 (1,275,289, 2,644,495)	399.34 (271.39, 543.32)	4,036,905 (2,722,442, 5,635,784)	401.21 (271.84, 547.35)	4,867,903 (3,229,884, 6,829,176)	452.51 (304.24, 625.12)	0.19 (0.11, 0.27)
Eastern Mediterranean Region	2,704,212 (2,203,447, 3,351,702)	675.47 (560.90, 840.13)	5,031,268 (4,154,769, 6,318,282)	672.43 (557.61, 841.94)	6,193,542 (5,018,938, 7,742,616)	790.60 (646.81, 980.27)	0.24 (0.00, 0.48)	16,466,105 (13,652,620, 19,910,796)	4576.33 (3843.23, 5452.56)	32,348,571 (26,857,579, 39,460,877)	4548.07 (3823.74, 5424.99)	39,542,191 (32,376,444, 47,799,366)	5281.09 (4380.53, 6353.26)	0.28 (0.21, 0.36)	1,974,639 (1,357,032, 2,775,287)	542.15 (373.44, 743.32)	3,862,749 (2,632,405, 5,338,662)	538.62 (369.43, 733.14)	4,712,892 (3,186,188, 6,644,660)	624.75 (426.07, 877.47)	0.28 (0.19, 0.36)
European Region	5,302,696 (4,427,797, 6,484,456)	618.06 (516.42, 755.01)	5,590,480 (4,665,883, 6,783,242)	633.42 (526.05, 785.72)	6,968,616 (5,782,923, 8,478,047)	807.58 (668.26, 997.22)	0.31 (0.22, 0.40)	38,873,374 (33,636,135, 44,870,050)	4283.78 (3720.63, 4968.46)	43,324,092 (36,911,496, 50,588,282)	4348.94 (3696.59, 5163.16)	52,647,634 (44,715,657, 61,530,879)	5401.27 (4536.69, 6407.88)	0.29 (0.26, 0.33)	4,591,316 (3,220,665, 6,194,268)	508.67 (353.30, 694.54)	5,084,157 (3,525,814, 6,830,614)	517.72 (355.4, 708.14)	6,180,620 (4,229,867, 8,374,587)	643.24 (442.26, 881.17)	0.30 (0.27, 0.34)
Region of the Americas	5,321,778 (4,400,065, 6,551,218)	710.10 (589.13, 868.46)	7,698,665 (6,335,708, 9,452,711)	756.19 (623.42, 931.35)	9,756,279 (7,993,529, 12,126,469)	957.80 (782.34, 1193.78)	0.24 (0.04, 0.45)	36,301,168 (31,417,537, 42,294,738)	4975.98 (4295.38, 5749.14)	58,290,186 (50,040,497, 67,586,746)	5424.72 (4666.69, 6309.27)	72,497,660 (61,891,423, 85,021,973)	6699.11 (5739.46, 7910.15)	0.22 (0.13, 0.31)	4,302,206 (2,992,403, 5,836,399)	587.86 (407.48, 795.52)	6,847,317 (4,740,197, 9,246,518)	640.29 (447, 868.52)	8,504,810 (5,856,291, 11,504,539)	790.11 (543.79, 1074.42)	0.21 (0.11, 0.30)
South-East Asia Region	6,414,126 (5,283,003, 7,918,286)	487.69 (404.61, 592.72)	10,246,671 (8,518,028, 12,437,508)	485.81 (406.44, 587.43)	12,990,879 (10,747,249, 15,932,223)	600.75 (500.32, 732.74)	0.25 (−0.23, 0.72)	37,144,896 (31,613,462, 43,870,476)	3060.55 (2626.13, 3562.56)	63,409,684 (54,488,527, 73,535,998)	3051.05 (2627.71, 3522.66)	79,458,002 (67,139,634, 92,828,424)	3694.33 (3144.16, 4306.21)	0.25 (0.04, 0.46)	4,405,878 (3,024,484, 6,041,536)	358.79 (246.70, 486.67)	7,508,736 (5,174,268, 10,216,067)	359.75 (246.39,488.66)	9,394,825 (6,538,747, 12,689,870)	435.43 (302.20, 587.84)	0.28 (0.05, 0.50)
Western Pacific Region	8,385,699 (6,911,315, 10,251,792)	520.03 (436.45, 624.84)	9,478,162 (8,050,325, 11,261,418)	504.11 (428.16, 598.12)	10,740,036 (8,967,049, 12,835,343)	566.45 (472.32, 689.88)	−0.15 (−0.54, 0.24)	53,079,232 (45,569,008, 61,887,552)	3316.70 (2864.81, 3830.39)	62,724,424 (54,802,242, 71,589,995)	3108.83 (2682.97, 3581.5)	72,932,438 (62,746,422, 84,902,456)	3568.44 (3039.14, 4146.63)	−0.31 (−0.45,−0.17)	6,403,905 (4,441,628, 8,812,758)	397.97 (276.85, 544.51)	7,473,312 (5,178,413, 10,175,896)	374.16 (261.67, 512.11)	8,672,686 (6,000,994, 11,795,917)	429.03 (297.18, 586.77)	−0.31 (−0.46,−0.15)

*Note:* Age-standardized rates are presented per 100,000. *Source*: Institute for Health Metrics and Evaluation. Used with permission. All rights reserved.

Abbreviations: ASR, age-standardized rate; SDI, sociodemographic index; UI, uncertainty interval; WHO, World Health Organization; YLDs, years lived with disability.

## Data Availability

The data used in this study were derived from the GBD 2021 database, which is publicly accessible at https://vizhub.healthdata.org/gbd-results/. As the GBD estimates are updated regularly and may vary substantially between versions due to methodological improvements and newly available data, we have not deposited our dataset and analysis code in an open repository to prevent potential inconsistencies with future GBD releases. Researchers interested in accessing the data and code used in this study are encouraged to contact the corresponding author.

## Supporting Information

Supporting Information Table S1. Annual percentage change for the incidence, prevalence, and years lived with disability of anxiety disorders in each age group stratified by sex from 1992 to 2021 (local drift with 95% CI). Table S2. The number and age-standardized rate (per 100,000) of incidence, prevalence, and years lived with disability of anxiety disorders in 204 countries and territories in 1992 and 2021, and its temporal trends from 1992 to 2021. Table S3. Fitted longitudinal age effects of the incidence, prevalence, and years lived with disability of anxiety disorders (per 100,000 person-years) and the corresponding 95% CIs stratified by sex. Table S4. Relative risks for the incidence, prevalence, and years lived with disability of anxiety disorders for each period compared with the reference period (2002–2006) and the corresponding 95% CIs stratified by sex. Table S5. Relative risk for anxiety disorders incidence, prevalence, and years lived with disability of each birth cohort compared with the reference (cohort 1957−1961) and the corresponding 95% CIs by sex. Table S6. Fitted longitudinal age effects of incidence, prevalence, and years lived with disability of anxiety disorders (per 100,000 person-years) and the corresponding 95% CIs by SDI region. Table S7. Relative risk for anxiety disorders incidence, prevalence, and years lived with disability of each period compared with the reference (2002–2006) and the corresponding 95% CIs by SDI region. Table S8. Relative risk for anxiety disorders incidence, prevalence, and years lived with disability of each birth cohort compared with the reference (cohort 1957−1961) and the corresponding 95% CIs by SDI region. Table S9. Fitted longitudinal age effects of incidence, prevalence, and years lived with disability of anxiety disorders (per 100,000 person-years) and the corresponding 95% CIs by WHO region. Table S10. Relative risk for anxiety disorders incidence, prevalence, and years lived with disability of each period compared with the reference (2002–2006) and the corresponding 95% CIs by sex and WHO region. Table S11. Relative risk for anxiety disorders incidence, prevalence, and years lived with disability of each birth cohort compared with the reference (cohort 1957−1961) and the corresponding 95% CIs by sex and WHO region. Figure S1. Global distribution of relative changes in anxiety disorders incidence from 1992 to 2021 and the top 20 countries by age-standardized incidence in 1992 and 2021. Figure S2. Global distribution of relative changes in anxiety disorders prevalence from 1992 to 2021 and the top 20 countries by age-standardized prevalence in 1992 and 2021. Figure S3. Global distribution of relative changes in anxiety disorders years lived with disability from 1992 to 2021 and the top 20 countries by age-standardized years lived with disability in 1992 and 2021. Figure S4. Parameter estimates of age, period, and cohort effects on the incidence of anxiety disorders stratified by sex, globally and across five SDI regions from 1992 to 2021. Figure S5. Parameter estimates of age, period, and cohort effects on the prevalence of anxiety disorders stratified by sex, globally and across five SDI regions from 1992 to 2021. Figure S6. Parameter estimates of age, period, and cohort effects on the years lived with disability of anxiety disorders stratified by sex, globally and across five SDI regions from 1992 to 2021. Figure S7. Parameter estimates of age, period, and cohort effects on the incidence of anxiety disorders, stratified by sex, across the six WHO regions from 1992 to 2021. Figure S8. Parameter estimates of age, period, and cohort effects on the prevalence of anxiety disorders stratified by sex, across the six WHO regions from 1992 to 2021. Figure S9. Parameter estimates of age, period, and cohort effects on the years lived with disability of anxiety disorders stratified by sex, across the six WHO regions from 1992 to 2021.
