# Supplementary material for: Global, Regional, and National Trends in the Burden of Anxiety Disorders From 1992 to 2021: An Age–Period–Cohort Analysis Based on the Global Burden of Disease Study 2021
Source: Depress Anxiety. 2025 Jul 12;2025:4178541. doi: 10.1155/da/4178541 (PMC12276053; doi:10.1155/da/4178541)
Supplement: Supporting Information — Table S1. Annual percentage change for the incidence, prevalence, and years lived with disability of anxiety disorders in each age group stratified by sex from 1992 to 2021 (local drift with 95% CI). Table S2. The number and age-standardized rate (per 100,000) of incidence, prevalence, and years lived with disability of anxiety disorders in 204 countries and territories in 1992 and 2021, and its temporal trends from 1992 to 2021. Table S3. Fitted longitudinal age effects of the incidence, prevalence, and years lived with disability of anxiety disorders (per 100,000 person-years) and the corresponding 95% CIs stratified by sex. Table S4. Relative risks for the incidence, prevalence, and years lived with disability of anxiety disorders for each period compared with the reference period (2002–2006) and the corresponding 95% CIs stratified by sex. Table S5. Relative risk for anxiety disorders incidence, prevalence, and years lived with disability of each birth cohort compared with the reference (cohort 1957−1961) and the corresponding 95% CIs by sex. Table S6. Fitted longitudinal age effects of incidence, prevalence, and years lived with disability of anxiety disorders (per 100,000 person-years) and the corresponding 95% CIs by SDI region. Table S7. Relative risk for anxiety disorders incidence, prevalence, and years lived with disability of each period compared with the reference (2002–2006) and the corresponding 95% CIs by SDI region. Table S8. Relative risk for anxiety disorders incidence, prevalence, and years lived with disability of each birth cohort compared with the reference (cohort 1957−1961) and the corresponding 95% CIs by SDI region. Table S9. Fitted longitudinal age effects of incidence, prevalence, and years lived with disability of anxiety disorders (per 100,000 person-years) and the corresponding 95% CIs by WHO region. Table S10. Relative risk for anxiety disorders incidence, prevalence, and years lived with disability of each period compared with the r [file 4178541.f1.pdf]

Supplementary Appendix

Global, Regional, and National Trends in the Burden of Anxiety Disorders From 1992 to 2021: An Age-Period-Cohort Analysis Based on the Global Burden of Disease Study 2021

Jiali Zhou, MPH[1,2], Shuting Li, MPH[2], Yuan Song, MNS[3], Jiayao Ying, MPH[2], Zeyu Luo, MPH[2], Shiyi Shan, MPH[2], Liying Zhou, PhD[2], Jindian Zha, BD[4], Xin Wang, MPH[5], Peige Song, PhD[2]*, Jianzhong Yang, MD[1*]

[1] Department of Psychiatry, the First Affiliated Hospital, Zhejiang University School of Medicine, Hangzhou, Zhejiang, China
[2] School of Public Health, Zhejiang University School of Medicine, Hangzhou, Zhejiang, China
[3] School of Nursing and Health, Zhengzhou University, Zhengzhou, Henan, China
[4] School of Health Management, Anhui Medical University, Hefei, Anhui, China
[5] Department of Psychiatry, the Second Affiliated Hospital of Kunming Medical University, Kunming, Yunnan, China

This supplementary material has been provided by the authors to give readers additional information about their work.

Contents

Table S1. Annual percentage change for the incidence, prevalence, and years lived with disability of anxiety disorders in each age group stratified by sex from 1992 to 2021 (local drift with 95% CI). .. 4
Table S2. The number and age-standardized rate (per 100,000) of incidence, prevalence, and years lived with disability of anxiety disorders in 204 countries and territories in 1992 and 2021, and its temporal trends from 1992 to 2021. 6
Table S3. Fitted longitudinal age effects of the incidence, prevalence, and years lived with disability of anxiety disorders (per 100,000 person-years) and the corresponding 95% CIs stratified by sex. . 19
Table S4. Relative risks for the incidence, prevalence, and years lived with disability of anxiety disorders for each period compared with the reference period (2002–2006) and the corresponding 95% CIs stratified by sex. 21
Table S5. Relative risk for anxiety disorders incidence, prevalence, and years lived with disability of each birth cohort compared with the reference (cohort 1957-1961) and the corresponding 95% CIs by sex. 22
Table S6. Fitted longitudinal age effects of incidence, prevalence, and years lived with disability of anxiety disorders (per 100,000 person-years) and the corresponding 95% CIs by SDI region. 24
Table S7. Relative risk for anxiety disorders incidence, prevalence, and years lived with disability of each period compared with the reference (2002–2006) and the corresponding 95% CIs by SDI region. 27
Table S8. Relative risk for anxiety disorders incidence, prevalence, and years lived with disability of each birth cohort compared with the reference (cohort 1957-1961) and the corresponding 95% CIs by SDI region. 28
Table S9. Fitted longitudinal age effects of incidence, prevalence, and years lived with disability of anxiety disorders (per 100,000 person-years) and the corresponding 95% CIs by WHO region. 32
Table S10. Relative risk for anxiety disorders incidence, prevalence, and years lived with disability of each period compared with the reference (2002–2006) and the corresponding 95% CIs by sex and WHO region. 35
Table S11. Relative risk for anxiety disorders incidence, prevalence, and years lived with disability of each birth cohort compared with the reference (cohort 1957-1961) and the corresponding 95% CIs by sex and WHO region. 37
Figure S1. Global distribution of relative changes in anxiety disorders incidence from 1992 to 2021 and the top 20 countries by age-standardized incidence in 1992 and 2021. 41
Figure S2. Global distribution of relative changes in anxiety disorders prevalence from 1992 to 2021 and the top 20 countries by age-standardized prevalence in 1992 and 2021. 42
Figure S3. Global distribution of relative changes in anxiety disorders years lived with disability from 1992 to 2021 and the top 20 countries by age-standardized years lived with disability in 1992 and 2021. 43
Figure S4. Parameter estimates of age, period, and cohort effects on the incidence of anxiety disorders stratified by sex, globally and across five SDI regions from 1992 and 2021. 44
Figure S5. Parameter estimates of age, period, and cohort effects on the prevalence of anxiety disorders stratified by sex, globally and across five SDI regions from 1992 and 2021. 45
Figure S6. Parameter estimates of age, period, and cohort effects on the years lived with disability of anxiety disorders stratified by sex, globally and across five SDI regions from 1992 and 2021. 46

Figure S7. Parameter estimates of age, period, and cohort effects on the incidence of anxiety disorders, stratified by sex, across the six WHO regions from 1992 and 2021. 47
Figure S8. Parameter estimates of age, period, and cohort effects on the prevalence of anxiety disorders stratified by sex, across the six WHO regions from 1992 and 2021. 48
Figure S9. Parameter estimates of age, period, and cohort effects on the years lived with disability of anxiety disorders stratified by sex, across the six WHO regions from 1992 and 2021...................... 49

Table S1. Annual percentage change for the incidence, prevalence, and years lived with disability of anxiety disorders in each age group stratified by sex from 1992 to 2021 (local drift with 95% CI).

Age group(years)	Both	Male	Female
**Incidence**			
0-4	0.3738 (0.2193, 0.5286)	0.3082 (0.1565, 0.4601)	0.4169 (0.2450, 0.5891)
5-9	0.2722 (0.2024, 0.3420)	0.2417 (0.1733, 0.3103)	0.2938 (0.2163, 0.3715)
10-14	0.2494 (0.1948, 0.3040)	0.2598 (0.2060, 0.3137)	0.2501 (0.1896, 0.3106)
15-19	0.2661 (0.2144, 0.3177)	0.3164 (0.2650, 0.3677)	0.2463 (0.1894, 0.3033)
20-24	0.2916 (0.2401, 0.3430)	0.3724 (0.3210, 0.4238)	0.2487 (0.1921, 0.3052)
25-29	0.2919 (0.2398, 0.3439)	0.3829 (0.3310, 0.4348)	0.2344 (0.1772, 0.2917)
30-34	0.2657 (0.2120, 0.3194)	0.3400 (0.2868, 0.3932)	0.2104 (0.1511, 0.2698)
35-39	0.2588 (0.2020, 0.3156)	0.3082 (0.2526, 0.3638)	0.2119 (0.1486, 0.2753)
40-44	0.2265 (0.1652, 0.2877)	0.2464 (0.1873, 0.3055)	0.1969 (0.1278, 0.2660)
45-49	0.1600 (0.0917, 0.2282)	0.1503 (0.0858, 0.2148)	0.1543 (0.0761, 0.2326)
50-54	0.1151 (0.0374, 0.1927)	0.0957 (0.0238, 0.1678)	0.1201 (0.0296, 0.2107)
55-59	0.0672 (-0.0212, 0.1558)	0.0631 (-0.0177, 0.1440)	0.0633 (-0.0412, 0.1678)
60-64	0.0065 (-0.0967, 0.1097)	0.0177 (-0.0757, 0.1113)	-0.0077 (-0.1306, 0.1154)
65-69	-0.0040 (-0.1292, 0.1214)	0.0163 (-0.0972, 0.1299)	-0.0332 (-0.1822, 0.1160)
70-74	0.0331 (-0.1299, 0.1963)	0.0528 (-0.0964, 0.2022)	-0.0148 (-0.2069, 0.1777)
75-79	0.0858 (-0.1448, 0.3170)	0.1120 (-0.1024, 0.3268)	0.0127 (-0.2555, 0.2815)
80-84	0.1548 (-0.1964, 0.5072)	0.1835 (-0.1533, 0.5214)	0.0596 (-0.3383, 0.4591)
85-89	0.1740 (-0.4649, 0.8170)	0.2193 (-0.4225, 0.8652)	0.0657 (-0.6337, 0.7701)
90-94	0.1407 (-1.3830, 1.6880)	0.2110 (-1.4091, 1.8576)	0.0337 (-1.5751, 1.6687)
95+	0.0779 (-5.4744, 5.9564)	0.1873 (-6.1032, 6.8993)	-0.0227 (-5.6969, 5.9930)
**Prevalence**			
0-4	0.3898 (0.0087, 0.7724)	0.3054 (-0.0202, 0.6321)	0.4312 (-0.0127, 0.8770)
5-9	0.2322 (0.1228, 0.3417)	0.1713 (0.0769, 0.2658)	0.2634 (0.1369, 0.3900)
10-14	0.1620 (0.0934, 0.2306)	0.1406 (0.0810, 0.2002)	0.1801 (0.1011, 0.2591)
15-19	0.1746 (0.1180, 0.2312)	0.1933 (0.1436, 0.2429)	0.1794 (0.1147, 0.2442)
20-24	0.2377 (0.1853, 0.2900)	0.2887 (0.2423, 0.3351)	0.2221 (0.1626, 0.2816)
25-29	0.2649 (0.2137, 0.3161)	0.3401 (0.2944, 0.3858)	0.2272 (0.1693, 0.2851)
30-34	0.2196 (0.1676, 0.2715)	0.3037 (0.2572, 0.3503)	0.1656 (0.1069, 0.2243)
35-39	0.1714 (0.1174, 0.2255)	0.2541 (0.2056, 0.3025)	0.1064 (0.0453, 0.1675)
40-44	0.1226 (0.0656, 0.1796)	0.2031 (0.1521, 0.2541)	0.0532 (-0.0112, 0.1176)
45-49	0.0601 (-0.0012, 0.1215)	0.1329 (0.0779, 0.1879)	-0.0101 (-0.0793, 0.0593)
50-54	0.0516 (-0.0156, 0.1189)	0.1115 (0.0510, 0.1720)	-0.0128 (-0.0885, 0.0630)
55-59	0.0506 (-0.0231, 0.1243)	0.1172 (0.0504, 0.1841)	-0.0062 (-0.0888, 0.0765)
60-64	-0.0032 (-0.0851, 0.0788)	0.0832 (0.0080, 0.1585)	-0.0563 (-0.1477, 0.0351)
65-69	-0.0730 (-0.1665, 0.0206)	0.0545 (-0.0334, 0.1425)	-0.1157 (-0.2188, -0.0126)
70-74	-0.1292 (-0.2417, -0.0165)	0.0501 (-0.0600, 0.1604)	-0.1441 (-0.2659, -0.0222)
75-79	-0.1425 (-0.2880, 0.0031)	0.0732 (-0.0765, 0.2231)	-0.1407 (-0.2948, 0.0137)
80-84	-0.1388 (-0.3361, 0.0590)	0.1355 (-0.0830, 0.3545)	-0.1277 (-0.3316, 0.0766)
85-89	-0.0987 (-0.4099, 0.2135)	0.2126 (-0.1642, 0.5909)	-0.0926 (-0.4056, 0.2213)
90-94	-0.0526 (-0.6586, 0.5570)	0.2571 (-0.5612, 1.0822)	-0.0675 (-0.6612, 0.5296)

Age group(years)	Both	Male	Female
95+	-0.0658 (-1.5693, 1.4607)	0.2693 (-2.0258, 2.6181)	-0.0937 (-1.5347, 1.3684)
**YLDs**			
0-4	0.5381 (-0.0889, 1.1690)	0.4711 (-0.2778, 1.2256)	0.5774 (-0.0095, 1.1678)
5-9	0.5055 (0.3299, 0.6814)	0.4978 (0.2871, 0.7089)	0.5070 (0.3431, 0.6712)
10-14	0.5164 (0.4086, 0.6244)	0.5767 (0.4471, 0.7065)	0.4824 (0.3818, 0.5831)
15-19	0.5122 (0.4263, 0.5981)	0.6221 (0.5181, 0.7263)	0.4569 (0.3772, 0.5366)
20-24	0.3944 (0.3185, 0.4705)	0.5306 (0.4375, 0.6238)	0.3352 (0.2652, 0.4053)
25-29	0.1725 (0.1013, 0.2437)	0.3138 (0.2258, 0.4019)	0.1210 (0.0557, 0.1864)
30-34	-0.0480 (-0.1172, 0.0213)	0.0883 (0.0024, 0.1742)	-0.0875 (-0.1509, -0.0241)
35-39	-0.1653 (-0.2347, -0.0959)	-0.0369 (-0.1231, 0.0494)	-0.2011 (-0.2645, -0.1376)
40-44	-0.1602 (-0.2314, -0.0889)	-0.0357 (-0.1245, 0.0531)	-0.1962 (-0.2612, -0.1312)
45-49	-0.0549 (-0.1293, 0.0195)	0.0536 (-0.0397, 0.1469)	-0.0932 (-0.1609, -0.0254)
50-54	0.0739 (-0.0063, 0.1542)	0.1844 (0.0827, 0.2863)	0.0310 (-0.0415, 0.1036)
55-59	0.1328 (0.0455, 0.2202)	0.2635 (0.1510, 0.3761)	0.0862 (0.0078, 0.1646)
60-64	0.1055 (0.0092, 0.2018)	0.2524 (0.1261, 0.3788)	0.0734 (-0.0122, 0.1591)
65-69	-0.0190 (-0.1262, 0.0883)	0.1628 (0.0181, 0.3076)	-0.0168 (-0.1110, 0.0775)
70-74	-0.1743 (-0.2980, -0.0504)	0.0567 (-0.1181, 0.2318)	-0.1134 (-0.2204, -0.0063)
75-79	-0.2410 (-0.3959, -0.0859)	-0.0028 (-0.2341, 0.2291)	-0.1328 (-0.2641, -0.0013)
80-84	-0.3624 (-0.5607, -0.1637)	-0.0804 (-0.4002, 0.2405)	-0.2311 (-0.3955, -0.0665)
85-89	-0.4708 (-0.7659, -0.1749)	-0.1504 (-0.6803, 0.3824)	-0.3421 (-0.5802, -0.1034)
90-94	-0.5265 (-1.0698, 0.0198)	-0.2088 (-1.3261, 0.9212)	-0.4152 (-0.8422, 0.0137)
95+	-0.6241 (-1.9070, 0.6756)	-0.2721 (-3.3153, 2.8669)	-0.5434 (-1.5317, 0.4549)

Notes: CI, confidence interval; YLDs, years lived with disability.

Source: Institute for Health Metrics and Evaluation. Used with permission. All rights reserved.

Table S2. The number and age-standardized rate (per 100,000) of incidence, prevalence, and years lived with disability of anxiety disorders in 204 countries and territories in 1992 and 2021, and its temporal trends from 1992 to 2021.

Characteristics	Incidence					Prevalence					YLDs				
	1992		2021		1992–2021	1992		2021		1992–2021	1992		2021		1992–2021
	Number No. (95% UI)	ASR per 100,000 No. (95% UI)	Incident cases No. (95% UI)	ASR per 100,000 No. (95% UI)	Net drift (% per year)	Number No. (95% UI)	ASR per 100,000 No. (95% UI)	Number No. (95% UI)	ASR per 100,000 No. (95% UI)	Net drift (% per year)	Number No. (95% UI)	ASR per 100,000 No. (95% UI)	Number No. (95% UI)	ASR per 100,000 No. (95% UI)	Net drift (% per year)
**Afghanistan**	73771 (59351, 92773)	718.56 (590.16, 912.68)	303477 (212742, 426472)	901.41 (640.42, 1264.13)	0.24 (0.13, 0.36)	460204 (367255, 578717)	4964.98 (4040.2, 6136.87)	1780311 (1256460, 2503673)	6036.08 (4354.2, 8133.31)	0.28 (0.21, 0.35)	54481 (36217, 78129)	580.96 (387.34, 815.49)	212963 (129925, 320564)	708.35 (439.83, 1057.96)	0.25 (0.12, 0.38)
**Albania**	17436 (13957, 22475)	516.89 (418.13, 658.32)	18362 (13440, 24846)	709.56 (521.13, 976.3)	0.31 (0.16, 0.45)	110000 (88598, 135876)	3457.45 (2810.03, 4226.27)	133818 (96251, 178941)	4620.55 (3328.34, 6148.87)	0.29 (0.25, 0.34)	13190 (8764, 18308)	411.18 (278.38, 560.31)	15794 (9780, 24083)	551.63 (337, 835.37)	0.31 (0.17, 0.44)
**Algeria**	199976 (160739, 251154)	707.03 (580.85, 893.65)	363903 (255478, 506148)	812.81 (572.86, 1135.81)	0.20 (0.07, 0.33)	1234136 (981145, 1564847)	4826.2 (3921.64, 5921.68)	2405797 (1696838, 3263059)	5466.45 (3865.66, 7367.72)	0.17 (0.06, 0.27)	148870 (98199, 213049)	575.13 (386.86, 801.32)	286953 (176141, 418472)	651.01 (400.83, 949.52)	0.12 (-0.03, 0.27)
**American Samoa**	292 (236, 371)	567.97 (463.03, 715.53)	340 (241, 475)	657.12 (461.12, 914.38)	0.12 (-0.57, 0.81)	1732 (1379, 2185)	3689.73 (3015.38, 4539.4)	2158 (1519, 2967)	4217.39 (2982.31, 5780.07)	0.11 (-0.23, 0.46)	209 (137, 297)	439.17 (294.15, 614.09)	256 (162, 396)	500.01 (315.14, 773.93)	0.14 (-0.61, 0.90)
**Andorra**	477 (387, 607)	790.56 (646.99, 999.32)	741 (535, 989)	998.24 (711.12, 1309.66)	0.22 (-0.30, 0.74)	3567 (2887, 4397)	5382.31 (4386.84, 6568.39)	5914 (4356, 7936)	6651.34 (4813.12, 8878.86)	0.27 (0.04, 0.49)	425 (285, 594)	643.73 (431.99, 901.42)	697 (449, 1018)	795.58 (490.84, 1175.72)	0.23 (-0.29, 0.74)
**Angola**	59157 (47473, 78029)	555.48 (450.3, 717.04)	211167 (150336, 294186)	646.27 (461.52, 892.58)	0.17 (0, 0.34)	336797 (266942, 427965)	3584.41 (2909.06, 4449.78)	1174074 (815756, 1654588)	4124.19 (2935.43, 5699.3)	0.18 (0.12, 0.24)	40140 (26038, 57268)	421.41 (280.09, 585.67)	140586 (84811, 209874)	486.13 (294.89, 722.52)	0.19 (0.01, 0.38)
**Antigua and Barbuda**	406 (330, 524)	642.02 (526.03, 818.54)	721 (508, 975)	792.31 (554.16, 1076.56)	0.15 (-0.46, 0.77)	2659 (2133, 3327)	4309.92 (3507.57, 5299.49)	5063 (3589, 6863)	5216.53 (3684.36, 7121.99)	0.16 (-0.11, 0.43)	317 (209, 443)	512.4 (342.97, 709.8)	599 (365, 897)	619.69 (374.96, 926.73)	0.15 (-0.42, 0.71)
**Argentina**	244908 (197962, 308537)	718.19 (582.31, 909.4)	409075 (285015, 568040)	908.12 (636.59, 1259.62)	0.16 (0.01, 0.32)	1713779 (1390706, 2118557)	5099.36 (4132.15, 6308.87)	3001028 (2169144, 4008612)	6334.57 (4550.53, 8460.44)	0.14 (0.09, 0.19)	204373 (137400, 280320)	607.54 (407.08, 830.68)	356341 (220745, 525463)	754.9 (469.58, 1119.09)	0.14 (0.08, 0.19)
**Armenia**	13764 (11250, 17581)	397.76 (326.57, 504.12)	16232 (11596, 22236)	550.71 (393.81, 759.28)	0.29 (0.15, 0.44)	85316 (69092, 105552)	2525.76 (2058.37, 3092.83)	107596 (78605, 143725)	3403.64 (2459.84, 4599.42)	0.25 (0.20, 0.31)	10207 (6777, 14038)	300.95 (201.92, 413.83)	12748 (7924, 19716)	406.95 (253.15, 619.9)	0.28 (0.10, 0.46)
**Australia**	137227 (111280, 172267)	791.15 (648.01, 993.63)	207852 (150112, 282612)	876.26 (630.78, 1204.07)	0.13 (-0.24, 0.50)	1008926 (842381, 1216783)	5550.95 (4644.38, 6753)	1633504 (1161019, 2191960)	6176.37 (4440.58, 8286.46)	0.29 (0.15, 0.43)	119425 (81916, 164945)	659 (447.98, 910.01)	191842 (120046, 279870)	731.99 (445.85, 1057.56)	0.28 (0.13, 0.43)
**Austria**	59150 (48400, 74193)	813.79 (669.67, 1022.01)	78883 (57464, 107996)	1029.45 (739.43, 1410.83)	0.27 (0.16, 0.37)	473620 (387186, 574437)	5742.41 (4703.41, 7081.69)	647994 (463506, 843718)	7060.07 (4935.03, 9457.65)	0.21 (0.19, 0.23)	55903 (37484, 76149)	684.77 (448.13, 958.64)	76410 (46751, 110752)	846.35 (521.78, 1246.69)	0.22 (0.17, 0.28)
**Azerbaijan**	27805 (22311, 35695)	367.52 (299.5, 461.61)	52395 (37748, 73607)	484.89 (350.03, 692.33)	0.21 (0.10, 0.31)	163925 (131560, 205298)	2268.93 (1844.51, 2795.98)	321452 (228194, 439451)	2906.05 (2077.55, 3989.9)	0.13 (0.08, 0.18)	19696 (13129, 27977)	270.77 (183.99, 375.85)	38386 (23020, 58316)	347.4 (210.77, 524.29)	0.13 (0.01, 0.25)
**Bahamas**	1779 (1439, 2289)	639.22 (521.4, 804.64)	3318 (2342, 4644)	819.68 (578.72, 1149.08)	0.29 (-0.07, 0.64)	11367 (9129, 14311)	4290.46 (3496.18, 5289.78)	22690 (16648, 31390)	5388.01 (3928.96, 7396.89)	0.24 (0.09, 0.40)	1364 (904, 1909)	510.91 (343.67, 706.71)	2691 (1691, 3951)	640.07 (404.12, 941.5)	0.21 (-0.14, 0.57)
**Bahrain**	4117 (3324, 5333)	728.75 (598.11, 925.86)	13861 (9613, 18860)	884.63 (622.2, 1191.85)	0.15 (-0.10, 0.41)	26630 (20985, 33695)	4927.8 (3951.92, 6064.31)	93622 (66063, 123860)	5719.99 (4016.02, 7508.78)	0.02 (-0.15, 0.20)	3204 (2113, 4608)	586.85 (389.93, 812.72)	11201 (6731, 16894)	682.81 (412.66, 1044.99)	0.02 (-0.28, 0.32)
**Bangladesh**	499093 (408927, 627149)	480.66 (394.11, 592.83)	1024645 (736582, 1419297)	601.28 (433.55, 829.02)	0.32 (0.11, 0.52)	2744159 (2281482, 3285655)	2992.73 (2500.72, 3550.59)	6192235 (4450867, 8282519)	3700.86 (2656.44, 4930.4)	0.40 (0.31, 0.50)	327224 (220350, 452181)	351.79 (239.27, 480.94)	733261 (436672, 1111067)	436.65 (259.09, 658.34)	0.42 (0.32, 0.52)
**Barbados**	1638 (1341, 2083)	636.88 (522.4, 804.66)	2367 (1769, 3186)	822.85 (601.73, 1118.58)	0.19 (-0.15, 0.53)	11278 (9114, 13740)	4280.34 (3485.98, 5191.87)	17422 (12805, 23277)	5386.46 (3987.96, 7257.64)	0.20 (0.07, 0.33)	1343 (903, 1860)	510.23 (342.18, 702.22)	2049 (1291, 3049)	640.99 (403.44, 965.4)	0.20 (-0.10, 0.50)

Characteristics	Incidence					Prevalence					YLDs				
	1992		2021		1992–2021	1992		2021		1992–2021	1992		2021		1992–2021
	Number No. (95% UI)	ASR per 100,000 No. (95% UI)	Incident cases No. (95% UI)	ASR per 100,000 No. (95% UI)	Net drift (% per year)	Number No. (95% UI)	ASR per 100,000 No. (95% UI)	Number No. (95% UI)	ASR per 100,000 No. (95% UI)	Net drift (% per year)	Number No. (95% UI)	ASR per 100,000 No. (95% UI)	Number No. (95% UI)	ASR per 100,000 No. (95% UI)	Net drift (% per year)
**Belarus**	55621 (45308, 70094)	528.85 (428.45, 669.97)	70157 (49983, 93516)	810.42 (584.55, 1091.2)	0.29 (0.18, 0.40)	390014 (318399, 480523)	3529.39 (2851.75, 4363.62)	500704 (359309, 678776)	5150.44 (3790.93, 6944.68)	0.28 (0.23, 0.33)	46075 (30877, 63428)	419.55 (280.93, 580.35)	58811 (36700, 86741)	614.76 (383.21, 917.54)	0.29 (0.22, 0.35)
**Belgium**	71980 (58627, 90059)	768.59 (628.7, 964.22)	93758 (68177, 124765)	932.2 (663.22, 1248.46)	0.28 (0.19, 0.37)	537008 (442857, 648712)	5124.53 (4109.89, 6255.42)	708043 (516481, 952115)	6092.43 (4482.38, 8329.08)	0.29 (0.23, 0.34)	63435 (43249, 86947)	611.56 (411.81, 843.57)	83189 (52947, 122726)	727.63 (456.48, 1093.25)	0.27 (0.22, 0.33)
**Belize**	1217 (978, 1573)	629.88 (514.28, 797.86)	3478 (2467, 4847)	768.63 (552.06, 1058.94)	0.25 (-0.18, 0.68)	7146 (5769, 9011)	4154 (3376.59, 5111.83)	22099 (15643, 30687)	5013.49 (3564.01, 6874.37)	0.23 (0.06, 0.41)	860 (575, 1232)	494.95 (333.11, 684.88)	2641 (1655, 4137)	595.88 (375.77, 924.43)	0.22 (-0.22, 0.65)
**Benin**	23862 (18922, 30225)	472.39 (381.48, 590.7)	71945 (51497, 100221)	524.79 (375.62, 715.39)	0.13 (-0.03, 0.29)	129773 (101695, 166708)	2953.33 (2383.84, 3632.11)	400698 (278058, 558584)	3264.96 (2291.77, 4417.88)	0.15 (0.09, 0.21)	15532 (10281, 22645)	348.62 (234.29, 489.39)	48348 (28449, 75248)	388.52 (240.58, 600.68)	0.20 (0, 0.39)
**Bermuda**	398 (319, 507)	638.11 (517.74, 806.42)	468 (330, 644)	803.01 (561.27, 1127.61)	0.18 (-0.41, 0.77)	2791 (2245, 3487)	4275.86 (3483.72, 5268.62)	3599 (2554, 4770)	5259.93 (3629.08, 7087.96)	0.15 (-0.11, 0.41)	333 (224, 460)	511.24 (345.02, 706.4)	423 (252, 619)	629.07 (373.45, 930.25)	0.21 (-0.34, 0.77)
**Bhutan**	2999 (2393, 3877)	490.83 (399.23, 618.15)	4154 (2933, 5729)	517.39 (363.19, 710.07)	0.15 (-0.15, 0.46)	16794 (13412, 21286)	3125.98 (2517.09, 3834.82)	25613 (18057, 34496)	3247.23 (2301.02, 4295.16)	0.11 (-0.07, 0.30)	2009 (1310, 2846)	368.51 (245.73, 506.1)	3050 (1902, 4557)	384.77 (240.92, 576.27)	0.08 (-0.26, 0.42)
**Bolivia (Plurinational State of)**	49675 (39790, 64548)	748.74 (604.26, 954.64)	135726 (97143, 187970)	1109.65 (793.81, 1527.53)	0.42 (0.29, 0.55)	314847 (252677, 401469)	5325.9 (4314.76, 6623.02)	905796 (650760, 1217550)	7571.02 (5489.71, 10192.49)	0.40 (0.29, 0.51)	37686 (24831, 52593)	630.8 (422.85, 864.1)	108076 (68383, 163049)	899.89 (569.15, 1353.07)	0.41 (0.28, 0.53)
**Bosnia and Herzegovina**	23676 (19144, 29917)	509.2 (414.27, 645.69)	21933 (15442, 30220)	702.29 (496.86, 959.54)	0.23 (0.12, 0.34)	160846 (131328, 197150)	3412.1 (2797.52, 4165.2)	162403 (117097, 216773)	4517.48 (3310.9, 6032.4)	0.19 (0.14, 0.23)	19112 (12894, 26390)	405.07 (274.03, 558.63)	18933 (11457, 28322)	536.88 (322.78, 789.91)	0.18 (0.06, 0.30)
**Botswana**	7604 (6081, 9717)	540.09 (438.5, 684.41)	18447 (12974, 26054)	728.21 (517.75, 1011.56)	0.18 (-0.03, 0.40)	43537 (34379, 55348)	3485.48 (2803.62, 4297.04)	112645 (80804, 155609)	4575.9 (3289.5, 6264.97)	0.23 (0.08, 0.38)	5216 (3356, 7532)	411.95 (273.19, 571.64)	13333 (8359, 20849)	538.26 (339.81, 828.07)	0.19 (-0.06, 0.43)
**Brazil**	1359823 (1119144, 1645727)	857.11 (708.7, 1034.56)	2705000 (2218152, 3335224)	1209.77 (993.15, 1503.98)	0.39 (-0.49, 1.28)	8867151 (7700311, 10151121)	6010.6 (5255.59, 6825.44)	21248815 (18160824, 24695181)	9007.36 (7692.54, 10470.78)	0.71 (0.35, 1.06)	1050795 (729333, 1445344)	705.58 (487.21, 958.73)	2488938 (1724559, 3388188)	1058.67 (732.51, 1442.26)	0.72 (0.33, 1.11)
**Brunei Darussalam**	1345 (1090, 1717)	468.97 (385.65, 586.36)	2516 (1759, 3413)	542.72 (379.68, 730.08)	0.05 (-0.41, 0.52)	7799 (6197, 9870)	2882.84 (2361.01, 3518.18)	15884 (11188, 21988)	3306.36 (2361.03, 4538.2)	0.09 (-0.10, 0.29)	947 (627, 1363)	346.29 (232.22, 487.23)	1911 (1193, 2789)	397.17 (249.92, 587.8)	0.13 (-0.33, 0.59)
**Bulgaria**	44559 (35942, 55188)	516.06 (417.43, 649.87)	48862 (35248, 66576)	795.06 (577.02, 1093.33)	0.32 (0.14, 0.50)	324015 (265628, 397006)	3451.22 (2812.94, 4245.49)	365854 (261410, 496255)	5050.24 (3579.46, 6952.15)	0.31 (0.10, 0.53)	38138 (26155, 53455)	410.21 (274.69, 564.91)	42657 (26018, 64406)	601.39 (366.25, 912.68)	0.31 (0.10, 0.52)
**Burkina Faso**	45601 (36277, 57507)	466.84 (378.52, 582.36)	115951 (83372, 161284)	507.72 (366.97, 701.02)	0.11 (-0.02, 0.24)	248636 (196441, 318891)	2912.04 (2334.62, 3603.99)	643521 (441153, 900698)	3146.77 (2215.14, 4314.47)	0.11 (0.06, 0.16)	29702 (19767, 42921)	343.84 (233.85, 480.12)	77601 (46357, 114193)	375.07 (226.34, 548.75)	0.17 (0, 0.34)
**Burundi**	29586 (23627, 38539)	538.71 (431, 686.87)	86816 (60139, 121613)	652.1 (457.89, 902.69)	0.22 (0.03, 0.41)	167208 (132461, 214148)	3493.04 (2813.68, 4317.6)	489121 (337013, 668723)	4156.98 (2929.75, 5670.37)	0.18 (0.11, 0.25)	19996 (13084, 27966)	412.65 (273.73, 572.27)	58881 (35520, 90399)	493.98 (307.15, 731.47)	0.17 (-0.03, 0.37)
**Cabo Verde**	1721 (1371, 2134)	467.36 (376.9, 584.78)	3324 (2359, 4512)	569.42 (405.54, 765.18)	0.16 (-0.23, 0.56)	9699 (7687, 12519)	2916.8 (2348.89, 3598.6)	20510 (14436, 28333)	3496.21 (2466.81, 4835.12)	0.18 (0.03, 0.32)	1170 (776, 1669)	348.38 (233.35, 487.11)	2461 (1509, 3809)	418.05 (258.97, 644.38)	0.19 (-0.19, 0.56)
**Cambodia**	59872 (47934, 75923)	578.06 (469.05, 732.79)	125733 (89933, 174384)	717.22 (510.53, 986.74)	0.16 (0.05, 0.27)	348285 (282034, 434234)	3881.91 (3192.65, 4755.46)	795682 (564266, 1083812)	4701.53 (3373.95, 6348.07)	0.18 (0.14, 0.22)	41592 (27706, 58622)	457.02 (309.21, 638.64)	95272 (56653, 142575)	559.24 (341.64, 829.83)	0.22 (0.09, 0.34)

Characteristics	Incidence					Prevalence					YLDs				
	1992		2021		1992–2021	1992		2021		1992–2021	1992		2021		1992–2021
	Number No. (95% UI)	ASR per 100,000 No. (95% UI)	Incident cases No. (95% UI)	ASR per 100,000 No. (95% UI)	Net drift (% per year)	Number No. (95% UI)	ASR per 100,000 No. (95% UI)	Number No. (95% UI)	ASR per 100,000 No. (95% UI)	Net drift (% per year)	Number No. (95% UI)	ASR per 100,000 No. (95% UI)	Number No. (95% UI)	ASR per 100,000 No. (95% UI)	Net drift (% per year)
**Cameroon**	52114 (41506, 65335)	473.56 (382.7, 589.2)	173840 (116060, 238144)	527.94 (357.51, 722.33)	0.11 (-0.01, 0.23)	290075 (230128, 369397)	2961.56 (2390.48, 3642.56)	977912 (671005, 1339982)	3274.42 (2257.32, 4490.83)	0.13 (0.08, 0.17)	34711 (22765, 49493)	349.79 (233.82, 488.22)	117961 (70656, 174269)	389.7 (237.49, 580.17)	0.14 (-0.01, 0.28)
**Canada**	187297 (156091, 223564)	649.81 (546.59, 767.08)	262563 (192904, 364744)	751.19 (552.91, 1053.36)	0.30 (-0.03, 0.64)	1339096 (1220404, 1469147)	4405.44 (4022.71, 4834.15)	1971166 (1430067, 2693930)	4946.13 (3597.11, 6732.41)	0.30 (0.13, 0.47)	159046 (110663, 215534)	524.88 (367.57, 711.44)	231182 (149052, 349906)	586.81 (376.45, 900.19)	0.29 (0.12, 0.46)
**Central African Republic**	15803 (12707, 20521)	566.07 (456.97, 724.43)	34991 (24554, 50733)	627.23 (455.14, 892.47)	0.18 (0, 0.37)	91722 (73347, 115984)	3708.09 (3002.86, 4564.7)	205075 (144218, 286537)	4106.39 (2929.94, 5621.11)	0.25 (0.12, 0.38)	10821 (7094, 14978)	431.15 (289.68, 589.05)	24423 (14594, 37392)	481.84 (292.81, 734.43)	0.26 (0.03, 0.49)
**Chad**	29352 (23649, 37043)	476.17 (386.34, 598.37)	89996 (63613, 127839)	512.32 (368.7, 709.69)	0.16 (0.01, 0.31)	162162 (127539, 206723)	2981.74 (2427.36, 3691.19)	487629 (340263, 701271)	3203.69 (2286.12, 4398.01)	0.15 (0.09, 0.20)	19371 (12653, 28018)	351.92 (236.81, 489.55)	58821 (34060, 87350)	380.22 (229.09, 562.66)	0.19 (0.01, 0.38)
**Chile**	98190 (81693, 117461)	697.27 (583.46, 830.38)	161859 (116450, 214966)	896.89 (638.05, 1186.61)	0.34 (0.15, 0.54)	737882 (670849, 811440)	5390.02 (4909.73, 5919.46)	1312901 (930603, 1770036)	6621.57 (4710.46, 8939.41)	0.28 (0.20, 0.35)	87773 (62514, 117420)	638.64 (453.26, 853.75)	154389 (96454, 227745)	783.87 (488.52, 1147.6)	0.27 (0.19, 0.36)
**China**	6512477 (5354111, 7929665)	531.67 (447.77, 632.97)	7733625 (6459019, 9303681)	546.51 (454.19, 662.14)	-0.28 (-1.05, 0.49)	41376172 (35586626, 47873710)	3407.47 (2955.8, 3916.09)	53132387 (45544682, 61644247)	3481.74 (2976.2, 4044.48)	-0.48 (-0.70, -0.26)	5001681 (3485901, 6882218)	409.04 (285.79, 559.59)	6314450 (4367592, 8566618)	418.95 (291.52, 573.17)	-0.48 (-0.72, -0.23)
**Colombia**	233400 (187586, 300235)	669.85 (547.29, 848.19)	413023 (296578, 578513)	834.58 (598.24, 1176.2)	-0.04 (-0.31, 0.24)	1493248 (1175887, 1854733)	4582.01 (3677.67, 5619.97)	2902511 (2044629, 4004027)	5600.77 (3929.9, 7774.08)	-0.21 (-0.35, -0.08)	179374 (118653, 252024)	545.51 (361.34, 754.56)	346374 (212262, 536353)	669.9 (408.53, 1027.02)	-0.20 (-0.34, -0.06)
**Comoros**	2477 (1931, 3210)	532.09 (427.56, 680.22)	4971 (3600, 7080)	642.55 (469.7, 893.49)	0.17 (-0.18, 0.51)	14050 (11184, 17789)	3426.23 (2764.8, 4230.7)	30330 (22195, 42566)	4100.14 (3013.58, 5730.83)	0.19 (-0.02, 0.41)	1690 (1112, 2387)	406.79 (271.73, 560.46)	3633 (2223, 5589)	488.01 (301.62, 751.51)	0.29 (-0.04, 0.61)
**Congo**	13719 (10990, 17752)	542.03 (441.31, 690.27)	35463 (24848, 49588)	628.43 (441.23, 867.68)	0.15 (-0.01, 0.32)	78756 (62741, 100330)	3484.87 (2809.32, 4305.61)	209237 (149025, 285795)	3971.74 (2821.54, 5317.72)	0.10 (-0.05, 0.25)	9393 (6105, 13413)	409.95 (278.1, 570.97)	24911 (15659, 38715)	468.24 (298.84, 728.05)	0.12 (-0.09, 0.34)
**Cook Islands**	111 (90, 141)	567.42 (460.86, 716.77)	117 (85, 162)	666.15 (470.46, 924.86)	0.22 (-0.82, 1.27)	680 (554, 843)	3676.35 (3011.66, 4478.46)	805 (597, 1079)	4290.29 (3165.51, 5779.36)	0.11 (-0.35, 0.57)	82 (54, 119)	438.93 (293.5, 623.35)	95 (59, 147)	510.65 (318.11, 780.65)	0.12 (-1.04, 1.29)
**Costa Rica**	20131 (16266, 25953)	610.05 (496.6, 766.89)	37574 (26823, 52694)	782.43 (555.19, 1105.45)	0.23 (0.03, 0.42)	123799 (99162, 156930)	4025.98 (3275.56, 4925.03)	254843 (179998, 347059)	5075.69 (3630.1, 6925.65)	0.30 (0.25, 0.34)	14900 (9679, 20858)	480.37 (322.07, 669.3)	30305 (18634, 45117)	605.61 (371.33, 902.18)	0.30 (0.17, 0.43)
**Croatia**	62423 (49921, 77975)	475.34 (387.5, 597.53)	26261 (18841, 35416)	667.49 (470.56, 906.6)	0.11 (-0.03, 0.25)	344976 (271313, 441484)	2953.01 (2383.6, 3654.26)	201138 (141138, 277284)	4341.16 (3059.41, 5816.23)	0.13 (0.07, 0.19)	41204 (27153, 59177)	347.35 (234.46, 486.36)	23471 (14813, 35962)	517.78 (325.99, 800.6)	0.18 (0, 0.36)
**Cuba**	25039 (20307, 31282)	508.16 (412.79, 643.76)	87306 (62434, 118811)	812.43 (590.98, 1084.57)	0.23 (0.14, 0.32)	179735 (145822, 218962)	3417.29 (2781, 4155.87)	642974 (461308, 847067)	5297.69 (3778.55, 7067.04)	0.20 (0.16, 0.23)	21188 (14285, 29078)	405.55 (271.88, 558.88)	75710 (49515, 109926)	631.6 (406.16, 920.39)	0.20 (0.10, 0.30)
**Cyprus**	72153 (58423, 91939)	636.84 (523.38, 807.77)	12225 (8824, 16646)	997.44 (713.44, 1377.81)	0.17 (0.04, 0.30)	489648 (394690, 609437)	4234.72 (3443.64, 5195.74)	98305 (71680, 132895)	6986.37 (5007.9, 9390.65)	0.20 (0.17, 0.22)	58327 (38481, 81195)	504.07 (336.12, 697.96)	11647 (7363, 17033)	836.76 (531.57, 1247.72)	0.19 (0.13, 0.26)
**Czechia**	6736 (5529, 8600)	835.62 (681.11, 1060.74)	63927 (45526, 86828)	644.38 (465.31, 891.59)	0.21 (0.05, 0.38)	49227 (40203, 60495)	5940.58 (4877.64, 7370.23)	468404 (343329, 630912)	4094.69 (3027.71, 5509.17)	0.17 (0.03, 0.30)	5875 (3939, 8152)	709.88 (474.27, 990.33)	54595 (35008, 82260)	487.52 (308.18, 754.34)	0.21 (0.02, 0.40)
**Cote d'Ivoire**	51386 (41873, 64643)	490.55 (401.82, 623.13)	147787 (102081, 210176)	517.81 (368.33, 729.26)	0.20 (0.07, 0.34)	361861 (293998, 440045)	3237.35 (2629.97, 3946.52)	836284 (585806, 1169827)	3210.76 (2302.58, 4348.69)	0.13 (0.09, 0.16)	42579 (28861, 58502)	383.82 (256.14, 532.27)	100614 (62003, 153518)	381.67 (236.2, 585.09)	0.14 (0.07, 0.20)

Characteristics	Incidence					Prevalence					YLDs				
	1992		2021		1992–2021	1992		2021		1992–2021	1992		2021		1992–2021
	Number No. (95% UI)	ASR per 100,000 No. (95% UI)	Incident cases No. (95% UI)	ASR per 100,000 No. (95% UI)	Net drift (% per year)	Number No. (95% UI)	ASR per 100,000 No. (95% UI)	Number No. (95% UI)	ASR per 100,000 No. (95% UI)	Net drift (% per year)	Number No. (95% UI)	ASR per 100,000 No. (95% UI)	Number No. (95% UI)	ASR per 100,000 No. (95% UI)	Net drift (% per year)
**Democratic People's Republic of Korea**	115234 (93300, 144024)	548.5 (446.75, 677.87)	149845 (108487, 198588)	577 (412.21, 771.08)	0.05 (-0.09, 0.19)	737839 (603477, 899200)	3563.89 (2946.49, 4321.89)	1029902 (732278, 1375071)	3681.9 (2593.62, 4932.47)	-0.03 (-0.04, -0.01)	89047 (59864, 125844)	428.12 (287.81, 600.3)	123299 (76962, 183160)	443.46 (276.21, 666.18)	-0.01 (-0.07, 0.05)
**Democratic Republic of the Congo**	212240 (169360, 273793)	548.04 (448, 701.64)	549426 (390081, 769536)	599.99 (434.33, 830.01)	0.10 (0.02, 0.18)	1204326 (959322, 1535162)	3529.79 (2846.48, 4379.29)	3133492 (2202032, 4402054)	3828.97 (2701.67, 5320.81)	0.16 (0.12, 0.2)	141992 (92507, 201617)	410.29 (275.04, 566.51)	373287 (226870, 577593)	449.95 (277.3, 688.95)	0.20 (0.10, 0.30)
**Denmark**	36387 (29839, 45769)	759.02 (624.7, 949.67)	47623 (34881, 61998)	932.94 (674.89, 1253.67)	0.20 (0.08, 0.31)	279707 (228275, 341491)	5137.95 (4188.93, 6370.75)	366452 (265044, 487315)	6154.28 (4472.94, 8223.4)	0.15 (0.13, 0.18)	32944 (22661, 44903)	611.89 (412.71, 844.93)	43128 (27391, 61821)	734.87 (469.29, 1069.96)	0.18 (0.11, 0.24)
**Djibouti**	2581 (2030, 3426)	517.54 (416.6, 666.28)	7958 (5560, 11014)	603.45 (423.65, 833.77)	0.19 (-0.13, 0.52)	14611 (11455, 18952)	3284.13 (2631.11, 4043.81)	47590 (34367, 66494)	3784.33 (2750.17, 5203.29)	0.14 (-0.03, 0.32)	1766 (1131, 2503)	391.29 (259.63, 542.07)	5726 (3470, 8649)	451.77 (274.79, 678.59)	0.16 (-0.20, 0.53)
**Dominica**	452 (369, 575)	633.18 (516.7, 798.54)	547 (394, 742)	800.58 (575.67, 1090.1)	0.26 (-0.35, 0.88)	2906 (2358, 3575)	4263.45 (3470.25, 5198.64)	3766 (2689, 5077)	5226.34 (3713.46, 7068.88)	0.13 (-0.15, 0.41)	347 (232, 484)	506.67 (343.54, 697.78)	444 (273, 674)	619.32 (376.91, 940.49)	0.23 (-0.36, 0.82)
**Dominican Republic**	48870 (38947, 62750)	652.85 (529.29, 823.19)	85381 (60541, 118172)	759.58 (541.3, 1048.67)	0.18 (0.08, 0.27)	304243 (244850, 380290)	4398.38 (3557.53, 5351.48)	567510 (411276, 764096)	5038.15 (3656.08, 6797.53)	0.20 (0.16, 0.23)	36528 (24297, 51813)	523.28 (349.63, 720.94)	67529 (41190, 101476)	598.58 (364.2, 900.92)	0.19 (0.08, 0.29)
**Ecuador**	76478 (60815, 99620)	724 (586.92, 924.75)	178406 (128400, 250661)	962.19 (692.17, 1350.06)	0.29 (0.13, 0.45)	484982 (391830, 616842)	5035.92 (4095.12, 6200.06)	1200624 (867990, 1640543)	6522.84 (4731.25, 8874.14)	0.35 (0.28, 0.42)	58329 (38821, 81970)	600.01 (405.96, 826.24)	143129 (91995, 218164)	776.17 (499.28, 1179.17)	0.35 (0.27, 0.43)
**Egypt**	395113 (316495, 498778)	660.5 (536.05, 833.25)	899197 (636150, 1243266)	813.5 (581.6, 1118.19)	0.39 (-0.26, 1.05)	2401285 (1900809, 3033229)	4329.7 (3482.69, 5339.75)	5481791 (3911372, 7472414)	5213.93 (3730.07, 7105.72)	0.36 (0.08, 0.65)	288895 (191023, 412332)	515.88 (350.28, 719.89)	658669 (380569, 983989)	622.28 (363.72, 915.86)	0.36 (0.05, 0.67)
**El Salvador**	33917 (27027, 43454)	622.44 (500.85, 794.51)	51254 (37000, 70819)	782.34 (565.84, 1087.03)	0.28 (0.11, 0.44)	206826 (165371, 259314)	4142.57 (3388.59, 5069.16)	336754 (236885, 455122)	5162.51 (3637.01, 6934.1)	0.34 (0.31, 0.37)	24707 (16205, 35182)	490.2 (322.74, 675.73)	40113 (24743, 58924)	613.95 (378.88, 904.11)	0.35 (0.25, 0.45)
**Equatorial Guinea**	2400 (1904, 3063)	549.18 (447.59, 697.92)	9763 (6741, 13744)	607.55 (427.58, 841.85)	0.07 (-0.29, 0.43)	13644 (10799, 17408)	3553.85 (2865.69, 4455.15)	55896 (38486, 79115)	3867.75 (2705.61, 5289.87)	0.10 (-0.05, 0.25)	1611 (1045, 2255)	413.86 (275.72, 563.67)	6690 (4154, 10315)	455.18 (290.27, 695)	0.13 (-0.19, 0.46)
**Eritrea**	16642 (13208, 21516)	543.12 (439.43, 691.96)	41862 (29669, 57797)	615.87 (441.45, 843.45)	0.15 (-0.06, 0.36)	94296 (74881, 120074)	3561 (2870.87, 4389.69)	247153 (171694, 335381)	4000.05 (2849.87, 5445.09)	0.14 (-0.02, 0.30)	11175 (7272, 15862)	416.64 (278.22, 582.98)	29668 (18170, 45765)	473.86 (302.31, 721.56)	0.13 (-0.10, 0.37)
**Estonia**	8026 (6553, 10067)	523.8 (427.78, 672.11)	8521 (5993, 11757)	715.19 (500.85, 1000.26)	0.18 (0.02, 0.33)	56692 (46335, 69283)	3477.87 (2842.59, 4265.9)	62597 (44250, 86952)	4552.21 (3174.23, 6369.6)	0.12 (0.07, 0.18)	6667 (4479, 9189)	412.3 (276.01, 569.92)	7322 (4570, 10737)	542.88 (331.34, 795.84)	0.13 (-0.04, 0.30)
**Eswatini**	4548 (3624, 5810)	540.17 (437.58, 688.78)	9801 (6900, 13609)	794.24 (565.72, 1104.48)	0.28 (-0.04, 0.59)	25342 (19890, 32094)	3478.24 (2824.17, 4303.87)	56785 (40185, 77501)	4947.7 (3511.5, 6715.79)	0.33 (0.14, 0.52)	3057 (1998, 4419)	413.15 (275.63, 572.39)	6736 (4239, 10250)	579.79 (369.69, 863.48)	0.25 (-0.02, 0.52)
**Ethiopia**	294310 (240053, 360410)	569.58 (471.61, 691.85)	715202 (560057, 888280)	642.65 (510.28, 792.27)	0.06 (-0.14, 0.26)	1661970 (1428161, 1952780)	3706.85 (3187.34, 4304.39)	4056308 (3329425, 4941349)	4057.26 (3364.81, 4867.17)	-0.02 (-0.20, 0.16)	198278 (136770, 273430)	435.99 (302.16, 590.92)	487508 (325297, 695795)	481.07 (323.2, 676.57)	0.01 (-0.18, 0.20)
**Fiji**	4533 (3663, 5824)	569.13 (465.9, 716.6)	6518 (4544, 9049)	690.1 (481.63, 956.59)	0.17 (-0.10, 0.44)	27082 (21449, 34119)	3709.36 (3001.17, 4531.2)	40874 (29028, 56273)	4421.75 (3159.72, 6048.24)	0.16 (0.01, 0.31)	3263 (2156, 4673)	440.77 (298.62, 611.16)	4874 (3062, 7514)	525.09 (331.87, 806.05)	0.13 (-0.16, 0.42)
**Finland**	31251 (25535, 38533)	668.12 (541.25, 824.79)	37829 (27716, 52513)	786.66 (557.43, 1096.04)	0.55 (0.38, 0.71)	208130 (174592, 251492)	4089.44 (3384.84, 4960.01)	268479 (193847, 373019)	4803.26 (3445.06, 6780.44)	0.73 (0.66, 0.80)	24670 (16954, 33970)	489.47 (334.77, 672.79)	31505 (19212, 47925)	574.29 (351.39, 876.55)	0.74 (0.66, 0.82)

Characteristics	Incidence					Prevalence					YLDs				
	1992		2021		1992–2021	1992		2021		1992–2021	1992		2021		1992–2021
	Number No. (95% UI)	ASR per 100,000 No. (95% UI)	Incident cases No. (95% UI)	ASR per 100,000 No. (95% UI)	Net drift (% per year)	Number No. (95% UI)	ASR per 100,000 No. (95% UI)	Number No. (95% UI)	ASR per 100,000 No. (95% UI)	Net drift (% per year)	Number No. (95% UI)	ASR per 100,000 No. (95% UI)	Number No. (95% UI)	ASR per 100,000 No. (95% UI)	Net drift (% per year)
**France**	468696 (384094, 586200)	851.3 (699.04, 1058.67)	619730 (453442, 846629)	1076.13 (776.28, 1475.68)	0.18 (0.04, 0.33)	3825573 (3157580, 4603506)	6280.16 (5137.91, 7614.81)	5196593 (3795179, 6928987)	7694.09 (5574.2, 10200.2)	0.07 (0.02, 0.12)	451485 (309345, 609805)	747.25 (505.26, 1025.89)	609422 (381940, 896794)	916.74 (561.91, 1333.63)	0.07 (0.02, 0.13)
**Gabon**	5600 (4451, 7297)	552.63 (448.23, 709.8)	12684 (8944, 17975)	671.3 (476.7, 953.92)	0.20 (-0.02, 0.41)	33028 (26252, 42064)	3574.91 (2896.67, 4436.86)	75907 (53948, 106466)	4274.37 (3050.91, 5958.42)	0.19 (0.07, 0.31)	3924 (2576, 5527)	420.63 (281.7, 583.25)	9009 (5664, 13577)	502.79 (320.42, 756.89)	0.22 (-0.03, 0.47)
**Gambia**	5065 (4043, 6396)	478.93 (390.19, 606.29)	13810 (9546, 19219)	552.4 (392.51, 757.07)	0.17 (-0.10, 0.44)	28223 (21758, 35474)	2999.43 (2436.29, 3690.63)	78503 (54955, 107392)	3434.07 (2446.4, 4671.44)	0.16 (-0.01, 0.34)	3399 (2198, 4906)	356.3 (237.29, 492.21)	9428 (5768, 14271)	406.92 (254.21, 612.43)	0.13 (-0.17, 0.43)
**Georgia**	20274 (16417, 25332)	367.67 (298.44, 459.21)	17051 (11909, 23348)	491.68 (348.49, 681.89)	0.16 (-0.01, 0.32)	128410 (104613, 157270)	2269.66 (1853.95, 2772.28)	110494 (78394, 151459)	2946.67 (2075.51, 4122.06)	0.14 (0.10, 0.18)	15284 (10150, 21286)	270.99 (179.95, 377.6)	13016 (7885, 19596)	351.84 (213.1, 535.04)	0.13 (0.02, 0.24)
**Germany**	608775 (504630, 727604)	819.78 (682.15, 996.71)	764048 (555526, 1018039)	1075.74 (782.5, 1456.78)	0.43 (0.26, 0.60)	5195921 (4526587, 5929948)	6029.06 (5246.21, 6942.35)	6701716 (4856966, 8894927)	7595.91 (5541.43, 10217.32)	0.35 (0.25, 0.45)	611115 (422786, 826817)	717.42 (502.71, 973.05)	781497 (483709, 1138913)	904.8 (554.66, 1348.06)	0.34 (0.24, 0.44)
**Ghana**	72185 (57598, 90603)	460.15 (371.74, 574.13)	185987 (131137, 255251)	524.33 (374.85, 707.24)	0.13 (0.03, 0.23)	403296 (317744, 516312)	2849.16 (2298.59, 3503.56)	1068227 (776282, 1485626)	3221.76 (2358.11, 4400.3)	0.18 (0.14, 0.22)	48478 (31902, 70442)	338.3 (229.08, 474.11)	128671 (76784, 190336)	384.3 (228.76, 558.44)	0.19 (0.06, 0.32)
**Greece**	81180 (66551, 101418)	803.34 (662.94, 1006.92)	89093 (64452, 120227)	1060.53 (754.12, 1441.67)	0.24 (0.07, 0.41)	627127 (515339, 759546)	5593.37 (4572.22, 6894.1)	740317 (540741, 980974)	7208.74 (5208.68, 9505.15)	0.26 (0.20, 0.32)	74255 (50775, 102852)	667.65 (450.48, 924.15)	86639 (55535, 128054)	860.23 (541.3, 1315.72)	0.27 (0.21, 0.33)
**Greenland**	402 (316, 515)	678.53 (538.75, 864.24)	487 (337, 664)	883.16 (617.79, 1211.79)	0.29 (-0.30, 0.89)	2748 (2165, 3518)	4679.11 (3752.09, 5826.59)	3562 (2492, 4871)	5970.8 (4098.36, 8197.65)	0.21 (-0.11, 0.52)	325 (211, 461)	550.23 (362.85, 776.29)	419 (251, 639)	705.97 (425.52, 1085.92)	0.23 (-0.41, 0.87)
**Grenada**	550 (447, 704)	637.07 (519.56, 802.91)	829 (594, 1162)	792.66 (568.63, 1109.48)	0.22 (-0.27, 0.72)	3447 (2799, 4281)	4264.32 (3484.2, 5192.27)	5663 (4023, 7605)	5177.31 (3673.18, 6956.35)	0.22 (0, 0.45)	411 (273, 578)	506.28 (339.23, 704.5)	670 (413, 1011)	614.06 (374.54, 934.67)	0.23 (-0.34, 0.81)
**Guam**	811 (655, 1044)	564.83 (462.05, 708.34)	1084 (771, 1461)	697.4 (492.85, 943.06)	0.16 (-0.31, 0.64)	5059 (4071, 6349)	3658.07 (2972.92, 4488.22)	7339 (5336, 9677)	4454.13 (3195.01, 5894.98)	0.22 (0.04, 0.40)	612 (407, 873)	438.84 (294.68, 612.99)	874 (536, 1317)	534.19 (330.16, 810.33)	0.23 (-0.22, 0.68)
**Guatemala**	53642 (43092, 68617)	615.34 (502.83, 780.49)	129238 (92803, 179686)	780.27 (563.94, 1079.72)	0.28 (0.15, 0.40)	306070 (241024, 385831)	4055.51 (3298.85, 5022.24)	808896 (576084, 1079885)	5077.05 (3676.29, 6760.28)	0.32 (0.23, 0.40)	36506 (24498, 51503)	476.97 (321.48, 658.99)	96308 (58789, 144900)	600.05 (374.15, 886.97)	0.29 (0.13, 0.46)
**Guinea**	30146 (24259, 38120)	481.42 (389.86, 600.36)	76664 (53429, 105487)	557.28 (391.34, 764.9)	0.15 (0.02, 0.28)	170119 (135548, 215045)	3029.9 (2438.2, 3752.31)	427424 (296260, 600138)	3471.17 (2486.18, 4787.92)	0.14 (0.09, 0.19)	20367 (13235, 29281)	359.25 (240.42, 508.99)	51430 (30968, 79870)	412.51 (253.33, 629.1)	0.14 (-0.01, 0.3)
**Guinea-Bissau**	4897 (3917, 6188)	467.97 (380.73, 593.52)	11429 (7932, 16033)	535.13 (384.73, 729.52)	0.15 (-0.14, 0.45)	26825 (20814, 34278)	2909.65 (2378.09, 3566.23)	63511 (44314, 89169)	3299.51 (2368.47, 4590.25)	0.13 (-0.07, 0.33)	3209 (2094, 4666)	343.43 (229.66, 477)	7642 (4675, 11898)	391.49 (246.46, 605.4)	0.17 (-0.10, 0.44)
**Guyana**	5052 (4026, 6508)	645.67 (523.89, 817.41)	6736 (4769, 9503)	860.44 (607.03, 1210.34)	0.24 (-0.01, 0.50)	31177 (25002, 39245)	4323.64 (3516.07, 5245.55)	43786 (30946, 59945)	5624.38 (4006.18, 7687.78)	0.23 (0.11, 0.36)	3707 (2458, 5210)	507.87 (346.29, 697.42)	5166 (3115, 7780)	661.72 (402.61, 997.52)	0.23 (-0.03, 0.49)
**Haiti**	42931 (34932, 55302)	653.08 (529.59, 831.17)	104738 (73709, 146591)	787.02 (558.79, 1092.12)	0.20 (0.06, 0.34)	259384 (209148, 325656)	4392.93 (3584.15, 5423.45)	658379 (465085, 912860)	5246.59 (3771.15, 7227.08)	0.21 (0.16, 0.27)	30673 (20851, 42896)	514.16 (348.99, 700.14)	77705 (47232, 117217)	614.48 (372.81, 920.75)	0.22 (0.04, 0.39)
**Honduras**	29867 (24011, 37869)	603.95 (491.47, 763.63)	88448 (60805, 124042)	830.46 (572.11, 1173.6)	0.33 (0.20, 0.47)	171262 (135570, 217526)	3952.34 (3209.72, 4876.05)	543929 (388164, 750300)	5332.85 (3815.41, 7407.98)	0.36 (0.30, 0.43)	20624 (13508, 29207)	469.78 (315.37, 652.9)	65251 (39226, 98631)	634.49 (387.86, 952.92)	0.38 (0.21, 0.55)

Characteristics	Incidence					Prevalence					YLDs				
	1992		2021		1992–2021	1992		2021		1992–2021	1992		2021		1992–2021
	Number No. (95% UI)	ASR per 100,000 No. (95% UI)	Incident cases No. (95% UI)	ASR per 100,000 No. (95% UI)	Net drift (% per year)	Number No. (95% UI)	ASR per 100,000 No. (95% UI)	Number No. (95% UI)	ASR per 100,000 No. (95% UI)	Net drift (% per year)	Number No. (95% UI)	ASR per 100,000 No. (95% UI)	Number No. (95% UI)	ASR per 100,000 No. (95% UI)	Net drift (% per year)
**Hungary**	53009 (43416, 65626)	504.45 (414.52, 633.9)	59348 (43073, 81213)	661.57 (474.16, 916.07)	0.18 (0.04, 0.31)	383457 (314352, 466239)	3373.53 (2768.59, 4104.56)	447550 (330454, 608457)	4264.27 (3110.13, 5793.35)	0.15 (0.11, 0.18)	44882 (30669, 61973)	398.62 (270.55, 549.03)	52206 (33110, 79528)	507.85 (318.41, 766.24)	0.18 (0.12, 0.25)
**Iceland**	1977 (1616, 2503)	773.46 (632.1, 971.74)	2758 (2013, 3661)	854.56 (612.1, 1155.71)	0.11 (-0.21, 0.43)	14015 (11465, 17142)	5276.68 (4324.2, 6495.17)	20703 (14989, 27208)	5749.34 (4078.77, 7571.57)	0.09 (-0.01, 0.19)	1672 (1122, 2323)	630.79 (421.46, 876.28)	2451 (1505, 3663)	687.51 (412.66, 1026.43)	0.09 (-0.21, 0.39)
**India**	4044005 (3338051, 4967943)	474.34 (395.82, 572.22)	8625554 (7051002, 10467536)	579.94 (480.12, 702.24)	0.28 (-0.56, 1.13)	23242319 (19891561, 27138645)	2950.8 (2529.49, 3411.72)	51980967 (44454000, 60381426)	3545.22 (3042.64, 4102.3)	0.30 (-0.07, 0.67)	2737973 (1895492, 3726539)	343.57 (237.1, 465.28)	6117755 (4251987, 8281114)	415.41 (287.24, 561.78)	0.33 (-0.08, 0.73)
**Indonesia**	1016824 (840924, 1241517)	528.62 (441.43, 635.49)	1986926 (1633184, 2412671)	683.75 (562.15, 822.04)	0.22 (0.12, 0.32)	5962410 (5138639, 6907003)	3341.65 (2895.95, 3836.97)	12389897 (10585884, 14627323)	4227.47 (3644.82, 4971.76)	0.24 (0.19, 0.28)	718447 (492143, 986955)	397.95 (273.59, 541.9)	1486922 (1017632, 2061944)	506.06 (347.16, 698.75)	0.25 (0.20, 0.29)
**Iran (Islamic Republic of)**	596816 (490718, 733985)	921.74 (762.8, 1122.34)	986389 (808525, 1216359)	1142.02 (938.58, 1401.35)	0.33 (-0.15, 0.81)	3816876 (3271524, 4431606)	6746.03 (5873.75, 7700.81)	7288282 (6217244, 8378425)	8198.38 (7057.54, 9426.24)	0.41 (0.20, 0.62)	458859 (317350, 639341)	800.05 (552.95, 1090.39)	864198 (587427, 1191910)	973.7 (659.07, 1345.76)	0.40 (0.18, 0.63)
**Iraq**	142460 (114137, 179638)	713.3 (585.28, 901.52)	373399 (263928, 520561)	846.84 (608.51, 1170.4)	0.26 (-0.18, 0.71)	883828 (692456, 1118317)	5000.48 (4039.02, 6147.19)	2460206 (1733541, 3379014)	5837.95 (4136.59, 7886.99)	0.28 (0.09, 0.47)	106016 (69291, 152220)	591.25 (399.08, 823.82)	293838 (177147, 444955)	690.9 (425.02, 1028.28)	0.27 (0.06, 0.47)
**Ireland**	32323 (26325, 40908)	882.7 (718.01, 1119.33)	50522 (36304, 69556)	1110.21 (782.58, 1536.01)	0.30 (0.12, 0.48)	240228 (195183, 295869)	6432.11 (5213.02, 7945.86)	397566 (295313, 539581)	7883.66 (5838.81, 10817.19)	0.33 (0.19, 0.47)	28587 (19232, 39691)	766.33 (514.83, 1064.34)	46939 (29581, 73014)	939.19 (594.21, 1434.14)	0.34 (0.18, 0.49)
**Israel**	32556 (26454, 41672)	612.55 (500.39, 786.53)	67684 (48477, 93202)	725.47 (518.5, 1000.3)	0.47 (0.16, 0.78)	197937 (160348, 241356)	3773.14 (3065.62, 4614.56)	422180 (304559, 576466)	4423.65 (3184.99, 6006.05)	0.69 (0.52, 0.85)	23749 (15898, 33622)	452.13 (302.28, 639.63)	50367 (30571, 75570)	529.92 (320.59, 800.77)	0.69 (0.51, 0.87)
**Italy**	432221 (357589, 523717)	805.67 (669.31, 977.86)	507150 (416874, 611940)	1016.76 (834.28, 1240.31)	0.44 (0.23, 0.64)	3428389 (2998047, 3902535)	5608.02 (4860.26, 6406.5)	4156262 (3547724, 4759163)	6814.75 (5788.4, 7875.01)	0.44 (0.34, 0.53)	403417 (279077, 544995)	667.04 (463.83, 911.23)	485995 (339265, 660564)	813.96 (553.81, 1127.37)	0.46 (0.36, 0.56)
**Jamaica**	15007 (12115, 19322)	621.96 (507.87, 785.17)	23220 (16405, 31635)	804.95 (568.48, 1083.92)	0.19 (-0.01, 0.39)	93609 (75777, 115804)	4098.26 (3362.54, 5037.24)	156468 (110890, 213858)	5211.03 (3695.24, 7072.5)	0.26 (0.21, 0.30)	11228 (7498, 15608)	488.74 (331.73, 674.58)	18652 (11078, 27898)	622.06 (371.53, 924.98)	0.25 (0.12, 0.38)
**Japan**	557577 (468806, 664263)	443.39 (370.06, 533.88)	581065 (490019, 690644)	535.01 (446.8, 638.38)	-0.24 (-0.37, -0.10)	3632319 (3144603, 4180810)	2684.06 (2312.83, 3094.33)	4057951 (3504342, 4662153)	3190.69 (2708.27, 3733.17)	-0.35 (-0.42, -0.28)	433989 (297579, 592910)	322.94 (222.53, 443.86)	478475 (335325, 645151)	384.89 (263.37, 521.37)	-0.34 (-0.41, -0.27)
**Jordan**	30589 (24392, 39177)	716.29 (582.65, 909.58)	112466 (80016, 156130)	852.48 (609.34, 1182.44)	0.18 (0.01, 0.35)	187809 (147774, 240089)	4897.32 (3982.54, 6013.59)	737436 (513268, 978697)	5694.68 (4016.1, 7541.96)	0.15 (0.08, 0.21)	22743 (14961, 32773)	584.13 (397.52, 821.39)	88575 (52135, 137122)	679.18 (404.09, 1050.26)	0.13 (-0.08, 0.33)
**Kazakhstan**	55796 (45272, 70142)	341.94 (278.88, 428.51)	78895 (56091, 108257)	415.75 (297.51, 565.93)	0.16 (0, 0.31)	326554 (263898, 406516)	2061.81 (1674.67, 2530.99)	465618 (329076, 607044)	2462.93 (1743.73, 3204.34)	0.12 (0.09, 0.16)	38896 (26432, 54609)	244.34 (167.32, 340.48)	55287 (35457, 80812)	292.54 (187.19, 431.56)	0.13 (0.04, 0.21)
**Kenya**	125518 (101557, 152675)	534.62 (445.32, 643.66)	329381 (266984, 402101)	628.26 (520.87, 758.52)	0.14 (-0.02, 0.30)	686190 (588976, 811430)	3376.65 (2915.67, 3912.65)	1886876 (1604317, 2232091)	3939.01 (3390.74, 4585.48)	0.27 (0.22, 0.31)	82483 (56264, 113933)	399.52 (277.26, 541.74)	226600 (153933, 312499)	467.2 (319.98, 634.63)	0.30 (0.20, 0.40)
**Kiribati**	435 (352, 551)	569.44 (466.59, 715.67)	835 (594, 1144)	666.22 (478.01, 908.99)	0.11 (-0.52, 0.74)	2584 (2075, 3255)	3744.38 (3036.22, 4562.07)	5058 (3645, 7141)	4323.58 (3140.3, 5957.35)	0.13 (-0.17, 0.43)	309 (203, 440)	441.65 (298.8, 612.03)	605 (369, 884)	511.7 (318.52, 746.76)	0.22 (-0.40, 0.84)
**Kuwait**	12382 (10003, 15775)	689.05 (566.06, 866.64)	36403 (26141, 50368)	761.38 (556.43, 1055.96)	0.25 (0.01, 0.49)	78690 (62267, 99343)	4508.78 (3685.39, 5505.12)	256068 (181744, 346024)	5065.57 (3632.52, 6817.41)	0.28 (0.20, 0.36)	9511 (6173, 13798)	540.5 (359.86, 767.58)	30497 (18804, 46382)	604.06 (377.49, 926.51)	0.23 (-0.02, 0.48)

Characteristics	Incidence					Prevalence					YLDs				
	1992		2021		1992–2021	1992		2021		1992–2021	1992		2021		1992–2021
	Number No. (95% UI)	ASR per 100,000 No. (95% UI)	Incident cases No. (95% UI)	ASR per 100,000 No. (95% UI)	Net drift (% per year)	Number No. (95% UI)	ASR per 100,000 No. (95% UI)	Number No. (95% UI)	ASR per 100,000 No. (95% UI)	Net drift (% per year)	Number No. (95% UI)	ASR per 100,000 No. (95% UI)	Number No. (95% UI)	ASR per 100,000 No. (95% UI)	Net drift (% per year)
**Kyrgyzstan**	15234 (12288, 19463)	337.6 (276.14, 422)	30197 (20687, 42342)	434.5 (298.67, 606.64)	0.24 (0.10, 0.38)	85845 (68959, 108669)	2026.09 (1639.46, 2505.91)	170311 (118530, 234580)	2549.35 (1793, 3521.18)	0.20 (0.14, 0.25)	10285 (6823, 14606)	240.68 (161.12, 337.15)	20453 (12191, 31000)	304.52 (183.24, 460.62)	0.20 (0.04, 0.37)
**Lao People's Democratic Republic**	26377 (21110, 34205)	624.24 (510.19, 791.01)	56839 (41106, 79399)	750.43 (541.82, 1044.47)	0.20 (0.02, 0.37)	158657 (126810, 197589)	4277.41 (3477.92, 5231.87)	366393 (258142, 500588)	5075.8 (3631.09, 6931.4)	0.23 (0.17, 0.30)	19052 (12494, 26609)	507.26 (335.6, 692.32)	44098 (27437, 64325)	606.03 (378.48, 872.9)	0.23 (0.05, 0.41)
**Latvia**	14127 (11585, 17532)	542.17 (441.62, 682.11)	13469 (9529, 18490)	809.38 (569.34, 1129.96)	0.26 (0.14, 0.38)	102109 (84004, 125256)	3654.37 (2973.47, 4481.51)	101052 (72449, 139298)	5201.7 (3603.25, 7191.51)	0.21 (0.17, 0.26)	12000 (8181, 16498)	433.52 (292.81, 596.81)	11781 (7388, 17305)	619.91 (387.19, 906.84)	0.24 (0.11, 0.36)
**Lebanon**	25894 (21339, 32632)	818.12 (671.33, 1036.1)	63879 (45411, 85516)	1168.05 (824.02, 1570.7)	0.35 (0.15, 0.55)	183734 (147578, 228818)	6088.11 (4905.75, 7493.25)	473053 (331020, 634186)	8274.66 (5766.56, 11065.29)	0.32 (0.15, 0.50)	21870 (14583, 31018)	720.54 (485.14, 1011.47)	55917 (33924, 82475)	981.03 (592.83, 1442.83)	0.32 (0.13, 0.52)
**Lesotho**	8425 (6809, 10678)	542.38 (436.42, 685.99)	15359 (10671, 21013)	768.09 (539.64, 1044.65)	0.24 (0.05, 0.42)	49103 (39260, 61016)	3530.55 (2858.23, 4342.19)	90820 (66125, 126008)	4822.31 (3521.82, 6498.92)	0.25 (0.16, 0.34)	5867 (3852, 8229)	417.33 (277.29, 576.33)	10720 (6809, 16032)	562.68 (364.68, 852.61)	0.20 (0, 0.40)
**Liberia**	9697 (7765, 12223)	488.24 (398.63, 616.64)	30528 (21867, 41651)	534.46 (386.87, 729.05)	0.12 (-0.11, 0.35)	54300 (42830, 69490)	3079.42 (2465.24, 3799.77)	176198 (124281, 242145)	3356.49 (2410.6, 4497.28)	0.15 (0.05, 0.25)	6377 (4225, 9055)	357.74 (240.84, 492.26)	20936 (12542, 33166)	393.61 (237.98, 607.59)	0.18 (-0.01, 0.38)
**Libya**	34066 (27268, 43005)	734.86 (601.41, 937.08)	65019 (45650, 89924)	910.93 (645.24, 1275.26)	0.21 (-0.01, 0.42)	212033 (166901, 270490)	5060.39 (4105.67, 6220.31)	458304 (328436, 626545)	6188.3 (4447.95, 8519.96)	0.25 (0.20, 0.29)	25628 (16831, 36886)	603.91 (404.38, 837.93)	54530 (33340, 80996)	735.5 (441.92, 1099.25)	0.21 (0.08, 0.34)
**Lithuania**	21816 (17849, 27484)	593.49 (480.73, 750.87)	21148 (15293, 28793)	871.86 (635.86, 1196.9)	0.20 (0.03, 0.37)	163009 (133490, 199588)	4194.5 (3426.03, 5139.24)	168600 (121806, 225456)	5865.14 (4304.22, 7983.86)	0.21 (0.14, 0.27)	19231 (12939, 26471)	497.79 (333.79, 688.81)	19649 (12409, 29340)	698.41 (428.37, 1061.48)	0.22 (0.12, 0.32)
**Luxembourg**	2844 (2320, 3588)	785.88 (639.88, 988.25)	5454 (3841, 7470)	944.79 (664.6, 1299.27)	0.21 (-0.05, 0.46)	22047 (18163, 26783)	5422.04 (4455.93, 6642.58)	42079 (30714, 57003)	6335.6 (4545.93, 8549.48)	0.11 (0.03, 0.19)	2602 (1760, 3570)	645.9 (430.15, 891.75)	4976 (3234, 7413)	758.27 (485.2, 1141.64)	0.10 (-0.14, 0.34)
**Madagascar**	66851 (53288, 87191)	552.75 (447.07, 708.7)	194728 (133547, 278453)	662.35 (463.38, 947.8)	0.22 (0.11, 0.33)	383559 (303278, 493584)	3614.48 (2919.04, 4510.12)	1130782 (779477, 1586289)	4304.25 (3032.07, 6023.64)	0.28 (0.23, 0.33)	45784 (29942, 64012)	426.52 (285.16, 594.78)	135927 (80041, 204894)	511.34 (300.64, 761.38)	0.31 (0.17, 0.45)
**Malawi**	54624 (43313, 71172)	545.15 (440.88, 695.37)	136881 (94419, 191824)	675.33 (480.41, 950.56)	0.22 (0.08, 0.36)	312212 (246351, 398963)	3552.37 (2887.49, 4428.84)	780958 (543722, 1086434)	4364.58 (3074.75, 5960.74)	0.31 (0.25, 0.37)	36990 (23972, 52687)	415.81 (276.55, 571.3)	93824 (54581, 140183)	517.08 (307.76, 759.12)	0.37 (0.19, 0.55)
**Malaysia**	116633 (93182, 149005)	618.68 (506.42, 782.04)	257688 (182572, 365326)	781.08 (553.68, 1095.84)	0.18 (-0.22, 0.58)	729533 (591771, 909578)	4238.2 (3466.09, 5212.29)	1738286 (1237189, 2389048)	5217.07 (3748.36, 7171.15)	0.14 (-0.06, 0.34)	87827 (56967, 123778)	504.3 (336.11, 702.98)	207794 (127809, 314535)	622.15 (379.8, 939.12)	0.14 (-0.08, 0.36)
**Maldives**	1206 (954, 1538)	538.86 (442.62, 682.85)	3451 (2431, 4853)	625.3 (449.94, 861.2)	0.07 (-0.29, 0.44)	6659 (5300, 8401)	3418.35 (2777.13, 4177.46)	21567 (15416, 29663)	3885.69 (2826.42, 5366.25)	0.14 (-0.06, 0.34)	801 (530, 1148)	405.66 (276.56, 561.07)	2591 (1563, 3979)	464.52 (283.6, 704.32)	0.08 (-0.33, 0.49)
**Mali**	38368 (30807, 48287)	441.07 (360.69, 557.9)	120206 (84604, 167234)	497.6 (350.16, 688.08)	0.17 (0.02, 0.31)	207623 (164738, 262284)	2678.23 (2160.39, 3294.8)	645653 (448745, 902549)	3017.21 (2125.27, 4271.21)	0.17 (0.11, 0.23)	24792 (16095, 35513)	316.21 (209.8, 438.22)	77606 (45928, 124401)	357.57 (218.62, 558.75)	0.19 (0, 0.37)
**Malta**	3069 (2518, 3808)	830.64 (676.45, 1022.41)	3618 (2574, 4980)	972.58 (683.29, 1361.75)	0.17 (-0.10, 0.43)	23035 (18944, 27893)	5907.93 (4844.6, 7266.11)	30395 (21364, 41146)	6741.54 (4683.23, 9201.83)	0.11 (0.03, 0.20)	2744 (1850, 3827)	706.39 (472.45, 997.87)	3570 (2254, 5371)	806.3 (503.16, 1241.65)	0.09 (-0.16, 0.34)
**Marshall Islands**	275 (219, 347)	568.11 (462.17, 719.44)	384 (274, 527)	654.76 (469.34, 895.79)	0.21 (-0.59, 1.02)	1524 (1204, 1923)	3696.53 (3032.09, 4511.5)	2363 (1730, 3285)	4200.81 (3075.28, 5785.94)	0.10 (-0.25, 0.45)	185 (120, 266)	439.39 (292.99, 606.9)	283 (176, 437)	498.19 (314.99, 758.79)	0.12 (-0.72, 0.96)

Characteristics	Incidence					Prevalence					YLDs				
	1992		2021		1992–2021	1992		2021		1992–2021	1992		2021		1992–2021
	Number No. (95% UI)	ASR per 100,000 No. (95% UI)	Incident cases No. (95% UI)	ASR per 100,000 No. (95% UI)	Net drift (% per year)	Number No. (95% UI)	ASR per 100,000 No. (95% UI)	Number No. (95% UI)	ASR per 100,000 No. (95% UI)	Net drift (% per year)	Number No. (95% UI)	ASR per 100,000 No. (95% UI)	Number No. (95% UI)	ASR per 100,000 No. (95% UI)	Net drift (% per year)
**Mauritania**	9513 (7556, 11973)	446.4 (360.85, 557.24)	22696 (15880, 32694)	501.69 (360.61, 700.3)	0.12 (-0.11, 0.36)	52611 (41648, 67497)	2731.76 (2210.37, 3378.09)	125759 (89956, 178314)	3037.69 (2219.09, 4218.33)	0.08 (-0.02, 0.19)	6334 (4102, 9203)	325.35 (216.83, 455.17)	15201 (9148, 22947)	363.3 (219.83, 553.61)	0.12 (-0.08, 0.32)
**Mauritius**	6309 (5051, 8096)	544.29 (438.86, 688.62)	8650 (6048, 11893)	676.52 (463.08, 935.3)	0.18 (-0.05, 0.41)	39476 (32054, 49158)	3542.55 (2898.93, 4305.13)	60001 (42245, 79813)	4271.61 (3044.6, 5714.27)	0.12 (0.05, 0.20)	4718 (3133, 6595)	420 (282.96, 578.12)	7048 (4240, 10353)	506.61 (304.4, 740.09)	0.10 (-0.13, 0.33)
**Mexico**	416810 (344778, 500380)	472.66 (397.5, 562.16)	972865 (801407, 1177529)	731.44 (603.91, 882.6)	0.64 (0.22, 1.05)	2370921 (2040023, 2749098)	2904.87 (2519.88, 3320.84)	6101986 (5153074, 7113373)	4519.86 (3821.22, 5276.48)	0.83 (0.64, 1.01)	284705 (195945, 391209)	343.95 (240.29, 466.74)	725327 (486257, 988346)	537.35 (361.5, 731.13)	0.83 (0.62, 1.03)
**Micronesia (Federated States of)**	616 (495, 767)	568.49 (466.4, 707.9)	703 (496, 975)	656.38 (464.78, 906.28)	0.14 (-0.45, 0.73)	3529 (2797, 4444)	3703.63 (3027.06, 4476.49)	4387 (3103, 6134)	4226.43 (3038.43, 5867.05)	0.17 (-0.10, 0.44)	426 (280, 607)	440.46 (297.56, 605.51)	526 (332, 780)	502.5 (315.98, 748.23)	0.04 (-0.59, 0.68)
**Monaco**	208 (171, 253)	790.85 (648.46, 995.51)	303 (217, 415)	1002.39 (702.08, 1366.97)	0.26 (-0.40, 0.93)	1798 (1487, 2173)	5486.91 (4518.27, 6688.27)	2543 (1782, 3502)	6740.28 (4814.03, 9267.29)	0.18 (-0.06, 0.42)	211 (144, 292)	656.68 (444.52, 914.53)	298 (189, 446)	805.59 (493.32, 1213.17)	0.19 (-0.44, 0.84)
**Mongolia**	7499 (5974, 9506)	341.04 (278.24, 428.04)	12663 (9081, 17382)	377.89 (271.89, 517.95)	-0.06 (-0.31, 0.18)	41015 (32795, 51781)	2044.88 (1661.57, 2510.73)	72675 (52957, 102268)	2256.89 (1636.92, 3146.73)	-0.10 (-0.23, 0.03)	4934 (3182, 7149)	242.82 (162.8, 338.58)	8671 (5390, 12675)	268.06 (167.42, 389.76)	-0.10 (-0.32, 0.11)
**Montenegro**	3316 (2682, 4157)	515.08 (418.07, 649.04)	4401 (3109, 5946)	736.06 (513.51, 1006.58)	0.31 (0.04, 0.58)	22622 (18447, 27589)	3462.9 (2836.39, 4232.16)	31540 (22207, 42285)	4757.56 (3370.41, 6358.2)	0.25 (0.16, 0.33)	2691 (1814, 3681)	412.13 (277.39, 565.98)	3710 (2286, 5485)	566.48 (351.27, 843.72)	0.23 (-0.03, 0.50)
**Morocco**	194782 (157570, 251479)	710.55 (579.64, 900.33)	336701 (246909, 452168)	894.56 (658.93, 1198.88)	0.26 (0.09, 0.43)	1242343 (982222, 1547062)	4852.22 (3932.45, 5957.42)	2282348 (1643839, 3088910)	5985.12 (4316.49, 8092.99)	0.26 (0.21, 0.31)	149134 (99845, 212172)	576.83 (390.37, 798.58)	270397 (168855, 405942)	708.94 (442.74, 1062.73)	0.23 (0.18, 0.29)
**Mozambique**	72378 (58294, 93602)	560.29 (454.87, 721)	214401 (152727, 300602)	685.68 (482.7, 959.39)	0.17 (0.07, 0.28)	420032 (334367, 528179)	3698.32 (2975.78, 4571.16)	1209450 (862029, 1658791)	4475.38 (3181.54, 5983.42)	0.25 (0.20, 0.30)	49594 (32300, 69545)	431.64 (288.53, 594.07)	143451 (88126, 214883)	522.58 (323.31, 781.18)	0.26 (0.13, 0.39)
**Myanmar**	229248 (183757, 296879)	546.3 (444.47, 687.08)	421127 (299187, 564055)	729 (520.38, 973.8)	0.23 (0.08, 0.38)	1379896 (1117604, 1719563)	3553.95 (2888.44, 4350.68)	2655200 (1905358, 3523063)	4629.71 (3317.33, 6125.07)	0.27 (0.22, 0.31)	165226 (109162, 231533)	420.99 (285.55, 579)	317607 (184956, 477018)	552.12 (323.21, 827.14)	0.28 (0.23, 0.34)
**Namibia**	7906 (6366, 10184)	533.38 (435.43, 680.43)	19811 (13765, 27179)	771.32 (536.08, 1059.04)	0.29 (0.08, 0.49)	45464 (36252, 57550)	3420.3 (2781.8, 4213.69)	117152 (82743, 158489)	4827.4 (3407.32, 6552.25)	0.33 (0.20, 0.46)	5455 (3589, 7736)	405.36 (273.23, 561.79)	13968 (9057, 20843)	570.49 (368.59, 858.19)	0.34 (0.09, 0.58)
**Nauru**	59 (48, 74)	568.24 (466.12, 712.98)	75 (54, 103)	656.76 (472.7, 887.14)	0.16 (-1.25, 1.59)	341 (271, 429)	3663.99 (2956.91, 4494.33)	448 (324, 622)	4254.23 (3081.65, 5812.7)	0.18 (-0.46, 0.83)	41 (27, 59)	436.32 (292.97, 605.28)	54 (32, 84)	505.72 (303.08, 765.57)	0.03 (-1.60, 1.68)
**Nepal**	89400 (72075, 115552)	479.44 (385.98, 610.64)	203366 (141352, 283054)	636.54 (444.88, 870.63)	0.38 (0.22, 0.53)	505812 (406053, 638186)	3021.61 (2470.51, 3750.39)	1215289 (850020, 1733447)	3945.11 (2772.74, 5527.81)	0.46 (0.40, 0.52)	59827 (40065, 84148)	352.73 (238.58, 488.4)	143804 (86635, 226590)	463.71 (282.82, 726.91)	0.49 (0.39, 0.59)
**Netherlands**	118752 (99877, 145304)	830.51 (694.79, 1011.96)	155475 (113724, 210728)	1058.9 (747.87, 1445.1)	0.20 (0.02, 0.38)	1051798 (947883, 1170619)	6546.93 (5848.54, 7338.94)	1390218 (993673, 1841237)	7885.11 (5633.28, 10590.76)	-0.08 (-0.19, 0.03)	125078 (86449, 168086)	783.95 (542.26, 1057.27)	163657 (101629, 239548)	942.86 (579.36, 1387.18)	-0.10 (-0.21, 0.02)
**New Zealand**	33866 (27729, 42458)	973.8 (796.38, 1215.09)	48517 (37316, 62883)	1001.21 (774.64, 1301.56)	0.03 (-0.25, 0.31)	264909 (228379, 308819)	7296.11 (6322.21, 8490.1)	398263 (314031, 497843)	7421.44 (5871.23, 9291.85)	0 (-0.07, 0.06)	31235 (21476, 42729)	862.42 (594.8, 1181.61)	46910 (30020, 67326)	880.94 (556.48, 1268.55)	0.01 (-0.08, 0.11)
**Nicaragua**	25755 (20632, 33004)	621.82 (508.26, 793.05)	54456 (39071, 75513)	783.85 (561.06, 1083.74)	0.27 (0.12, 0.41)	149382 (119160, 189310)	4140.15 (3379.47, 5046.14)	347006 (248986, 473155)	5121.32 (3709.56, 6981.76)	0.28 (0.23, 0.33)	17948 (11671, 25298)	490.51 (328.52, 673.14)	41483 (25608, 62719)	608.93 (381.52, 917.32)	0.28 (0.14, 0.43)

Characteristics	Incidence					Prevalence					YLDs				
	1992		2021		1992–2021	1992		2021		1992–2021	1992		2021		1992–2021
	Number No. (95% UI)	ASR per 100,000 No. (95% UI)	Incident cases No. (95% UI)	ASR per 100,000 No. (95% UI)	Net drift (% per year)	Number No. (95% UI)	ASR per 100,000 No. (95% UI)	Number No. (95% UI)	ASR per 100,000 No. (95% UI)	Net drift (% per year)	Number No. (95% UI)	ASR per 100,000 No. (95% UI)	Number No. (95% UI)	ASR per 100,000 No. (95% UI)	Net drift (% per year)
**Niger**	37519 (29809, 47918)	452.69 (366.4, 569)	117462 (81401, 163819)	475.06 (341.27, 653.29)	0.16 (-0.01, 0.34)	201109 (158735, 253500)	2775.33 (2216.06, 3417.63)	627191 (436366, 880553)	2930.04 (2104.31, 3971.81)	0.16 (0.09, 0.23)	24142 (15767, 35331)	328.59 (220.64, 467.89)	76145 (44181, 118559)	350.1 (204.58, 540.71)	0.16 (-0.05, 0.37)
**Nigeria**	437684 (361342, 526127)	467.15 (388.68, 565.45)	1157439 (943853, 1403882)	494.94 (411.29, 602.3)	0.24 (-0.50, 0.98)	2477161 (2118764, 2884769)	2896.37 (2503.92, 3347.93)	6478651 (5474811, 7610167)	3075.76 (2657.58, 3560.82)	0.23 (-0.06, 0.52)	295020 (203709, 407286)	341.16 (235.25, 466.77)	778976 (525922, 1080426)	364.95 (250.47, 501.13)	0.27 (-0.05, 0.58)
**Niue**	13 (11, 16)	567.13 (467.1, 713.1)	11 (8, 15)	658.35 (464.28, 924.91)	0.74 (-2.22, 3.78)	82 (67, 99)	3712.71 (3044.45, 4494.17)	74 (53, 100)	4242.66 (3038.6, 5758.69)	0.03 (-1.16, 1.24)	10 (7, 14)	443.17 (296.59, 612.52)	9 (5, 13)	504.61 (306.32, 754.54)	1.27 (-2.06, 4.71)
**North Macedonia**	10481 (8524, 13225)	512.28 (416.44, 647.55)	16170 (11698, 21595)	763.06 (551.23, 1037.29)	0.34 (0.22, 0.46)	70557 (57245, 86314)	3425.5 (2778.64, 4177.31)	115303 (83531, 154401)	4868.05 (3554.51, 6511.97)	0.31 (0.24, 0.38)	8386 (5644, 11484)	406.8 (274.44, 556.59)	13560 (8190, 20010)	579.64 (347.75, 853.72)	0.32 (0.19, 0.46)
**Northern Mariana Islands**	277 (221, 357)	569.36 (462.61, 713.75)	352 (252, 483)	713.25 (510.09, 988.45)	0.12 (-0.60, 0.84)	1725 (1372, 2196)	3656.97 (2975.29, 4479.92)	2295 (1647, 3111)	4517.49 (3277.46, 6274.73)	0.25 (-0.09, 0.59)	209 (134, 293)	438.54 (291.37, 611.47)	274 (173, 407)	541.15 (337.56, 804.67)	0.20 (-0.55, 0.95)
**Norway**	38798 (31863, 47475)	969.01 (798.62, 1175.84)	52512 (43323, 63787)	1067.02 (876.49, 1299.59)	0.05 (-0.05, 0.16)	322267 (282441, 367073)	7173.44 (6226.99, 8193.12)	425866 (361240, 499501)	7544.74 (6364.53, 8928.18)	-0.08 (-0.11, -0.04)	38011 (26384, 51401)	854.62 (593.31, 1175.91)	50243 (35128, 67803)	901.75 (621.43, 1246.91)	-0.05 (-0.11, 0.01)
**Oman**	15420 (12323, 19423)	706.33 (572.62, 885.92)	42559 (30713, 57926)	869.88 (624.08, 1158.12)	0.19 (-0.07, 0.45)	90847 (71483, 114580)	4668.73 (3781.52, 5696.4)	271740 (189928, 370977)	5624.34 (3947.31, 7639.36)	0.20 (0.11, 0.29)	10999 (7262, 15780)	557.52 (380.63, 777.21)	32703 (19847, 48698)	673.09 (412.19, 1001.77)	0.16 (-0.10, 0.41)
**Pakistan**	536255 (444034, 650807)	512.71 (425.4, 616.08)	1408226 (1098567, 1783412)	604.56 (470.69, 766.9)	0.24 (0.13, 0.34)	3013340 (2592918, 3518708)	3215.37 (2775.98, 3709.27)	8061159 (6508201, 9993869)	3739.62 (3035.47, 4651)	0.26 (0.22, 0.30)	358285 (246803, 488137)	377.89 (259.29, 512.69)	957510 (643971, 1362632)	439.19 (301.44, 622.61)	0.25 (0.21, 0.30)
**Palau**	94 (76, 121)	568.99 (464.58, 719.07)	117 (83, 158)	642.65 (455.39, 870.28)	0.13 (-0.93, 1.21)	595 (480, 737)	3703.17 (3003.44, 4490.26)	798 (572, 1107)	4092.3 (2962.88, 5655.65)	0.03 (-0.43, 0.49)	72 (47, 100)	441.47 (296.67, 618.03)	94 (56, 147)	487.63 (292, 767.23)	0.02 (-1.14, 1.19)
**Palestine**	17044 (13616, 21315)	748.4 (603.28, 938.42)	51315 (36397, 70569)	921.22 (657.65, 1246.88)	0.20 (-0.02, 0.43)	104006 (82306, 132059)	5291.74 (4278.37, 6496.06)	327024 (230094, 445128)	6302.59 (4552.02, 8392.6)	0.17 (0.09, 0.25)	12532 (8317, 17874)	628.47 (422.8, 879.63)	39245 (23650, 60192)	747.83 (456.99, 1112.11)	0.15 (-0.08, 0.38)
**Panama**	14825 (11876, 19275)	579.5 (469.7, 735.34)	31901 (22448, 43616)	735.73 (517.38, 1003.39)	0.30 (0.16, 0.45)	90434 (72917, 113512)	3723.63 (3055.12, 4565.32)	203767 (143351, 283361)	4660.89 (3276.47, 6486.3)	0.33 (0.28, 0.38)	10888 (7075, 15467)	445.01 (296.81, 619.22)	24250 (14679, 35506)	554.89 (335.87, 813.6)	0.32 (0.17, 0.47)
**Papua New Guinea**	24698 (19874, 31227)	568.36 (470.38, 714.99)	66702 (46249, 93985)	631.21 (441.91, 879.02)	0.06 (-0.14, 0.25)	144005 (113944, 181753)	3683.87 (2980.9, 4537.75)	395178 (274866, 542256)	4050.25 (2847.17, 5482.52)	0.08 (-0.01, 0.16)	17293 (11277, 24964)	435.87 (291.67, 606.77)	47552 (28037, 75252)	481.37 (287.66, 739.28)	0.11 (-0.04, 0.25)
**Paraguay**	36779 (29538, 47334)	868.85 (698.72, 1099.93)	83865 (59468, 115930)	1123.41 (804.57, 1552.53)	0.21 (0.02, 0.40)	250029 (203569, 312103)	6664.04 (5445.7, 8165.9)	614406 (442980, 821749)	8390.01 (6086.82, 11223.56)	0.24 (0.21, 0.27)	29891 (19980, 41588)	789.53 (533.57, 1081.04)	72904 (46827, 111933)	992.12 (637.27, 1514.56)	0.23 (0.14, 0.33)
**Peru**	168871 (134012, 220809)	741.97 (596.87, 954.76)	395172 (272594, 567762)	1065.08 (735.89, 1527.37)	0.38 (0.21, 0.55)	1088249 (876621, 1397137)	5230.94 (4228.6, 6433.32)	2708037 (1855795, 3780098)	7240.51 (4964.95, 10101.73)	0.39 (0.31, 0.46)	130741 (86830, 184416)	623 (420.53, 874.98)	324259 (197583, 487545)	866.21 (530.3, 1296.62)	0.40 (0.32, 0.48)
**Philippines**	383282 (314872, 467878)	590.09 (489.57, 711.7)	873801 (721937, 1062959)	747.65 (622.73, 905.99)	0.18 (0, 0.35)	2259451 (1949338, 2626848)	3850.21 (3334.89, 4406.48)	5402474 (4629752, 6261821)	4782.23 (4109.45, 5518.02)	0.26 (0.17, 0.35)	271335 (187454, 370969)	456.21 (313.47, 615.73)	647452 (447579, 888892)	569.44 (394.79, 775.84)	0.29 (0.17, 0.40)
**Poland**	193308 (161592, 230114)	492.79 (411.4, 590.35)	239588 (200086, 283999)	651.23 (541.55, 784.89)	0.20 (0.12, 0.27)	1286850 (1116546, 1472035)	3191.74 (2771.24, 3666.94)	1701877 (1463503, 1971094)	4071.35 (3484.22, 4726.93)	0.16 (0.13, 0.20)	151511 (105116, 204893)	377.25 (261.44, 510.05)	199098 (136238, 268912)	484.95 (334.88, 674.77)	0.19 (0.15, 0.23)

Characteristics	Incidence					Prevalence					YLDs				
	1992		2021		1992–2021	1992		2021		1992–2021	1992		2021		1992–2021
	Number No. (95% UI)	ASR per 100,000 No. (95% UI)	Incident cases No. (95% UI)	ASR per 100,000 No. (95% UI)	Net drift (% per year)	Number No. (95% UI)	ASR per 100,000 No. (95% UI)	Number No. (95% UI)	ASR per 100,000 No. (95% UI)	Net drift (% per year)	Number No. (95% UI)	ASR per 100,000 No. (95% UI)	Number No. (95% UI)	ASR per 100,000 No. (95% UI)	Net drift (% per year)
**Portugal**	95608 (78923, 119037)	978.76 (807.87, 1222.11)	110473 (80371, 151522)	1290.64 (929.74, 1775.32)	0.28 (0.01, 0.56)	825816 (681720, 1006953)	7625.33 (6233.28, 9361.57)	1055767 (761305, 1395471)	9712.44 (6864.89, 13062.42)	0.32 (0.25, 0.40)	97421 (65794, 134412)	905.47 (610.99, 1253.73)	123299 (74516, 182077)	1156.75 (692.93, 1723.82)	0.33 (0.25, 0.41)
**Puerto Rico**	24252 (19752, 30455)	650.78 (530.57, 815.63)	23782 (17110, 32390)	768.51 (550.56, 1045.65)	0.15 (0.06, 0.25)	164894 (134669, 200276)	4384.37 (3579.97, 5329.55)	183265 (131023, 242913)	5103.72 (3641.17, 6870.42)	0.17 (0.13, 0.21)	19628 (13089, 27049)	521.94 (347.66, 719.29)	21441 (13277, 31783)	607.48 (372.43, 914.91)	0.17 (0.06, 0.28)
**Qatar**	3389 (2701, 4294)	689.99 (565.13, 860.81)	23539 (15859, 32553)	773.42 (547.49, 1069.2)	0.16 (-0.17, 0.50)	21333 (16800, 27130)	4437.54 (3579.29, 5409.21)	155078 (107588, 219879)	4892.21 (3499.36, 6708.93)	0.07 (-0.14, 0.27)	2574 (1686, 3721)	530.66 (357.22, 748.2)	18560 (11154, 27035)	583.07 (362.99, 829.7)	-0.04 (-0.41, 0.32)
**Republic of Korea**	237705 (191925, 307951)	503.68 (408.03, 638.55)	263675 (185015, 361357)	557.56 (390.36, 779.64)	0.05 (-0.64, 0.74)	1542791 (1249713, 1923121)	3257.05 (2675.58, 4004.67)	1953265 (1392994, 2607292)	3549.72 (2490.06, 4794.05)	0.06 (-0.20, 0.32)	185721 (122412, 261462)	390.05 (260.45, 548.55)	231494 (141666, 348642)	427.19 (257.21, 656.73)	0.07 (-0.22, 0.35)
**Republic of Moldova**	25250 (20638, 31870)	561.23 (457.04, 709.87)	25425 (17599, 34671)	745.74 (514.25, 1020.42)	0.21 (0.10, 0.32)	172739 (140249, 210613)	3826.05 (3112.11, 4638.84)	190423 (136591, 254570)	4906.16 (3456.03, 6594.21)	0.18 (0.13, 0.23)	20514 (13707, 27949)	454.61 (303.61, 617.9)	22393 (14120, 33036)	585.47 (368.72, 872.06)	0.19 (0.06, 0.31)
**Romania**	116311 (95004, 146286)	488.39 (400.19, 616.43)	120068 (84440, 160210)	677.97 (463.34, 930.51)	0.25 (0.10, 0.40)	794542 (642700, 974965)	3198.23 (2591.39, 3929.62)	872769 (601135, 1159137)	4284.23 (2954.97, 5872.74)	0.27 (0.21, 0.32)	93769 (63575, 128492)	379.04 (254.18, 524.54)	102273 (63787, 153653)	511.06 (308.42, 768.28)	0.29 (0.23, 0.35)
**Russian Federation**	792842 (664205, 948574)	513.79 (430.14, 620.82)	977668 (805754, 1160073)	703.82 (582.55, 842.15)	0.24 (0.13, 0.35)	5350699 (4656651, 6124503)	3325.05 (2888.59, 3821.51)	6735034 (5853092, 7782235)	4395.19 (3804.7, 5077.53)	0.18 (0.14, 0.22)	628766 (437282, 848356)	392.84 (272.38, 531.35)	787482 (545601, 1060552)	521.98 (359.28, 709.56)	0.19 (0.15, 0.24)
**Rwanda**	38723 (30949, 50616)	536.13 (431.84, 686.7)	89552 (64357, 127027)	650.23 (467.55, 892.94)	0.18 (-0.01, 0.36)	217537 (171762, 277747)	3474.47 (2780.62, 4304.72)	527657 (373649, 732411)	4184.19 (2956.27, 5698.98)	0.24 (0.16, 0.31)	26065 (16975, 36891)	410.79 (275.01, 566.5)	63245 (37268, 95625)	495.99 (291.32, 740.9)	0.24 (0.03, 0.44)
**Saint Kitts and Nevis**	266 (216, 343)	639.03 (520.33, 811.83)	461 (330, 643)	766.18 (554.05, 1075.16)	0.22 (-0.47, 0.91)	1707 (1387, 2105)	4278.33 (3471.37, 5232.43)	3266 (2343, 4405)	5044.03 (3591.63, 6839.66)	0.17 (-0.12, 0.46)	203 (135, 283)	506.5 (338.18, 698.21)	385 (239, 565)	598.12 (371.73, 888.48)	0.17 (-0.57, 0.91)
**Saint Lucia**	904 (728, 1175)	639.7 (522.54, 812.86)	1494 (1061, 2061)	840.12 (598.43, 1154.89)	0.15 (-0.29, 0.60)	5626 (4533, 7073)	4294.15 (3488.64, 5295.44)	10589 (7717, 14137)	5464.86 (3926.46, 7406.8)	0.19 (0.01, 0.36)	672 (442, 945)	508.27 (340.52, 702.7)	1246 (787, 1851)	648.01 (408.45, 961.41)	0.20 (-0.23, 0.64)
**Saint Vincent and the Grenadines**	712 (573, 908)	636.95 (520.45, 805.78)	914 (647, 1268)	797 (560.99, 1107.96)	0.11 (-0.41, 0.63)	4409 (3571, 5546)	4255.09 (3465.51, 5199.28)	6255 (4422, 8369)	5195.44 (3682.42, 6956.51)	0.10 (-0.15, 0.36)	528 (349, 757)	505.52 (334.95, 707.92)	737 (455, 1073)	615.54 (380.17, 897.16)	0.19 (-0.31, 0.69)
**Samoa**	973 (783, 1215)	565.21 (466.27, 708.29)	1422 (1025, 1971)	658.45 (477.13, 911.3)	0.06 (-0.42, 0.55)	5701 (4541, 7194)	3681.97 (2992.52, 4504.72)	8509 (6097, 11614)	4226.48 (3078.86, 5671)	0.11 (-0.14, 0.35)	690 (454, 999)	439.15 (290.22, 609.21)	1022 (621, 1545)	503.17 (310.37, 754.83)	0.12 (-0.34, 0.59)
**San Marino**	184 (150, 231)	796.01 (655.94, 999.73)	279 (205, 377)	1014.65 (742.52, 1383.94)	0.16 (-0.60, 0.92)	1440 (1181, 1746)	5507.02 (4509.77, 6785.98)	2288 (1641, 3037)	6854.65 (4884.34, 9048.56)	0.21 (-0.06, 0.49)	171 (116, 237)	658.89 (440.46, 919.92)	269 (170, 399)	819.76 (516, 1237.12)	0.10 (-0.63, 0.84)
**Sao Tome and Principe**	589 (472, 740)	466.74 (382.19, 588.61)	1179 (829, 1617)	516.22 (369.44, 706.11)	0.16 (-0.41, 0.74)	3263 (2562, 4195)	2904.49 (2330.58, 3588.57)	6857 (4771, 9296)	3183.37 (2204.36, 4317.52)	0.11 (-0.14, 0.37)	394 (258, 571)	346.68 (230.42, 489.11)	826 (505, 1231)	379.75 (235.67, 567.3)	0.09 (-0.55, 0.73)
**Saudi Arabia**	125526 (100054, 157226)	693.79 (565.7, 877.61)	310803 (223060, 432305)	788.51 (572.31, 1076.11)	0.23 (0.13, 0.33)	756154 (590916, 949447)	4581.05 (3701.56, 5631.7)	2108231 (1495959, 2885792)	5144.07 (3714.57, 6929.05)	0.19 (0.13, 0.24)	91299 (59368, 132975)	545.46 (366.33, 778.55)	251809 (157586, 378772)	612.79 (381.21, 918.18)	0.17 (0.07, 0.28)
**Senegal**	36425 (29170, 45821)	457.91 (374.97, 575.94)	92528 (65174, 129263)	560.37 (395.77, 769.79)	0.17 (0.04, 0.31)	199995 (158509, 254204)	2825.01 (2274.49, 3477.82)	518879 (368788, 723247)	3393.86 (2448.52, 4619.89)	0.17 (0.11, 0.23)	23948 (15734, 34574)	334.35 (220.98, 469.69)	62342 (36843, 96241)	403.5 (246.72, 620.17)	0.20 (0.02, 0.37)

Characteristics	Incidence					Prevalence					YLDs				
	1992		2021		1992–2021	1992		2021		1992–2021	1992		2021		1992–2021
	Number No. (95% UI)	ASR per 100,000 No. (95% UI)	Incident cases No. (95% UI)	ASR per 100,000 No. (95% UI)	Net drift (% per year)	Number No. (95% UI)	ASR per 100,000 No. (95% UI)	Number No. (95% UI)	ASR per 100,000 No. (95% UI)	Net drift (% per year)	Number No. (95% UI)	ASR per 100,000 No. (95% UI)	Number No. (95% UI)	ASR per 100,000 No. (95% UI)	Net drift (% per year)
**Serbia**	49258 (40382, 60762)	497.25 (405.21, 619.4)	56493 (39700, 75203)	653.83 (457.47, 871.62)	0.22 (0.15, 0.30)	340955 (278539, 414661)	3285.78 (2702.65, 3997.05)	412390 (295187, 563643)	4188.18 (3026.52, 5741.14)	0.20 (0.15, 0.24)	40398 (27312, 55456)	391.07 (263.97, 536.55)	48398 (29806, 72997)	499.75 (306.27, 768.89)	0.19 (0.11, 0.28)
**Seychelles**	402 (324, 512)	535.69 (437.4, 673.67)	744 (543, 1010)	697.26 (502.64, 935.86)	0.09 (-0.49, 0.67)	2506 (2048, 3123)	3473.07 (2855.35, 4252.95)	4851 (3544, 6595)	4361.59 (3217.46, 5934.71)	0.17 (-0.09, 0.43)	301 (200, 422)	414.78 (279.57, 576.27)	578 (351, 877)	521.46 (318.96, 797.74)	0.01 (-0.60, 0.62)
**Sierra Leone**	20199 (16257, 25286)	496.04 (404.21, 622.39)	46948 (32870, 65963)	516.62 (370.33, 708.08)	0.11 (-0.05, 0.28)	116737 (93951, 146673)	3162.04 (2584.07, 3873.37)	274466 (193932, 377700)	3282.1 (2336.46, 4460.64)	0.12 (0.06, 0.19)	13930 (9184, 19715)	373.5 (247.99, 518.88)	32952 (20719, 50739)	389.04 (247.75, 615.48)	0.14 (-0.06, 0.34)
**Singapore**	15561 (12501, 20025)	464.19 (379.13, 583.77)	27370 (19350, 37473)	510.3 (364.27, 699.95)	0.01 (-0.13, 0.14)	99145 (80107, 123749)	2860.36 (2326.6, 3493.6)	185207 (131480, 250585)	3104.54 (2203.92, 4193.67)	-0.03 (-0.08, 0.02)	11991 (7922, 17302)	344.97 (231.51, 486.64)	22176 (13616, 33608)	375.49 (229.03, 574.77)	-0.02 (-0.18, 0.13)
**Slovakia**	27410 (22418, 34498)	504.84 (409.95, 638.42)	36061 (26339, 48689)	699.95 (506.35, 956.61)	0.17 (0.01, 0.32)	188799 (153775, 229609)	3378.06 (2755.77, 4104.88)	262516 (193656, 352032)	4482.53 (3230.74, 6052.68)	0.16 (0.13, 0.20)	22317 (14996, 30783)	400.43 (267.47, 551.99)	30820 (19516, 45989)	534.88 (331.62, 800.45)	0.18 (0.09, 0.27)
**Slovenia**	10203 (8320, 12746)	502.43 (411.9, 629.4)	12222 (8658, 16502)	633.71 (452.41, 875.1)	0.17 (-0.03, 0.37)	72460 (59507, 88599)	3358.5 (2769.24, 4114.86)	91999 (66126, 125465)	4076.45 (2934.59, 5625.38)	0.12 (0.08, 0.17)	8533 (5777, 11814)	397.99 (270.55, 551.56)	10745 (6234, 16083)	486.73 (286.72, 730.35)	0.15 (0.01, 0.29)
**Solomon Islands**	2042 (1616, 2587)	569.63 (463.7, 716.3)	4638 (3292, 6526)	662.65 (477.07, 929.82)	0.13 (-0.18, 0.44)	11600 (9140, 14833)	3668.65 (3004.05, 4506.82)	27322 (19515, 38436)	4248.99 (3060.74, 5848.69)	0.18 (0.01, 0.35)	1404 (914, 2019)	437.07 (295.28, 613.39)	3292 (2112, 5074)	505.75 (324.67, 773.13)	0.17 (-0.19, 0.52)
**Somalia**	38648 (30858, 50154)	523.75 (421.86, 666.45)	142398 (98729, 193979)	665.45 (460.02, 899.91)	0.18 (-0.03, 0.39)	213118 (167883, 268577)	3361.34 (2695.19, 4160.35)	769029 (534570, 1066654)	4186.2 (2937.89, 5741.84)	0.22 (0.13, 0.31)	25474 (16376, 36442)	396.32 (260.12, 553.86)	92097 (57171, 138749)	493.78 (316.44, 726.49)	0.24 (0.08, 0.40)
**South Africa**	225264 (184322, 275729)	573.15 (475.3, 693.55)	448967 (365264, 556147)	758.65 (619.89, 932.49)	0.22 (-0.11, 0.54)	1362265 (1171146, 1582148)	3735.46 (3220.09, 4300.79)	2841770 (2373320, 3415773)	4825.58 (4040.9, 5791.71)	0.20 (0.09, 0.31)	162184 (111278, 224663)	440.12 (304.21, 600.48)	333284 (228251, 458835)	564.55 (388.29, 767.45)	0.17 (0.05, 0.29)
**South Sudan**	35645 (28088, 45940)	596.83 (482.5, 755.22)	64311 (43713, 88618)	666.21 (461.33, 913.45)	0.19 (0.04, 0.35)	210204 (165705, 270650)	4009.85 (3257.98, 4965.25)	381780 (270770, 528296)	4528.92 (3184.27, 6183.02)	0.27 (0.21, 0.33)	25019 (16387, 35731)	470.66 (311.66, 657.22)	45538 (27033, 71510)	532.39 (315.88, 830.76)	0.27 (0.08, 0.45)
**Spain**	261520 (213964, 331492)	688.14 (560.58, 866.97)	349472 (253185, 466837)	902.48 (642.61, 1224.56)	0.47 (0.32, 0.63)	1950172 (1613768, 2365525)	4658.9 (3827.19, 5738.91)	2771314 (2039679, 3723771)	5984.87 (4311.5, 8097.35)	0.76 (0.68, 0.84)	231225 (155394, 319791)	556.33 (369.16, 781.23)	325127 (203456, 477162)	714.79 (443.66, 1052.49)	0.75 (0.67, 0.84)
**Sri Lanka**	98492 (79729, 126667)	547.17 (446.87, 689.76)	158395 (112274, 215787)	704.72 (496.56, 956.3)	0.17 (0.03, 0.31)	607452 (497025, 758074)	3544.43 (2880.09, 4360.89)	1057824 (735664, 1415915)	4502.79 (3161.09, 6018.81)	0.30 (0.27, 0.34)	72743 (48176, 100830)	420.95 (282.69, 580.94)	125460 (76727, 188623)	536.55 (326.53, 812.68)	0.30 (0.22, 0.38)
**Sudan**	158411 (126375, 200676)	711.06 (575.82, 908.69)	403701 (280070, 577617)	858.75 (612.38, 1211.25)	0.20 (0.02, 0.39)	966062 (757891, 1220937)	4859.99 (3939.65, 5990.97)	2497205 (1789131, 3544144)	5734.42 (4172.57, 8075.87)	0.17 (0.13, 0.22)	115889 (76389, 165604)	576 (383.45, 805.33)	300202 (185510, 468843)	681.86 (428.05, 1046.69)	0.17 (0.08, 0.26)
**Suriname**	2516 (2040, 3238)	632.1 (516.04, 799.97)	5042 (3516, 6918)	863.08 (596.46, 1181.18)	0.22 (-0.06, 0.50)	15817 (12951, 19873)	4186.15 (3428.21, 5141.31)	33464 (23685, 44724)	5575.55 (3957.39, 7482.75)	0.26 (0.14, 0.37)	1887 (1262, 2660)	496.3 (334.67, 690.64)	3943 (2471, 5801)	658.46 (412.59, 971.04)	0.16 (-0.19, 0.52)
**Sweden**	60819 (50986, 73242)	758.96 (634.15, 915.89)	83015 (62210, 106615)	897.29 (672.5, 1155.15)	0.20 (0.12, 0.28)	457296 (398546, 520133)	5067.37 (4405.78, 5830.48)	622064 (495006, 778853)	5890.21 (4632.59, 7457.93)	0.16 (0.14, 0.18)	54050 (37433, 72652)	606.32 (424.9, 831.11)	73309 (47872, 103894)	704.7 (458.19, 1009.98)	0.16 (0.11, 0.20)
**Switzerland**	58124 (47345, 72492)	908.5 (745.9, 1141.44)	80011 (58614, 108718)	1044.83 (753.6, 1420.74)	0.24 (0.15, 0.33)	495516 (403521, 605774)	6754.19 (5484.38, 8266.48)	702585 (517122, 931408)	7609.41 (5619.15, 10306.74)	0.25 (0.23, 0.27)	58242 (39182, 79927)	802.1 (534.34, 1123.15)	82213 (52215, 119713)	905.93 (576.21, 1353.26)	0.26 (0.21, 0.30)

Characteristics	Incidence					Prevalence					YLDs				
	1992		2021		1992–2021	1992		2021		1992–2021	1992		2021		1992–2021
	Number No. (95% UI)	ASR per 100,000 No. (95% UI)	Incident cases No. (95% UI)	ASR per 100,000 No. (95% UI)	Net drift (% per year)	Number No. (95% UI)	ASR per 100,000 No. (95% UI)	Number No. (95% UI)	ASR per 100,000 No. (95% UI)	Net drift (% per year)	Number No. (95% UI)	ASR per 100,000 No. (95% UI)	Number No. (95% UI)	ASR per 100,000 No. (95% UI)	Net drift (% per year)
**Syrian Arab Republic**	106838 (85215, 134045)	743.18 (605.45, 940.62)	134788 (96450, 183583)	923.99 (659.93, 1258.74)	0.26 (0.12, 0.39)	649039 (511923, 831496)	5170.9 (4171.62, 6393.92)	945251 (668283, 1288268)	6427.35 (4549.33, 8864.77)	0.23 (0.12, 0.33)	78371 (51639, 113211)	615.63 (414.51, 863.09)	112090 (70647, 167371)	759.83 (483.21, 1145.39)	0.21 (0.10, 0.33)
**Taiwan (Province of China)**	123488 (100105, 150360)	581.94 (475.97, 702.23)	132531 (94179, 180979)	592.66 (424.76, 808.33)	0 (-0.14, 0.13)	797745 (661252, 967845)	3739.13 (3135.33, 4497.31)	976511 (714917, 1310867)	3777.38 (2760.36, 5125.5)	-0.01 (-0.06, 0.04)	96701 (64408, 132887)	451.16 (302.48, 620.54)	115227 (72111, 170728)	454.35 (287.09, 694.46)	-0.01 (-0.06, 0.04)
**Tajikistan**	19111 (15227, 24479)	357.68 (291.16, 454.03)	47368 (32670, 66752)	461.78 (317.75, 643.39)	0.23 (0.09, 0.38)	105105 (84106, 132605)	2179.7 (1767.32, 2670.31)	268045 (187070, 370769)	2754.66 (1890.5, 3731.04)	0.17 (0.12, 0.23)	12682 (8411, 18136)	259.98 (174.64, 363.82)	32319 (19272, 50397)	329.54 (199.26, 504.61)	0.16 (0, 0.33)
**Thailand**	313454 (254086, 399421)	514.63 (418.5, 646.34)	404231 (282924, 575334)	604.26 (427.62, 852.74)	0.14 (0.08, 0.21)	1917820 (1547766, 2381878)	3267.34 (2647.67, 3986.26)	2835794 (2000806, 3850426)	3782.08 (2650.8, 5197.72)	0.14 (0.12, 0.15)	229732 (153207, 322338)	388.15 (260.29, 540.4)	334599 (202047, 515895)	451.78 (275.29, 702.4)	0.16 (0.12, 0.19)
**Timor-Leste**	4170 (3366, 5293)	535.12 (437.74, 666.59)	9185 (6473, 12156)	655.45 (467.19, 856.8)	0.23 (-0.06, 0.51)	23737 (19108, 29976)	3437.55 (2806.63, 4203.76)	53714 (38138, 72043)	4130.04 (2979.63, 5476.24)	0.20 (0.05, 0.35)	2849 (1868, 4026)	406.59 (276.45, 552.3)	6477 (3950, 9813)	492.54 (304.85, 746.14)	0.23 (-0.08, 0.53)
**Togo**	19077 (15194, 24284)	492.17 (399.98, 620.96)	46682 (32976, 64213)	538.13 (388.47, 731.8)	0.10 (-0.12, 0.32)	106442 (82892, 136586)	3128.57 (2517.12, 3875.21)	271213 (189810, 371172)	3405.51 (2380.18, 4558.65)	0.17 (0.08, 0.27)	12769 (8409, 18596)	370.13 (249.83, 517.39)	32565 (19975, 49841)	404.66 (250.83, 616.71)	0.20 (0.02, 0.37)
**Tokelau**	9 (7, 11)	570.06 (471.76, 710.84)	9 (7, 13)	654.92 (479.77, 922.97)	0.56 (-3.49, 4.78)	55 (44, 68)	3726.11 (3031.33, 4558.45)	59 (44, 80)	4214.79 (3092.42, 5705.41)	0.25 (-1.20, 1.73)	7 (4, 9)	444.25 (300.72, 620.5)	7 (4, 11)	501.67 (315.19, 747.29)	0.96 (-4.41, 6.62)
**Tonga**	567 (455, 716)	571 (468.97, 715.31)	706 (497, 967)	662.86 (465.92, 912.78)	0.12 (-0.43, 0.68)	3355 (2689, 4204)	3725.85 (3039.13, 4548.83)	4300 (3057, 5764)	4281.59 (3086.44, 5740.33)	0.15 (-0.13, 0.43)	406 (270, 572)	445.14 (301.44, 617.42)	517 (320, 790)	511.29 (313.44, 772.63)	0.17 (-0.42, 0.76)
**Trinidad and Tobago**	7710 (6266, 9876)	611.12 (499.85, 777.52)	11658 (8153, 15967)	846.95 (587.14, 1166.35)	0.24 (0.03, 0.46)	47870 (38475, 59571)	3990.28 (3248.93, 4909.66)	79563 (57987, 111021)	5387.18 (3910.88, 7460.39)	0.25 (0.16, 0.34)	5719 (3819, 8089)	473.8 (321.17, 662.86)	9361 (6003, 14066)	639.02 (409.49, 973.75)	0.27 (0.10, 0.44)
**Tunisia**	66660 (53782, 83812)	725.61 (596.46, 916.86)	118721 (83853, 168901)	1025.71 (722.51, 1475.05)	0.29 (0.19, 0.38)	431847 (341858, 540083)	5003.23 (4052.32, 6138.19)	826676 (598155, 1122049)	6875.36 (4987.43, 9496.72)	0.32 (0.25, 0.38)	51972 (34106, 73284)	596.87 (396.58, 827.55)	97771 (61489, 144542)	816.84 (506.57, 1206.5)	0.28 (0.16, 0.39)
**Turkmenistan**	388240 (329711, 451551)	620.02 (530.94, 726.12)	23199 (16647, 31381)	442.45 (316.72, 596.45)	0.21 (0.04, 0.39)	2396822 (2169961, 2638627)	3992.9 (3657.36, 4379.18)	136092 (96077, 182140)	2643.82 (1887.84, 3529.92)	0.22 (0.15, 0.28)	288854 (202598, 390547)	477.27 (337.14, 638.99)	16324 (9570, 24675)	315.95 (185, 475.16)	0.23 (0.03, 0.43)
**Tuvalu**	13208 (10625, 16837)	352.81 (288.06, 438.92)	82 (59, 117)	654.72 (470.36, 924.17)	0.19 (-1.19, 1.59)	73579 (58555, 93726)	2143.71 (1725.06, 2619.2)	520 (367, 714)	4211.59 (2983.44, 5734.77)	-0.06 (-0.64, 0.53)	8862 (5718, 12633)	255.27 (169.72, 357.48)	62 (38, 94)	502.16 (304.68, 759.01)	-0.07 (-1.61, 1.50)
**Türkiye**	53 (43, 67)	572.32 (464.99, 712.87)	698666 (503758, 964631)	841.34 (608.36, 1151.42)	1.64 (1.15, 2.13)	330 (265, 406)	3771.05 (3074.03, 4570.12)	4694266 (3283843, 6411243)	5420.06 (3786.57, 7370.38)	1.80 (1.61, 1.99)	39 (26, 55)	447.64 (301.37, 619.54)	559351 (332345, 830643)	648.12 (385.2, 958.6)	1.79 (1.58, 2.00)
**Uganda**	93855 (74274, 121884)	546.29 (443.09, 700.25)	291916 (208121, 411833)	669.78 (482.67, 926.49)	0.26 (0.16, 0.36)	523334 (410993, 677745)	3534.23 (2831.98, 4400.32)	1631846 (1168621, 2246871)	4329.31 (3104.53, 5764.86)	0.37 (0.32, 0.42)	62161 (40391, 87689)	414 (273.76, 573.16)	196623 (117125, 295283)	513.68 (314.71, 760.22)	0.41 (0.29, 0.53)
**Ukraine**	256705 (217695, 307445)	485.61 (407.13, 582.72)	270965 (194341, 352040)	654.83 (475.42, 861.32)	0.34 (0.17, 0.51)	1732334 (1517158, 1980615)	3088.18 (2698.62, 3563.9)	1877242 (1408155, 2386328)	4028.17 (3012.58, 5203.31)	0.35 (0.27, 0.42)	202865 (142360, 274324)	364.89 (254.24, 497.86)	219303 (138377, 314425)	478.86 (302.2, 690.35)	0.37 (0.30, 0.45)
**United Arab Emirates**	14864 (11943, 18676)	673.35 (555.83, 838.83)	79861 (54355, 114045)	835.42 (606.53, 1165.59)	0.21 (-0.03, 0.44)	90425 (73148, 112654)	4273.67 (3510.29, 5186.88)	519114 (358345, 711671)	5168.1 (3648.71, 7062.59)	0 (-0.42, 0.43)	10917 (7240, 15343)	510.54 (349.77, 711.76)	61832 (37158, 96015)	618.4 (374.21, 940.32)	0.05 (-0.16, 0.26)

Characteristics	Incidence					Prevalence					YLDs				
	1992		2021		1992–2021	1992		2021		1992–2021	1992		2021		1992–2021
	Number No. (95% UI)	ASR per 100,000 No. (95% UI)	Incident cases No. (95% UI)	ASR per 100,000 No. (95% UI)	Net drift (% per year)	Number No. (95% UI)	ASR per 100,000 No. (95% UI)	Number No. (95% UI)	ASR per 100,000 No. (95% UI)	Net drift (% per year)	Number No. (95% UI)	ASR per 100,000 No. (95% UI)	Number No. (95% UI)	ASR per 100,000 No. (95% UI)	Net drift (% per year)
**United Kingdom**	374407 (311479, 453122)	691.42 (577.61, 839.45)	543893 (453467, 652957)	882.38 (738.46, 1064.01)	0.41 (0.27, 0.55)	2618897 (2283055, 3007805)	4412.16 (3818.05, 5090.48)	3901117 (3390525, 4449002)	5601.04 (4832.57, 6488.41)	0.45 (0.39, 0.50)	309263 (213436, 418190)	525.89 (361.54, 720.42)	457793 (316664, 626362)	666.56 (463.96, 909.62)	0.44 (0.38, 0.50)
**United Republic of Tanzania**	143192 (114261, 187181)	547.37 (441.74, 705.93)	383377 (259607, 537258)	647.81 (446.82, 898.89)	0.20 (0.13, 0.28)	816139 (648910, 1047628)	3566.42 (2909.06, 4455.65)	2219009 (1548777, 3094291)	4191.81 (2968.07, 5793.52)	0.24 (0.19, 0.28)	96373 (62833, 135437)	416.54 (276.03, 574.65)	265812 (159251, 424219)	496.55 (305.8, 780.78)	0.29 (0.19, 0.38)
**United States of America**	1926644 (1584105, 2363389)	729.59 (602.32, 894.89)	3095768 (2496885, 3731330)	980.77 (792.69, 1188.66)	0.14 (-0.35, 0.63)	14406055 (12414235, 16593992)	5175.58 (4442.22, 5984.77)	24303559 (20888275, 27975340)	6865.24 (5935.69, 7971.2)	0.20 (0.01, 0.39)	1692415 (1163101, 2275347)	610.47 (422, 825.71)	2814409 (1928982, 3792632)	803.98 (555.33, 1093.96)	0.27 (-0.21, 0.75)
**United States Virgin Islands**	698 (566, 887)	641.04 (521.63, 817.31)	628 (455, 841)	803.37 (580.15, 1091.5)	0.08 (-0.12, 0.28)	4627 (3733, 5670)	4298.27 (3480.73, 5279.73)	4795 (3493, 6339)	5276.72 (3859.99, 7045.51)	-0.09 (-0.18, 0)	552 (364, 768)	511.96 (339.01, 708.6)	559 (354, 813)	626.99 (389.56, 928.17)	-0.13 (-0.23, -0.04)
**Uruguay**	22051 (17892, 28223)	708.73 (573.59, 911.08)	29716 (21385, 40108)	931.84 (657.43, 1270.89)	0.15 (-0.02, 0.32)	165359 (135326, 203995)	5137.11 (4183.91, 6393.75)	232347 (171006, 311052)	6609.91 (4857.73, 8825.09)	0.21 (0.18, 0.25)	19671 (13164, 26801)	613.16 (407.84, 841.71)	27437 (16872, 40742)	787.29 (487, 1174.01)	0.21 (0.11, 0.30)
**Uzbekistan**	71445 (57722, 90782)	336.37 (275.31, 421.78)	137014 (97827, 185446)	397.67 (284.84, 540.54)	0.14 (0, 0.29)	392990 (314561, 496446)	2010.31 (1625.9, 2468.37)	793788 (570761, 1101689)	2339.51 (1691.15, 3233.04)	0.11 (0.08, 0.15)	47188 (31268, 66282)	238.87 (159.76, 331.57)	95096 (57457, 152434)	279.36 (169.19, 447.37)	0.12 (0.04, 0.20)
**Vanuatu**	897 (719, 1142)	569.09 (464.23, 713.12)	2127 (1481, 2961)	664.34 (470.14, 908.95)	0.12 (-0.32, 0.57)	5129 (4062, 6497)	3680.66 (3001.57, 4513.19)	12645 (8890, 17600)	4277.06 (3055.42, 5849.99)	0.18 (-0.06, 0.42)	620 (403, 882)	438.68 (293.84, 606.34)	1526 (903, 2328)	510.41 (305.66, 768.87)	0.07 (-0.42, 0.56)
**Venezuela (Bolivarian Republic of)**	120556 (97479, 155979)	597.23 (485.67, 761.14)	182162 (130952, 251027)	685.36 (489.99, 934.9)	0.17 (0.06, 0.27)	729505 (584435, 918913)	3893.29 (3152.21, 4786.82)	1220334 (873020, 1663971)	4465.94 (3177.98, 6118.14)	0.23 (0.20, 0.25)	87718 (57319, 125225)	463.49 (313.11, 642.6)	144804 (91447, 217457)	531.71 (333.06, 796.1)	0.23 (0.17, 0.29)
**Viet Nam**	260913 (210154, 324488)	374.6 (307.28, 462.14)	464255 (331256, 628586)	452.09 (321.55, 612.97)	0.10 (-0.17, 0.37)	1403695 (1135022, 1758328)	2174.56 (1789.74, 2668.09)	2677186 (1919933, 3585958)	2572.07 (1839.88, 3460.28)	0.06 (-0.06, 0.18)	169334 (112551, 238013)	259.68 (177.07, 363.68)	321630 (204153, 496452)	309.09 (195.72, 476.34)	0.07 (-0.06, 0.21)
**Yemen**	108586 (86347, 137228)	716.35 (585.08, 908.67)	278881 (198872, 387103)	769.08 (553.55, 1044.65)	0.12 (0.01, 0.23)	621891 (487271, 791045)	4925.32 (3980.9, 6087.78)	1702674 (1218061, 2315536)	5202.99 (3785.27, 6971.28)	0.01 (-0.03, 0.05)	74221 (48280, 106996)	578.87 (389.3, 809.78)	202561 (122312, 306292)	611.69 (374.89, 903.7)	0.01 (-0.11, 0.14)
**Zambia**	43816 (34246, 57068)	549.8 (443.24, 703.57)	141135 (97561, 200522)	698.68 (487.88, 1000.79)	0.23 (0.08, 0.39)	247468 (193494, 320168)	3576.4 (2880.44, 4462.62)	804953 (558361, 1111078)	4504.8 (3158.04, 6129.37)	0.38 (0.31, 0.45)	29403 (19381, 41567)	419.63 (282.27, 574.85)	96200 (58363, 150172)	531.01 (330.88, 836.64)	0.36 (0.17, 0.55)
**Zimbabwe**	54711 (43443, 69461)	510.04 (415.49, 645.24)	102882 (71505, 146768)	636.62 (449.17, 883.6)	0.15 (0.02, 0.28)	300649 (238865, 383789)	3173.54 (2581.83, 3910.79)	575166 (395286, 804358)	3912.83 (2749.35, 5401.1)	0.18 (0.13, 0.23)	36117 (23907, 52060)	375.76 (248.79, 522.38)	68943 (41536, 102713)	462.98 (280.44, 681.62)	0.16 (0, 0.32)

Notes: ASR, age-standardized rate; YLDs, years lived with disability; CI, confidence interval; UI, uncertainty interval.
Source: Institute for Health Metrics and Evaluation. Used with permission. All rights reserved.

Table S3. Fitted longitudinal age effects of the incidence, prevalence, and years lived with disability of anxiety disorders (per 100,000 person-years) and the corresponding 95% CIs stratified by sex.

Age group (years)	Both	Male	Female
**Incidence**			
0-4	81.07 (78.85, 83.36)	65.30 (63.55, 67.11)	97.96 (94.97, 101.05)
5-9	503.23 (494.03, 512.60)	405.12 (397.89, 412.48)	608.34 (595.93, 621.02)
10-14	700.55 (688.62, 712.68)	561.07 (551.76, 570.54)	849.98 (833.80, 866.46)
15-19	643.47 (632.76, 654.35)	505.77 (497.55, 514.12)	788.72 (774.08, 803.65)
20-24	622.00 (611.92, 632.25)	486.56 (478.84, 494.40)	761.30 (747.53, 775.32)
25-29	642.46 (632.40, 652.68)	510.15 (502.36, 518.07)	777.60 (763.99, 791.44)
30-34	663.73 (653.68, 673.94)	534.89 (527.01, 542.88)	796.15 (782.64, 809.89)
35-39	686.48 (677.05, 696.04)	561.70 (554.21, 569.28)	815.41 (802.84, 828.18)
40-44	675.02 (665.89, 684.28)	568.92 (561.50, 576.44)	784.95 (772.98, 797.10)
45-49	630.14 (621.42, 638.98)	555.94 (548.59, 563.38)	706.61 (695.50, 717.88)
50-54	583.12 (574.57, 591.79)	540.82 (533.35, 548.39)	626.06 (615.53, 636.77)
55-59	532.91 (524.30, 541.67)	523.11 (515.29, 531.05)	542.54 (532.30, 552.97)
60-64	479.36 (470.58, 488.30)	490.51 (482.31, 498.85)	468.41 (458.28, 478.77)
65-69	424.05 (414.31, 434.01)	444.01 (434.71, 453.52)	405.26 (394.27, 416.56)
70-74	366.95 (356.74, 377.45)	396.80 (386.76, 407.09)	340.53 (329.29, 352.14)
75-79	308.10 (297.29, 319.30)	349.66 (338.53, 361.16)	274.73 (263.30, 286.66)
80-84	240.77 (229.22, 252.91)	284.25 (271.67, 297.41)	210.23 (198.44, 222.71)
85-89	168.64 (155.72, 182.62)	201.91 (186.98, 218.03)	148.71 (135.91, 162.71)
90-94	99.01 (83.83, 116.94)	120.48 (101.45, 143.07)	88.24 (73.76, 105.56)
95+	32.34 (18.92, 55.29)	39.99 (22.22, 72.00)	28.98 (16.59, 50.61)
**Prevalence**			
0-4	80.34 (75.69, 85.28)	62.95 (59.82, 66.25)	98.97 (92.34, 106.09)
5-9	1230.72 (1204.44, 1257.58)	944.68 (926.97, 962.73)	1537.26 (1499.70, 1575.76)
10-14	3216.19 (3161.09, 3272.25)	2439.77 (2402.70, 2477.40)	4047.64 (3968.82, 4128.02)
15-19	4217.82 (4150.62, 4286.10)	3134.11 (3089.58, 3179.27)	5359.98 (5263.27, 5458.46)
20-24	4423.76 (4356.14, 4492.43)	3225.31 (3181.16, 3270.08)	5656.28 (5558.63, 5755.65)
25-29	4518.38 (4451.89, 4585.86)	3270.02 (3226.82, 3313.79)	5794.99 (5698.81, 5892.79)
30-34	4622.81 (4557.17, 4689.39)	3350.40 (3307.66, 3393.68)	5932.33 (5837.37, 6028.83)
35-39	4760.42 (4699.02, 4822.62)	3471.15 (3431.01, 3511.76)	6093.44 (6004.73, 6183.46)
40-44	4883.42 (4822.10, 4945.53)	3588.36 (3547.90, 3629.28)	6223.90 (6135.76, 6313.31)
45-49	4912.10 (4850.30, 4974.68)	3627.69 (3586.63, 3669.21)	6229.40 (6141.12, 6318.96)
50-54	4869.36 (4805.95, 4933.61)	3610.43 (3568.09, 3653.28)	6135.58 (6045.63, 6226.87)
55-59	4782.56 (4715.73, 4850.34)	3563.62 (3518.73, 3609.09)	5976.51 (5882.54, 6071.97)
60-64	4679.67 (4607.98, 4752.46)	3505.77 (3457.21, 3555.01)	5792.74 (5693.08, 5894.14)
65-69	4595.84 (4510.00, 4683.32)	3432.11 (3373.69, 3491.55)	5656.20 (5538.07, 5776.85)
70-74	4516.67 (4419.47, 4616.00)	3338.49 (3272.03, 3406.30)	5529.60 (5397.57, 5664.86)
75-79	4451.43 (4338.66, 4567.13)	3242.43 (3164.41, 3322.36)	5402.37 (5252.36, 5556.65)
80-84	4357.86 (4222.03, 4498.06)	3093.01 (2996.90, 3192.19)	5227.03 (5052.01, 5408.12)
85-89	4058.83 (3885.46, 4239.93)	2719.25 (2592.76, 2851.92)	4822.74 (4608.27, 5047.19)
90-94	3562.44 (3314.77, 3828.62)	2180.73 (1992.48, 2386.77)	4194.47 (3902.46, 4508.33)

Age group (years)	Both	Male	Female
95+	2890.27 (2501.73, 3339.15)	1577.68 (1281.12, 1942.87)	3392.78 (2949.49, 3902.70)
	YLDs		
0-4	10.01 (9.44, 10.61)	7.83 (7.44, 8.23)	12.35 (11.54, 13.21)
5-9	153.51 (150.28, 156.80)	117.58 (115.39, 119.81)	192.03 (187.42, 196.76)
10-14	398.39 (391.64, 405.27)	302.48 (297.89, 307.14)	501.14 (491.47, 511.01)
15-19	517.42 (509.23, 525.74)	386.53 (381.03, 392.11)	655.40 (643.64, 667.38)
20-24	537.93 (529.75, 546.24)	395.51 (390.08, 401.02)	684.45 (672.67, 696.44)
25-29	544.89 (536.90, 553.00)	398.56 (393.28, 403.92)	694.57 (683.05, 706.27)
30-34	553.90 (546.05, 561.87)	406.12 (400.91, 411.39)	706.01 (694.71, 717.50)
35-39	566.58 (559.28, 573.97)	418.44 (413.57, 423.36)	719.75 (709.26, 730.40)
40-44	577.04 (569.78, 584.38)	429.71 (424.83, 434.65)	729.52 (719.16, 740.04)
45-49	576.14 (568.87, 583.51)	430.98 (426.05, 435.96)	725.03 (714.70, 735.51)
50-54	566.58 (559.16, 574.10)	424.91 (419.86, 430.02)	709.09 (698.61, 719.72)
55-59	550.41 (542.65, 558.29)	414.11 (408.80, 419.49)	683.93 (673.06, 694.98)
60-64	531.26 (522.99, 539.65)	401.45 (395.76, 407.23)	654.35 (642.91, 665.99)
65-69	512.47 (502.71, 522.42)	385.59 (378.83, 392.46)	628.08 (614.70, 641.76)
70-74	493.36 (482.46, 504.52)	366.95 (359.37, 374.69)	602.00 (587.23, 617.14)
75-79	475.48 (463.00, 488.31)	348.39 (339.60, 357.40)	575.33 (558.78, 592.37)
80-84	454.44 (439.59, 469.79)	324.71 (314.01, 335.79)	543.36 (524.31, 563.10)
85-89	411.83 (393.11, 431.44)	278.63 (264.66, 293.33)	487.46 (464.43, 511.64)
90-94	351.64 (325.19, 380.25)	218.33 (197.75, 241.06)	412.22 (381.21, 445.75)
95+	277.08 (236.22, 325.00)	154.04 (122.18, 194.20)	323.77 (277.40, 377.89)

Notes: YLDs, years lived with disability; CI, confidence interval.

Source: Institute for Health Metrics and Evaluation. Used with permission. *All rights reserved.*

Table S4. Relative risks for the incidence, prevalence, and years lived with disability of anxiety disorders for each period compared with the reference period (2002–2006) and the corresponding 95% CIs stratified by sex.

Periods	Both	Male	Female
**Incidence**			
1992-1996	1.0027 (0.9870, 1.0186)	1.0178 (1.0008, 1.0350)	0.9933 (0.9768, 1.0102)
1997-2001	1.0103 (0.9993, 1.0215)	1.0140 (1.0027, 1.0255)	1.0085 (0.9966, 1.0206)
2002-2006	1.0000 (1.0000, 1.0000)	1.0000 (1.0000, 1.0000)	1.0000 (1.0000, 1.0000)
2007-2011	1.0076 (0.9968, 1.0185)	1.0104 (0.9993, 1.0217)	1.0048 (0.9930, 1.0166)
2012-2016	0.9974 (0.9819, 1.0132)	1.0104 (0.9937, 1.0275)	0.9866 (0.9702, 1.0032)
2017-2021	1.0679 (1.0452, 1.0912)	1.0879 (1.0629, 1.1136)	1.0514 (1.0277, 1.0756)
**Prevalence**			
1992-1996	0.9915 (0.9818, 1.0012)	1.0153 (1.0054, 1.0254)	0.9758 (0.9653, 0.9864)
1997-2001	1.0086 (0.9996, 1.0177)	1.0123 (1.0039, 1.0209)	1.0055 (0.9955, 1.0156)
2002-2006	1.0000 (1.0000, 1.0000)	1.0000 (1.0000, 1.0000)	1.0000 (1.0000, 1.0000)
2007-2011	0.9972 (0.9886, 1.0059)	1.0108 (1.0026, 1.0191)	0.9906 (0.9811, 1.0003)
2012-2016	0.9750 (0.9658, 0.9843)	1.0073 (0.9977, 1.0171)	0.9593 (0.9494, 0.9693)
2017-2021	1.0366 (1.0256, 1.0476)	1.0796 (1.0670, 1.0923)	1.0173 (1.0057, 1.0289)
**YLDs**			
1992-1996	0.9921 (0.9822, 1.0020)	1.0149 (1.0046, 1.0254)	0.9766 (0.9660, 0.9873)
1997-2001	1.0087 (0.9997, 1.0178)	1.0119 (1.0033, 1.0206)	1.0058 (0.9958, 1.0159)
2002-2006	1.0000 (1.0000, 1.0000)	1.0000 (1.0000, 1.0000)	1.0000 (1.0000, 1.0000)
2007-2011	0.9979 (0.9893, 1.0066)	1.0115 (1.0031, 1.0199)	0.9913 (0.9817, 1.0009)
2012-2016	0.9762 (0.9668, 0.9856)	1.0086 (0.9985, 1.0189)	0.9601 (0.9500, 0.9703)
2017-2021	1.0368 (1.0254, 1.0483)	1.0808 (1.0673, 1.0944)	1.0166 (1.0047, 1.0286)

Notes: YLDs, years lived with disability; CI, confidence interval.
Source: Institute for Health Metrics and Evaluation. Used with permission. *All rights reserved.*

Table S5. Relative risk for anxiety disorders incidence, prevalence, and years lived with disability of each birth cohort compared with the reference (cohort 1957-1961) and the corresponding 95% CIs by sex.

Cohorts	Both	Male	Female
	**Incidence**		
1897-1901	0.96 (0.14, 6.86)	0.93 (0.10, 8.73)	1.00 (0.13, 7.45)
1902-1906	0.96 (0.57, 1.60)	0.94 (0.54, 1.63)	0.99 (0.58, 1.70)
1907-1911	0.96 (0.78, 1.18)	0.94 (0.76, 1.16)	0.98 (0.78, 1.24)
1912-1916	0.96 (0.86, 1.07)	0.95 (0.85, 1.06)	0.98 (0.87, 1.11)
1917-1921	0.97 (0.90, 1.04)	0.96 (0.90, 1.03)	0.99 (0.91, 1.07)
1922-1926	0.98 (0.93, 1.03)	0.98 (0.94, 1.03)	0.99 (0.94, 1.05)
1927-1931	0.99 (0.95, 1.03)	0.98 (0.95, 1.02)	1.00 (0.95, 1.04)
1932-1936	0.99 (0.97, 1.02)	0.99 (0.97, 1.02)	1.00 (0.96, 1.03)
1937-1941	0.99 (0.97, 1.02)	0.99 (0.97, 1.02)	1.00 (0.97, 1.03)
1942-1946	0.99 (0.97, 1.01)	0.99 (0.97, 1.01)	0.99 (0.97, 1.02)
1947-1951	0.99 (0.97, 1.01)	0.99 (0.98, 1.01)	0.99 (0.97, 1.01)
1952-1956	0.99 (0.97, 1.01)	0.99 (0.97, 1.00)	0.99 (0.97, 1.01)
1957-1961	1.00 (1.00, 1.00)	1.00 (1.00, 1.00)	1.00 (1.00, 1.00)
1962-1966	1.01 (1.00, 1.03)	1.01 (1.00, 1.03)	1.01 (1.00, 1.03)
1967-1971	1.02 (1.00, 1.03)	1.01 (1.00, 1.03)	1.02 (1.00, 1.03)
1972-1976	1.03 (1.01, 1.04)	1.03 (1.01, 1.04)	1.03 (1.01, 1.04)
1977-1981	1.05 (1.04, 1.07)	1.06 (1.04, 1.08)	1.04 (1.03, 1.06)
1982-1986	1.07 (1.05, 1.08)	1.08 (1.06, 1.10)	1.06 (1.04, 1.08)
1987-1991	1.07 (1.06, 1.09)	1.09 (1.07, 1.11)	1.06 (1.04, 1.08)
1992-1996	1.09 (1.07, 1.11)	1.11 (1.10, 1.13)	1.08 (1.06, 1.10)
1997-2001	1.11 (1.09, 1.13)	1.14 (1.12, 1.16)	1.10 (1.07, 1.12)
2002-2006	1.12 (1.10, 1.14)	1.14 (1.12, 1.17)	1.11 (1.09, 1.13)
2007-2011	1.13 (1.11, 1.15)	1.15 (1.12, 1.17)	1.12 (1.09, 1.15)
2012-2016	1.16 (1.13, 1.19)	1.17 (1.14, 1.20)	1.15 (1.12, 1.18)
2017-2021	1.22 (1.15, 1.28)	1.22 (1.16, 1.29)	1.21 (1.14, 1.28)
	**Prevalence**		
1897-1901	1.04 (0.63, 1.74)	0.91 (0.42, 2.01)	1.06 (0.65, 1.73)
1902-1906	1.03 (0.84, 1.26)	0.92 (0.70, 1.21)	1.04 (0.86, 1.27)
1907-1911	1.03 (0.93, 1.13)	0.93 (0.82, 1.05)	1.04 (0.94, 1.15)
1912-1916	1.03 (0.97, 1.09)	0.94 (0.88, 1.01)	1.04 (0.97, 1.11)
1917-1921	1.02 (0.97, 1.07)	0.96 (0.92, 1.01)	1.03 (0.98, 1.08)
1922-1926	1.03 (0.99, 1.06)	0.98 (0.94, 1.01)	1.03 (1.00, 1.07)
1927-1931	1.01 (0.98, 1.04)	0.97 (0.95, 1.00)	1.02 (0.99, 1.05)
1932-1936	1.00 (0.98, 1.02)	0.98 (0.96, 1.00)	1.01 (0.99, 1.04)
1937-1941	0.99 (0.97, 1.01)	0.98 (0.96, 1.00)	1.00 (0.98, 1.03)
1942-1946	0.99 (0.97, 1.01)	0.98 (0.97, 1.00)	1.00 (0.98, 1.02)
1947-1951	0.99 (0.98, 1.01)	0.99 (0.97, 1.00)	1.00 (0.98, 1.02)
1952-1956	0.99 (0.97, 1.00)	0.99 (0.97, 1.00)	0.99 (0.98, 1.01)

Cohorts	Both	Male	Female
1957-1961	1.00 (1.00, 1.00)	1.00 (1.00, 1.00)	1.00 (1.00, 1.00)
1962-1966	1.01 (0.99, 1.02)	1.01 (1.00, 1.02)	1.00 (0.99, 1.02)
1967-1971	1.00 (0.99, 1.01)	1.01 (0.99, 1.02)	0.99 (0.98, 1.01)
1972-1976	1.01 (0.99, 1.02)	1.02 (1.01, 1.03)	1.00 (0.98, 1.01)
1977-1981	1.03 (1.01, 1.04)	1.05 (1.03, 1.06)	1.01 (1.00, 1.03)
1982-1986	1.05 (1.03, 1.06)	1.07 (1.05, 1.08)	1.03 (1.01, 1.05)
1987-1991	1.05 (1.04, 1.07)	1.08 (1.06, 1.09)	1.03 (1.02, 1.05)
1992-1996	1.06 (1.05, 1.08)	1.09 (1.08, 1.11)	1.05 (1.03, 1.07)
1997-2001	1.07 (1.05, 1.09)	1.10 (1.08, 1.12)	1.06 (1.03, 1.08)
2002-2006	1.08 (1.05, 1.10)	1.10 (1.08, 1.12)	1.06 (1.04, 1.09)
2007-2011	1.09 (1.06, 1.12)	1.11 (1.08, 1.13)	1.08 (1.05, 1.11)
2012-2016	1.12 (1.08, 1.17)	1.14 (1.10, 1.17)	1.11 (1.07, 1.16)
2017-2021	1.18 (1.04, 1.35)	1.19 (1.06, 1.33)	1.17 (1.01, 1.37)
**YLDs**			
1897-1901	1.05 (0.60, 1.85)	0.91 (0.38, 2.19)	1.07 (0.62, 1.83)
1902-1906	1.03 (0.83, 1.28)	0.92 (0.68, 1.24)	1.05 (0.85, 1.30)
1907-1911	1.03 (0.92, 1.14)	0.93 (0.81, 1.06)	1.04 (0.94, 1.16)
1912-1916	1.03 (0.96, 1.10)	0.94 (0.87, 1.01)	1.04 (0.97, 1.11)
1917-1921	1.02 (0.97, 1.07)	0.96 (0.91, 1.01)	1.03 (0.98, 1.08)
1922-1926	1.03 (0.99, 1.06)	0.97 (0.94, 1.01)	1.04 (1.00, 1.08)
1927-1931	1.01 (0.98, 1.04)	0.97 (0.95, 1.00)	1.03 (0.99, 1.06)
1932-1936	1.00 (0.98, 1.02)	0.98 (0.95, 1.00)	1.01 (0.99, 1.04)
1937-1941	0.99 (0.97, 1.01)	0.98 (0.96, 1.00)	1.00 (0.98, 1.03)
1942-1946	0.99 (0.97, 1.01)	0.98 (0.97, 1.00)	1.00 (0.98, 1.02)
1947-1951	0.99 (0.98, 1.01)	0.99 (0.97, 1.00)	1.00 (0.98, 1.02)
1952-1956	0.99 (0.97, 1.00)	0.99 (0.97, 1.00)	0.99 (0.98, 1.01)
1957-1961	1.00 (1.00, 1.00)	1.00 (1.00, 1.00)	1.00 (1.00, 1.00)
1962-1966	1.01 (0.99, 1.02)	1.01 (1.00, 1.02)	1.00 (0.99, 1.02)
1967-1971	1.00 (0.99, 1.01)	1.01 (1.00, 1.02)	0.99 (0.98, 1.01)
1972-1976	1.01 (0.99, 1.02)	1.02 (1.01, 1.03)	1.00 (0.98, 1.01)
1977-1981	1.03 (1.02, 1.05)	1.05 (1.04, 1.06)	1.01 (1.00, 1.03)
1982-1986	1.05 (1.03, 1.06)	1.07 (1.05, 1.08)	1.03 (1.01, 1.05)
1987-1991	1.05 (1.04, 1.07)	1.08 (1.07, 1.10)	1.04 (1.02, 1.05)
1992-1996	1.07 (1.05, 1.08)	1.10 (1.08, 1.11)	1.05 (1.03, 1.07)
1997-2001	1.07 (1.05, 1.09)	1.10 (1.08, 1.12)	1.05 (1.03, 1.08)
2002-2006	1.08 (1.06, 1.10)	1.10 (1.08, 1.12)	1.06 (1.04, 1.09)
2007-2011	1.09 (1.07, 1.12)	1.11 (1.09, 1.14)	1.08 (1.05, 1.11)
2012-2016	1.13 (1.09, 1.17)	1.14 (1.11, 1.18)	1.12 (1.07, 1.16)
2017-2021	1.19 (1.05, 1.35)	1.20 (1.07, 1.34)	1.18 (1.02, 1.37)

Notes: YLDs, years lived with disability; CI, confidence interval.

Source: Institute for Health Metrics and Evaluation. Used with permission. *All rights reserved.*

Table S6. Fitted longitudinal age effects of incidence, prevalence, and years lived with disability of anxiety disorders (per 100,000 person-years) and the corresponding 95% CIs by SDI region.

Age group (years)	Total population				
	Low SDI	Low-middle SDI	Middle SDI	Middle-high SDI	High SDI
Incidence					
0-4	62.21 (61.06, 63.38)	55.67 (53.81, 57.60)	90.03 (87.17, 92.98)	111.96 (107.20, 116.94)	113.19 (108.27, 118.35)
5-9	397.39 (391.73, 403.13)	349.02 (341.02, 357.21)	547.60 (536.04, 559.40)	675.87 (657.41, 694.84)	690.29 (671.86, 709.23)
10-14	583.95 (575.99, 592.01)	521.50 (510.35, 532.91)	729.54 (715.17, 744.20)	866.14 (844.29, 888.55)	961.71 (938.31, 985.68)
15-19	610.05 (601.88, 618.32)	552.34 (540.85, 564.08)	619.09 (607.11, 631.31)	651.92 (635.77, 668.48)	899.52 (878.45, 921.08)
20-24	628.28 (620.00, 636.68)	585.39 (573.51, 597.51)	583.52 (572.49, 594.77)	551.83 (538.52, 565.47)	849.51 (830.43, 869.03)
25-29	636.06 (627.79, 644.44)	618.93 (606.68, 631.44)	612.53 (601.37, 623.90)	568.85 (555.79, 582.22)	839.48 (821.50, 857.84)
30-34	643.49 (635.23, 651.86)	651.53 (638.93, 664.37)	642.62 (631.29, 654.15)	585.79 (572.93, 598.93)	831.44 (814.48, 848.75)
35-39	651.52 (643.76, 659.37)	685.92 (673.75, 698.30)	675.94 (665.09, 686.97)	600.83 (589.02, 612.88)	824.34 (809.40, 839.56)
40-44	630.68 (623.14, 638.31)	682.68 (670.65, 694.92)	673.47 (662.82, 684.29)	592.73 (581.34, 604.35)	780.94 (767.16, 794.97)
45-49	581.72 (574.45, 589.07)	640.36 (628.74, 652.20)	635.15 (624.89, 645.58)	563.94 (552.97, 575.13)	702.17 (689.53, 715.03)
50-54	531.86 (524.64, 539.18)	595.34 (583.74, 607.17)	594.00 (583.86, 604.31)	534.81 (523.97, 545.88)	623.71 (611.84, 635.81)
55-59	480.35 (472.99, 487.83)	545.28 (533.44, 557.38)	550.28 (539.90, 560.86)	504.73 (493.65, 516.07)	542.91 (531.52, 554.54)
60-64	429.36 (421.72, 437.14)	487.49 (475.36, 499.93)	498.37 (487.68, 509.29)	464.28 (452.92, 475.92)	475.83 (464.58, 487.34)
65-69	377.88 (369.32, 386.64)	424.06 (410.63, 437.94)	442.33 (430.31, 454.69)	415.43 (402.82, 428.43)	420.28 (408.09, 432.84)
70-74	325.08 (315.82, 334.62)	359.56 (345.35, 374.36)	385.07 (372.25, 398.34)	364.49 (351.31, 378.17)	361.22 (348.84, 374.03)
75-79	271.96 (261.60, 282.72)	294.14 (278.77, 310.35)	328.00 (314.05, 342.56)	311.12 (297.26, 325.63)	297.96 (285.47, 310.99)
80-84	215.17 (202.95, 228.12)	228.91 (211.52, 247.74)	258.75 (243.26, 275.23)	245.54 (230.86, 261.15)	230.08 (217.62, 243.25)
85-89	154.68 (139.15, 171.95)	164.40 (143.37, 188.51)	180.78 (162.56, 201.03)	172.69 (156.18, 190.96)	160.94 (148.21, 174.75)
90-94	93.69 (73.11, 120.05)	98.91 (73.38, 133.33)	106.20 (83.66, 134.80)	102.27 (82.27, 127.11)	94.03 (80.39, 109.98)
95+	30.90 (12.51, 76.29)	33.06 (12.89, 84.76)	34.84 (15.54, 78.10)	33.54 (15.54, 72.39)	30.57 (19.13, 48.84)

Age group (years)	Total population				
	Low SDI	Low-middle SDI	Middle SDI	Middle-high SDI	High SDI
**Prevalence**					
0-4	59.49 (57.54, 61.50)	52.57 (47.84, 57.78)	88.07 (81.78, 94.83)	109.34 (99.36, 120.33)	112.83 (102.30, 124.45)
5-9	942.03 (928.14, 956.14)	817.50 (788.75, 847.29)	1321.90 (1287.25, 1357.47)	1620.88 (1568.25, 1675.27)	1691.96 (1638.39, 1747.28)
10-14	2513.32 (2481.08, 2545.98)	2213.25 (2149.67, 2278.72)	3383.70 (3312.20, 3456.75)	4071.92 (3968.05, 4178.51)	4361.83 (4257.29, 4468.94)
15-19	3535.77 (3492.56, 3579.52)	3162.56 (3077.82, 3249.64)	4239.76 (4155.87, 4325.34)	4782.76 (4669.77, 4898.47)	5768.45 (5644.00, 5895.64)
20-24	4020.72 (3972.91, 4069.10)	3662.81 (3568.48, 3759.63)	4271.65 (4190.13, 4354.75)	4353.16 (4254.39, 4454.22)	6133.93 (6010.22, 6260.18)
25-29	4239.91 (4190.50, 4289.90)	3994.16 (3894.55, 4096.33)	4288.52 (4209.58, 4368.95)	4098.47 (4009.21, 4189.72)	6271.14 (6152.04, 6392.55)
30-34	4324.09 (4274.50, 4374.26)	4249.33 (4146.35, 4354.88)	4383.09 (4304.98, 4462.61)	4044.49 (3959.97, 4130.81)	6327.96 (6214.49, 6443.50)
35-39	4359.98 (4313.53, 4406.93)	4487.84 (4387.89, 4590.07)	4555.03 (4480.77, 4630.52)	4091.33 (4014.50, 4169.64)	6350.44 (6249.46, 6453.06)
40-44	4360.54 (4314.19, 4407.39)	4698.65 (4595.84, 4803.77)	4734.45 (4659.19, 4810.93)	4163.11 (4087.18, 4240.45)	6325.04 (6228.89, 6422.67)
45-49	4285.60 (4238.73, 4332.98)	4786.28 (4680.69, 4894.26)	4827.60 (4750.87, 4905.57)	4211.20 (4134.83, 4288.99)	6154.12 (6060.93, 6248.73)
50-54	4160.67 (4112.19, 4209.72)	4781.56 (4671.06, 4894.67)	4845.62 (4766.15, 4926.42)	4242.86 (4164.33, 4322.86)	5888.85 (5796.68, 5982.49)
55-59	4010.84 (3959.48, 4062.87)	4710.77 (4592.70, 4831.88)	4825.57 (4740.30, 4912.37)	4280.05 (4196.43, 4365.33)	5559.96 (5467.08, 5654.43)
60-64	3861.43 (3805.72, 3917.95)	4597.17 (4469.39, 4728.61)	4775.50 (4682.58, 4870.27)	4318.72 (4227.75, 4411.66)	5245.58 (5150.06, 5342.87)
65-69	3718.17 (3651.57, 3785.98)	4456.68 (4303.44, 4615.37)	4726.12 (4612.66, 4842.37)	4356.49 (4246.19, 4469.65)	5024.29 (4914.35, 5136.68)
70-74	3568.83 (3492.04, 3647.30)	4298.75 (4123.72, 4481.21)	4651.52 (4521.19, 4785.61)	4381.12 (4255.64, 4510.29)	4858.14 (4738.17, 4981.14)
75-79	3423.66 (3331.25, 3518.64)	4126.06 (3919.42, 4343.58)	4586.59 (4432.81, 4745.71)	4411.59 (4266.35, 4561.77)	4705.84 (4572.44, 4843.14)
80-84	3270.19 (3150.12, 3394.82)	3933.97 (3674.68, 4211.57)	4498.45 (4307.08, 4698.33)	4420.60 (4245.77, 4602.62)	4510.29 (4359.74, 4666.04)
85-89	2993.08 (2820.89, 3175.78)	3614.59 (3260.98, 4006.55)	4179.25 (3921.81, 4453.58)	4212.97 (3986.94, 4451.82)	4118.19 (3942.84, 4301.34)
90-94	2602.49 (2321.89, 2917.01)	3173.00 (2641.10, 3812.01)	3642.18 (3250.13, 4081.53)	3790.74 (3454.97, 4159.15)	3551.63 (3325.91, 3792.68)
95+	2151.35 (1655.18, 2796.25)	2741.84 (1910.93, 3934.06)	2882.83 (2256.29, 3683.37)	3143.82 (2571.46, 3843.57)	2840.88 (2515.76, 3208.01)
**YLDs**					

Age group (years)	Total population				
	**Low SDI**	**Low-middle SDI**	**Middle SDI**	**Middle-high SDI**	**High SDI**
0-4	7.21 (6.99, 7.44)	6.49 (5.93, 7.11)	11.01 (10.24, 11.83)	13.70 (12.46, 15.05)	14.23 (12.96, 15.63)
5-9	114.69 (113.07, 116.33)	100.93 (97.47, 104.52)	165.49 (161.20, 169.89)	203.10 (196.53, 209.89)	213.26 (206.73, 220.00)
10-14	304.72 (300.95, 308.53)	271.68 (264.01, 279.57)	420.73 (411.88, 429.77)	506.11 (493.15, 519.40)	544.08 (531.36, 557.11)
15-19	426.20 (421.18, 431.29)	385.14 (374.98, 395.58)	522.34 (512.03, 532.87)	588.74 (574.73, 603.08)	708.23 (693.25, 723.53)
20-24	481.48 (475.95, 487.07)	442.78 (431.52, 454.32)	521.62 (511.66, 531.77)	531.40 (519.24, 543.84)	745.28 (730.51, 760.36)
25-29	503.84 (498.16, 509.59)	478.96 (467.15, 491.06)	519.24 (509.67, 529.00)	496.41 (485.49, 507.58)	755.92 (741.78, 770.33)
30-34	510.99 (505.32, 516.73)	506.06 (493.92, 518.49)	527.06 (517.64, 536.65)	486.99 (476.69, 497.51)	758.33 (744.92, 771.99)
35-39	512.40 (507.11, 517.74)	530.73 (519.02, 542.70)	543.87 (534.96, 552.92)	489.56 (480.24, 499.05)	755.83 (743.97, 767.89)
40-44	509.56 (504.30, 514.87)	551.49 (539.51, 563.74)	561.03 (552.05, 570.14)	494.78 (485.62, 504.12)	747.12 (735.88, 758.52)
45-49	497.93 (492.63, 503.28)	557.42 (545.19, 569.93)	567.76 (558.66, 577.01)	496.57 (487.41, 505.91)	721.52 (710.69, 732.52)
50-54	479.85 (474.40, 485.36)	552.00 (539.27, 565.02)	565.14 (555.77, 574.66)	496.27 (486.89, 505.82)	685.27 (674.60, 696.10)
55-59	458.03 (452.29, 463.84)	537.80 (524.30, 551.66)	556.49 (546.51, 566.66)	494.98 (485.07, 505.09)	640.05 (629.38, 650.90)
60-64	435.84 (429.65, 442.11)	517.86 (503.36, 532.78)	543.09 (532.31, 554.10)	492.76 (482.07, 503.69)	595.30 (584.42, 606.37)
65-69	412.15 (404.84, 419.58)	492.78 (475.62, 510.56)	527.53 (514.55, 540.84)	488.76 (475.96, 501.90)	559.97 (547.63, 572.59)
70-74	387.26 (378.94, 395.76)	465.31 (445.96, 485.50)	508.00 (493.29, 523.14)	482.09 (467.72, 496.91)	530.22 (516.94, 543.83)
75-79	362.34 (352.46, 372.49)	435.99 (413.44, 459.77)	489.22 (472.11, 506.96)	475.27 (458.85, 492.29)	502.12 (487.57, 517.10)
80-84	337.70 (325.00, 350.89)	405.90 (377.92, 435.95)	467.66 (446.64, 489.66)	465.37 (445.85, 485.75)	469.31 (453.14, 486.06)
85-89	302.39 (284.31, 321.61)	363.72 (325.96, 405.85)	422.85 (394.91, 452.77)	432.15 (407.18, 458.65)	415.61 (397.06, 435.02)
90-94	257.19 (227.99, 290.13)	312.06 (255.91, 380.53)	358.63 (316.59, 406.25)	378.79 (342.06, 419.45)	347.89 (324.29, 373.20)
95+	206.41 (155.73, 273.59)	263.35 (177.26, 391.24)	275.68 (209.84, 362.19)	305.03 (243.54, 382.04)	270.12 (236.59, 308.41)

Notes: YLDs, years lived with disability; CI, confidence interval; SDI, socio-demographic index.
Source: Institute for Health Metrics and Evaluation. Used with permission. All rights reserved.

Table S7. Relative risk for anxiety disorders incidence, prevalence, and years lived with disability of each period compared with the reference (2002–2006) and the corresponding 95% CIs by SDI region.

Periods	Total population				
	Low SDI	Low-middle SDI	Middle SDI	Middle-high SDI	High SDI
**Incidence**					
1992-1996	1.0110 (0.9897, 1.0327)	1.0088 (0.9835, 1.0347)	0.9982 (0.9751, 1.0219)	1.0131 (0.9903, 1.0365)	0.9564 (0.9405, 0.9726)
1997-2001	1.0028 (0.9907, 1.0150)	0.9894 (0.9738, 1.0052)	1.0136 (0.9989, 1.0286)	1.0226 (1.0063, 1.0391)	1.0017 (0.9881, 1.0155)
2002-2006	1.0000 (1.0000, 1.0000)	1.0000 (1.0000, 1.0000)	1.0000 (1.0000, 1.0000)	1.0000 (1.0000, 1.0000)	1.0000 (1.0000, 1.0000)
2007-2011	1.0143 (1.0023, 1.0264)	1.0255 (1.0097, 1.0416)	1.0035 (0.9890, 1.0181)	1.0055 (0.9895, 1.0218)	0.9972 (0.9838, 1.0108)
2012-2016	1.0165 (0.9952, 1.0383)	1.0197 (0.9943, 1.0457)	0.9930 (0.9700, 1.0165)	0.9997 (0.9770, 1.0228)	0.9599 (0.9439, 0.9763)
2017-2021	1.0658 (1.0332, 1.0995)	1.0801 (1.0420, 1.1197)	1.0669 (1.0320, 1.1030)	1.0654 (1.0328, 1.0990)	1.0358 (1.0140, 1.0580)
**Prevalence**					
1992-1996	1.0087 (0.9990, 1.0185)	0.9824 (0.9641, 1.0010)	0.9754 (0.9623, 0.9886)	1.0125 (0.9984, 1.0268)	0.9541 (0.9427, 0.9656)
1997-2001	1.0024 (0.9949, 1.0099)	0.9751 (0.9596, 0.9908)	1.0008 (0.9894, 1.0124)	1.0194 (1.0062, 1.0328)	1.0196 (1.0080, 1.0312)
2002-2006	1.0000 (1.0000, 1.0000)	1.0000 (1.0000, 1.0000)	1.0000 (1.0000, 1.0000)	1.0000 (1.0000, 1.0000)	1.0000 (1.0000, 1.0000)
2007-2011	1.0122 (1.0050, 1.0194)	1.0251 (1.0096, 1.0408)	0.9994 (0.9883, 1.0106)	1.0013 (0.9886, 1.0143)	0.9744 (0.9635, 0.9855)
2012-2016	1.0168 (1.0073, 1.0263)	1.0144 (0.9963, 1.0328)	0.9749 (0.9622, 0.9878)	0.9853 (0.9717, 0.9991)	0.9360 (0.9250, 0.9471)
2017-2021	1.0645 (1.0515, 1.0777)	1.0700 (1.0467, 1.0938)	1.0410 (1.0247, 1.0574)	1.0397 (1.0239, 1.0558)	1.0154 (1.0030, 1.0280)
**YLDs**					
1992-1996	1.0069 (0.9970, 1.0168)	0.9826 (0.9639, 1.0017)	0.9763 (0.9627, 0.9900)	1.0114 (0.9969, 1.0261)	0.9546 (0.9433, 0.9661)
1997-2001	1.0009 (0.9936, 1.0083)	0.9748 (0.9594, 0.9905)	1.0014 (0.9898, 1.0132)	1.0189 (1.0055, 1.0325)	1.0193 (1.0079, 1.0309)
2002-2006	1.0000 (1.0000, 1.0000)	1.0000 (1.0000, 1.0000)	1.0000 (1.0000, 1.0000)	1.0000 (1.0000, 1.0000)	1.0000 (1.0000, 1.0000)
2007-2011	1.0153 (1.0082, 1.0224)	1.0275 (1.0120, 1.0432)	1.0000 (0.9887, 1.0113)	1.0020 (0.9889, 1.0151)	0.9738 (0.9630, 0.9848)
2012-2016	1.0218 (1.0121, 1.0316)	1.0180 (0.9993, 1.0369)	0.9759 (0.9628, 0.9893)	0.9868 (0.9727, 1.0010)	0.9348 (0.9239, 0.9459)
2017-2021	1.0700 (1.0564, 1.0838)	1.0737 (1.0492, 1.0987)	1.0411 (1.0239, 1.0585)	1.0407 (1.0241, 1.0575)	1.0121 (0.9996, 1.0248)

Notes: YLDs, years lived with disability; SDI, socio-demographic index. Source: Institute for Health Metrics and Evaluation. Used with permission. *All rights reserved.*

Table S8. Relative risk for anxiety disorders incidence, prevalence, and years lived with disability of each birth cohort compared with the reference (cohort 1957-1961) and the corresponding 95% CIs by SDI region.

Cohorts	Total population				
	Low SDI	Low-middle SDI	Middle SDI	Middle-high SDI	High SDI
			Incidence		
1897-1901	0.92 (0.04, 20.83)	0.96 (0.03, 29.63)	0.98 (0.04, 25.65)	0.94 (0.05, 17.39)	1.01 (0.20, 5.22)
1902-1906	0.94 (0.44, 2.04)	0.96 (0.39, 2.36)	0.98 (0.44, 2.20)	0.94 (0.47, 1.88)	0.99 (0.63, 1.57)
1907-1911	0.96 (0.72, 1.28)	0.96 (0.67, 1.37)	0.99 (0.73, 1.35)	0.94 (0.72, 1.23)	0.98 (0.80, 1.19)
1912-1916	0.96 (0.83, 1.11)	0.96 (0.80, 1.16)	1.00 (0.85, 1.16)	0.95 (0.83, 1.09)	0.96 (0.86, 1.08)
1917-1921	0.97 (0.89, 1.05)	0.97 (0.86, 1.09)	1.01 (0.92, 1.10)	0.98 (0.90, 1.08)	0.96 (0.88, 1.03)
1922-1926	0.97 (0.92, 1.03)	0.97 (0.90, 1.05)	1.02 (0.96, 1.08)	1.00 (0.94, 1.06)	0.96 (0.91, 1.01)
1927-1931	0.98 (0.94, 1.02)	0.98 (0.93, 1.04)	1.02 (0.98, 1.07)	1.00 (0.96, 1.05)	0.96 (0.91, 1.00)
1932-1936	0.99 (0.96, 1.02)	0.99 (0.95, 1.03)	1.03 (0.99, 1.06)	1.01 (0.98, 1.05)	0.95 (0.92, 0.99)
1937-1941	1.00 (0.97, 1.02)	0.99 (0.96, 1.03)	1.02 (0.99, 1.05)	1.01 (0.98, 1.05)	0.95 (0.92, 0.98)
1942-1946	1.00 (0.98, 1.02)	0.99 (0.96, 1.02)	1.01 (0.98, 1.04)	1.01 (0.98, 1.04)	0.95 (0.92, 0.98)
1947-1951	1.00 (0.98, 1.02)	0.99 (0.97, 1.02)	1.00 (0.98, 1.02)	1.01 (0.98, 1.04)	0.96 (0.94, 0.98)
1952-1956	1.00 (0.98, 1.01)	0.99 (0.97, 1.01)	0.99 (0.97, 1.01)	1.00 (0.98, 1.02)	0.98 (0.96, 1.00)
1957-1961	1.00 (1.00, 1.00)	1.00 (1.00, 1.00)	1.00 (1.00, 1.00)	1.00 (1.00, 1.00)	1.00 (1.00, 1.00)
1962-1966	1.01 (1.00, 1.02)	1.02 (1.00, 1.04)	1.01 (1.00, 1.03)	1.01 (0.99, 1.03)	1.01 (1.00, 1.03)
1967-1971	1.02 (1.01, 1.04)	1.04 (1.02, 1.06)	1.02 (1.00, 1.03)	1.01 (0.99, 1.04)	1.01 (0.99, 1.03)
1972-1976	1.04 (1.03, 1.05)	1.06 (1.04, 1.09)	1.04 (1.02, 1.06)	1.02 (1.00, 1.05)	1.00 (0.98, 1.02)
1977-1981	1.05 (1.04, 1.07)	1.09 (1.07, 1.11)	1.08 (1.06, 1.10)	1.04 (1.01, 1.06)	1.00 (0.97, 1.02)
1982-1986	1.06 (1.05, 1.07)	1.11 (1.09, 1.14)	1.10 (1.08, 1.12)	1.02 (1.00, 1.05)	1.01 (0.99, 1.03)
1987-1991	1.07 (1.05, 1.08)	1.13 (1.10, 1.15)	1.11 (1.09, 1.13)	1.01 (0.99, 1.04)	1.03 (1.00, 1.05)
1992-1996	1.08 (1.07, 1.10)	1.14 (1.12, 1.17)	1.13 (1.11, 1.16)	1.05 (1.02, 1.07)	1.05 (1.03, 1.08)

Cohorts	Total population				
	Low SDI	Low-middle SDI	Middle SDI	Middle-high SDI	High SDI
1997-2001	1.11 (1.09, 1.12)	1.17 (1.14, 1.20)	1.16 (1.13, 1.18)	1.09 (1.06, 1.12)	1.09 (1.06, 1.12)
2002-2006	1.14 (1.12, 1.16)	1.22 (1.19, 1.25)	1.16 (1.13, 1.18)	1.11 (1.08, 1.14)	1.13 (1.10, 1.16)
2007-2011	1.18 (1.16, 1.19)	1.28 (1.24, 1.31)	1.15 (1.13, 1.18)	1.09 (1.06, 1.13)	1.15 (1.11, 1.18)
2012-2016	1.22 (1.20, 1.24)	1.35 (1.31, 1.39)	1.17 (1.13, 1.20)	1.09 (1.05, 1.14)	1.18 (1.13, 1.23)
2017-2021	1.29 (1.25, 1.33)	1.44 (1.35, 1.54)	1.23 (1.15, 1.31)	1.15 (1.05, 1.25)	1.24 (1.13, 1.36)
**Prevalence**					
1897-1901	0.95 (0.40, 2.28)	0.85 (0.22, 3.24)	0.94 (0.35, 2.56)	0.98 (0.46, 2.08)	1.17 (0.79, 1.75)
1902-1906	0.95 (0.68, 1.32)	0.87 (0.52, 1.47)	0.95 (0.66, 1.37)	0.99 (0.75, 1.29)	1.12 (0.95, 1.33)
1907-1911	0.95 (0.81, 1.10)	0.89 (0.69, 1.14)	0.98 (0.83, 1.16)	1.01 (0.89, 1.15)	1.09 (1.00, 1.19)
1912-1916	0.96 (0.88, 1.04)	0.92 (0.79, 1.07)	1.01 (0.92, 1.12)	1.04 (0.96, 1.12)	1.06 (1.00, 1.12)
1917-1921	0.97 (0.92, 1.02)	0.94 (0.85, 1.04)	1.03 (0.97, 1.10)	1.06 (1.00, 1.12)	1.01 (0.97, 1.06)
1922-1926	0.97 (0.94, 1.01)	0.95 (0.89, 1.03)	1.03 (0.98, 1.08)	1.06 (1.02, 1.11)	1.02 (0.98, 1.05)
1927-1931	0.98 (0.95, 1.00)	0.96 (0.91, 1.02)	1.02 (0.98, 1.06)	1.04 (1.01, 1.08)	0.99 (0.96, 1.02)
1932-1936	0.98 (0.96, 1.01)	0.97 (0.93, 1.01)	1.01 (0.98, 1.04)	1.04 (1.01, 1.07)	0.97 (0.94, 1.00)
1937-1941	0.99 (0.97, 1.01)	0.98 (0.94, 1.01)	1.00 (0.97, 1.02)	1.02 (1.00, 1.05)	0.97 (0.95, 0.99)
1942-1946	0.99 (0.98, 1.01)	0.98 (0.94, 1.01)	0.99 (0.96, 1.01)	1.02 (1.00, 1.05)	0.97 (0.95, 0.99)
1947-1951	0.99 (0.98, 1.01)	0.98 (0.95, 1.01)	0.98 (0.96, 1.01)	1.02 (0.99, 1.04)	0.98 (0.96, 0.99)
1952-1956	1.00 (0.98, 1.01)	0.98 (0.96, 1.01)	0.98 (0.96, 1.00)	1.00 (0.98, 1.02)	0.99 (0.97, 1.00)
1957-1961	1.00 (1.00, 1.00)	1.00 (1.00, 1.00)	1.00 (1.00, 1.00)	1.00 (1.00, 1.00)	1.00 (1.00, 1.00)
1962-1966	1.01 (1.00, 1.02)	1.02 (1.00, 1.04)	1.01 (0.99, 1.03)	1.01 (0.99, 1.02)	1.00 (0.98, 1.02)
1967-1971	1.02 (1.01, 1.03)	1.04 (1.02, 1.06)	1.01 (0.99, 1.03)	1.00 (0.98, 1.02)	0.98 (0.97, 1.00)
1972-1976	1.03 (1.02, 1.04)	1.06 (1.04, 1.09)	1.03 (1.01, 1.05)	1.01 (0.99, 1.03)	0.96 (0.95, 0.98)
1977-1981	1.04 (1.03, 1.06)	1.09 (1.07, 1.12)	1.07 (1.05, 1.09)	1.02 (1.00, 1.04)	0.96 (0.94, 0.98)

Cohorts	Total population				
	Low SDI	Low-middle SDI	Middle SDI	Middle-high SDI	High SDI
1982-1986	1.05 (1.04, 1.06)	1.11 (1.09, 1.14)	1.10 (1.08, 1.12)	1.02 (1.00, 1.04)	0.97 (0.95, 0.99)
1987-1991	1.07 (1.05, 1.08)	1.13 (1.10, 1.16)	1.11 (1.09, 1.13)	1.02 (0.99, 1.04)	0.99 (0.97, 1.01)
1992-1996	1.09 (1.07, 1.10)	1.15 (1.12, 1.18)	1.12 (1.09, 1.14)	1.04 (1.01, 1.06)	1.03 (1.00, 1.05)
1997-2001	1.11 (1.10, 1.13)	1.19 (1.15, 1.22)	1.12 (1.10, 1.15)	1.06 (1.03, 1.09)	1.06 (1.04, 1.09)
2002-2006	1.14 (1.13, 1.16)	1.23 (1.19, 1.27)	1.12 (1.09, 1.15)	1.08 (1.04, 1.11)	1.09 (1.05, 1.12)
2007-2011	1.18 (1.16, 1.20)	1.29 (1.24, 1.35)	1.13 (1.09, 1.16)	1.07 (1.03, 1.11)	1.10 (1.06, 1.14)
2012-2016	1.22 (1.20, 1.25)	1.38 (1.30, 1.46)	1.15 (1.10, 1.21)	1.08 (1.01, 1.14)	1.13 (1.06, 1.20)
2017-2021	1.29 (1.21, 1.38)	1.48 (1.21, 1.81)	1.21 (1.03, 1.43)	1.13 (0.91, 1.40)	1.19 (0.95, 1.48)
YLDs					
1897-1901	0.93 (0.36, 2.39)	0.85 (0.19, 3.67)	0.94 (0.31, 2.88)	0.97 (0.42, 2.28)	1.19 (0.77, 1.84)
1902-1906	0.93 (0.65, 1.32)	0.87 (0.49, 1.53)	0.96 (0.64, 1.42)	0.99 (0.73, 1.33)	1.14 (0.95, 1.36)
1907-1911	0.93 (0.79, 1.09)	0.88 (0.67, 1.16)	0.99 (0.82, 1.18)	1.01 (0.88, 1.16)	1.10 (1.00, 1.21)
1912-1916	0.94 (0.86, 1.03)	0.91 (0.78, 1.07)	1.02 (0.92, 1.13)	1.03 (0.95, 1.12)	1.07 (1.00, 1.13)
1917-1921	0.95 (0.90, 1.01)	0.93 (0.84, 1.04)	1.03 (0.96, 1.11)	1.05 (0.99, 1.12)	1.02 (0.97, 1.07)
1922-1926	0.96 (0.93, 1.00)	0.95 (0.88, 1.02)	1.03 (0.98, 1.08)	1.06 (1.01, 1.11)	1.02 (0.99, 1.06)
1927-1931	0.97 (0.94, 1.00)	0.96 (0.90, 1.01)	1.02 (0.98, 1.06)	1.04 (1.00, 1.08)	1.00 (0.96, 1.03)
1932-1936	0.98 (0.95, 1.00)	0.97 (0.92, 1.01)	1.01 (0.98, 1.04)	1.03 (1.00, 1.07)	0.97 (0.95, 1.00)
1937-1941	0.99 (0.97, 1.01)	0.97 (0.94, 1.01)	1.00 (0.97, 1.03)	1.02 (0.99, 1.05)	0.97 (0.95, 1.00)
1942-1946	0.99 (0.97, 1.01)	0.97 (0.94, 1.01)	0.99 (0.97, 1.01)	1.02 (0.99, 1.05)	0.97 (0.95, 0.99)
1947-1951	0.99 (0.98, 1.01)	0.98 (0.95, 1.00)	0.99 (0.96, 1.01)	1.02 (0.99, 1.04)	0.98 (0.96, 0.99)
1952-1956	1.00 (0.98, 1.01)	0.98 (0.96, 1.01)	0.98 (0.96, 1.00)	1.00 (0.98, 1.02)	0.99 (0.97, 1.00)
1957-1961	1.00 (1.00, 1.00)	1.00 (1.00, 1.00)	1.00 (1.00, 1.00)	1.00 (1.00, 1.00)	1.00 (1.00, 1.00)
1962-1966	1.01 (1.00, 1.02)	1.02 (1.00, 1.04)	1.01 (0.99, 1.03)	1.01 (0.99, 1.03)	1.00 (0.98, 1.02)

Cohorts	Total population				
	Low SDI	Low-middle SDI	Middle SDI	Middle-high SDI	High SDI
1967-1971	1.02 (1.01, 1.03)	1.04 (1.02, 1.07)	1.01 (0.99, 1.03)	1.00 (0.98, 1.02)	0.98 (0.97, 1.00)
1972-1976	1.04 (1.03, 1.05)	1.06 (1.04, 1.09)	1.03 (1.01, 1.05)	1.01 (0.99, 1.03)	0.96 (0.94, 0.98)
1977-1981	1.05 (1.04, 1.06)	1.09 (1.07, 1.12)	1.07 (1.06, 1.09)	1.02 (1.00, 1.05)	0.96 (0.94, 0.98)
1982-1986	1.06 (1.05, 1.07)	1.12 (1.09, 1.15)	1.10 (1.08, 1.12)	1.02 (1.00, 1.04)	0.97 (0.95, 0.99)
1987-1991	1.08 (1.06, 1.09)	1.13 (1.11, 1.16)	1.11 (1.09, 1.13)	1.02 (1.00, 1.05)	0.99 (0.97, 1.01)
1992-1996	1.10 (1.09, 1.11)	1.16 (1.13, 1.19)	1.12 (1.09, 1.14)	1.04 (1.02, 1.07)	1.02 (1.00, 1.05)
1997-2001	1.13 (1.11, 1.14)	1.19 (1.16, 1.23)	1.12 (1.10, 1.15)	1.07 (1.04, 1.10)	1.06 (1.03, 1.08)
2002-2006	1.16 (1.14, 1.18)	1.24 (1.20, 1.28)	1.12 (1.09, 1.15)	1.08 (1.05, 1.11)	1.08 (1.05, 1.11)
2007-2011	1.20 (1.18, 1.22)	1.30 (1.26, 1.36)	1.13 (1.10, 1.16)	1.08 (1.04, 1.12)	1.09 (1.06, 1.13)
2012-2016	1.25 (1.22, 1.27)	1.39 (1.31, 1.47)	1.16 (1.10, 1.21)	1.08 (1.02, 1.15)	1.13 (1.06, 1.19)
2017-2021	1.32 (1.24, 1.40)	1.50 (1.23, 1.82)	1.22 (1.03, 1.43)	1.14 (0.92, 1.41)	1.18 (0.96, 1.47)

Notes: YLDs, years lived with disability; CI, confidence interval; SDI, socio-demographic index.
Source: Institute for Health Metrics and Evaluation. Used with permission. *All rights reserved.*

Table S9. Fitted longitudinal age effects of incidence, prevalence, and years lived with disability of anxiety disorders (per 100,000 person-years) and the corresponding 95% CIs by WHO region.

Age group (years)	Total population					
	AFR	EMR	EUR	AMR	SEAR	WPR
**Incidence**						
0-4	73.24 (71.60, 74.91)	106.36 (103.41, 109.38)	110.32 (107.25, 113.48)	90.46 (85.63, 95.56)	40.18 (37.17, 43.43)	126.26(117.82,135.3)
5-9	469.12 (460.93, 477.47)	665.46 (651.54, 679.69)	676.83 (664.78, 689.11)	559.05 (540.24, 578.51)	252.78 (241.03, 265.11)	753.07(719.55,788.16)
10-14	671.54 (660.27, 683.01)	898.69 (880.68, 917.08)	890.38 (875.72, 905.29)	830.25 (804.69, 856.63)	404.48 (387.45, 422.27)	931.07(892.38,971.45)
15-19	661.94 (651.00, 673.07)	788.62 (773.01, 804.55)	734.24 (722.42, 746.24)	881.93 (855.82, 908.83)	475.60 (456.47, 495.54)	626.23(600.37,653.2)
20-24	654.10 (643.43, 664.95)	739.23 (724.78, 753.97)	662.78 (652.43, 673.30)	924.90 (898.47, 952.10)	534.17 (513.46, 555.73)	489.85(469.97,510.58)
25-29	650.56 (640.08, 661.22)	749.09 (734.70, 763.75)	667.32 (657.37, 677.42)	953.88 (927.56, 980.95)	575.84 (554.22, 598.31)	517.52(497.69,538.14)
30-34	648.01 (637.68, 658.51)	759.08 (744.73, 773.70)	668.69 (659.17, 678.35)	981.86 (955.74, 1008.69)	613.84 (591.50, 637.02)	543.97(524.06,564.63)
35-39	644.54 (634.98, 654.25)	768.25 (754.72, 782.02)	665.82 (657.38, 674.37)	1007.70 (983.51, 1032.48)	653.11 (631.41, 675.56)	568.71(550.04,588.02)
40-44	615.78 (606.59, 625.11)	749.74 (736.56, 763.15)	649.42 (641.37, 657.57)	969.13 (946.34, 992.47)	655.55 (633.97, 677.86)	564.05(545.99,582.71)
45-49	563.17 (554.36, 572.12)	701.81 (689.08, 714.77)	621.48 (613.73, 629.34)	861.81 (840.89, 883.24)	620.24 (599.29, 641.92)	532.71(515.43,550.58)
50-54	510.58 (501.89, 519.42)	652.68 (640.00, 665.61)	592.77 (585.11, 600.53)	747.63 (728.09, 767.68)	581.89 (560.88, 603.69)	502.2(485.22,519.78)
55-59	456.59 (447.75, 465.60)	599.89 (586.80, 613.27)	562.92 (555.15, 570.80)	625.45 (606.91, 644.56)	538.64 (517.16, 561.01)	470.39(453.03,488.42)
60-64	407.56 (398.24, 417.11)	537.53 (523.73, 551.69)	516.66 (508.74, 524.71)	527.25 (509.02, 546.12)	484.33 (462.43, 507.27)	432.67(414.97,451.12)
65-69	363.14 (352.44, 374.17)	466.25 (450.57, 482.48)	453.78 (445.22, 462.51)	454.69 (434.76, 475.53)	421.48 (397.25, 447.20)	393.22(373.44,414.04)
70-74	317.60 (305.86, 329.80)	393.02 (376.19, 410.59)	387.69 (379.08, 396.50)	378.26 (357.93, 399.74)	358.01 (332.41, 385.59)	353.3(332.22,375.71)
75-79	270.32 (257.17, 284.13)	316.21 (298.06, 335.45)	315.56 (306.97, 324.38)	299.82 (279.24, 321.91)	295.21 (267.45, 325.85)	313.54(290.65,338.23)
80-84	215.59 (200.38, 231.95)	243.35 (223.11, 265.42)	242.58 (233.94, 251.54)	226.69 (205.83, 249.66)	231.81 (200.38, 268.17)	251.08(226.38,278.46)
85-89	154.42 (135.63, 175.81)	176.33 (151.99, 204.57)	173.16 (163.84, 183.02)	159.95 (138.24, 185.08)	166.86 (129.25, 215.41)	170.42(143.27,202.73)
90-94	92.62 (68.12, 125.95)	107.41 (77.21, 149.41)	103.38 (92.57, 115.44)	94.58 (71.56, 125.01)	100.86 (57.03, 178.36)	96.02(65.41,140.97)
95+	31.03 (10.33, 93.22)	36.14 (12.12, 107.78)	34.40 (24.15, 49.00)	31.34 (13.88, 70.77)	33.94 (4.83, 238.19)	29.39(7.88,109.58)

Age group (years)	Total population					
	AFR	EMR	EUR	AMR	SEAR	WPR
**Prevalence**						
0-4	70.85 (68.34, 73.45)	105.26 (100.84, 109.88)	105.80 (99.79, 112.18)	87.34 (76.50, 99.72)	38.08 (31.44, 46.12)	127.53 (110.93, 146.62)
5-9	1124.83 (1106.65, 1143.32)	1641.76 (1612.46, 1671.59)	1600.95 (1569.22, 1633.32)	1344.20 (1285.91, 1405.13)	590.63 (553.14, 630.65)	1852.32 (1761.92, 1947.36)
10-14	2972.95 (2930.91, 3015.58)	4290.27 (4224.63, 4356.94)	4068.93 (4006.01, 4132.84)	3630.82 (3510.28, 3755.50)	1634.06 (1553.40, 1718.91)	4558.09 (4377.85, 4745.74)
15-19	4037.59 (3983.00, 4092.93)	5556.74 (5475.69, 5638.98)	5067.96 (4995.96, 5140.99)	5241.44 (5083.89, 5403.87)	2482.48 (2370.96, 2599.24)	5046.15 (4858.37, 5241.19)
20-24	4390.22 (4332.28, 4448.94)	5719.38 (5638.02, 5801.91)	5051.25 (4982.87, 5120.58)	6224.63 (6048.57, 6405.82)	3080.49 (2949.84, 3216.92)	4179.83 (4028.96, 4336.36)
25-29	4477.85 (4419.76, 4536.70)	5713.68 (5634.01, 5794.48)	4978.10 (4913.80, 5043.25)	6907.58 (6722.00, 7098.28)	3511.29 (3368.88, 3659.71)	3704.77 (3576.07, 3838.11)
30-34	4466.81 (4409.58, 4524.78)	5701.94 (5623.74, 5781.24)	4957.93 (4896.90, 5019.71)	7393.14 (7204.06, 7587.19)	3828.39 (3678.98, 3983.86)	3583.18 (3463.17, 3707.36)
35-39	4420.29 (4367.51, 4473.71)	5691.30 (5618.42, 5765.13)	4954.05 (4899.72, 5008.99)	7768.71 (7589.52, 7952.13)	4097.26 (3951.42, 4248.47)	3627.21 (3517.62, 3740.21)
40-44	4344.85 (4292.88, 4397.45)	5680.70 (5608.42, 5753.91)	4969.39 (4916.54, 5022.80)	8034.66 (7857.38, 8215.94)	4326.79 (4175.81, 4483.23)	3723.38 (3614.40, 3835.64)
45-49	4213.96 (4161.90, 4266.66)	5600.18 (5527.48, 5673.85)	5007.45 (4954.67, 5060.79)	7969.63 (7794.34, 8148.85)	4442.09 (4286.23, 4603.62)	3767.63 (3657.98, 3880.57)
50-54	4051.14 (3997.74, 4105.25)	5481.84 (5406.94, 5557.77)	5062.02 (5007.85, 5116.78)	7638.04 (7462.70, 7817.50)	4471.03 (4307.30, 4640.98)	3770.34 (3658.32, 3885.78)
55-59	3874.86 (3818.28, 3932.28)	5338.24 (5258.15, 5419.55)	5148.79 (5091.45, 5206.78)	7136.72 (6957.53, 7320.53)	4445.33 (4270.66, 4627.14)	3756.51 (3637.63, 3879.28)
60-64	3723.22 (3660.99, 3786.51)	5193.24 (5104.39, 5283.63)	5260.78 (5198.11, 5324.20)	6574.70 (6389.62, 6765.13)	4378.53 (4189.92, 4575.63)	3723.92 (3596.85, 3855.48)
65-69	3614.07 (3538.12, 3691.66)	5051.85 (4942.02, 5164.13)	5342.34 (5266.92, 5418.85)	6098.93 (5885.42, 6320.18)	4253.03 (4026.58, 4492.21)	3722.51 (3570.02, 3881.52)
70-74	3529.14 (3440.22, 3620.35)	4908.42 (4779.72, 5040.58)	5400.93 (5316.27, 5486.95)	5667.49 (5434.87, 5910.06)	4083.72 (3826.67, 4358.04)	3719.80 (3546.44, 3901.64)
75-79	3460.85 (3352.96, 3572.20)	4716.00 (4561.97, 4875.23)	5433.54 (5337.36, 5531.45)	5243.52 (4985.52, 5514.87)	3904.27 (3602.36, 4231.48)	3732.91 (3531.25, 3946.08)
80-84	3390.98 (3251.23, 3536.73)	4449.91 (4257.72, 4650.77)	5397.70 (5286.05, 5511.71)	4809.24 (4517.58, 5119.73)	3714.03 (3336.68, 4134.05)	3704.85 (3460.42, 3966.53)
85-89	3152.05 (2954.16, 3363.20)	4002.27 (3742.64, 4279.92)	5139.62 (5002.36, 5280.65)	4295.75 (3951.87, 4669.56)	3399.45 (2886.06, 4004.16)	3399.25 (3087.47, 3742.52)
90-94	2761.86 (2437.70, 3129.14)	3386.03 (2988.41, 3836.55)	4655.37 (4463.65, 4855.32)	3695.72 (3247.07, 4206.37)	2954.26 (2188.45, 3988.06)	2853.85 (2413.94, 3373.93)
95+	2310.85 (1742.81, 3064.04)	2701.57 (2062.42, 3538.80)	3960.81 (3648.78, 4299.53)	2997.50 (2361.93, 3804.09)	2436.50 (1266.26, 4688.26)	2088.73 (1454.60, 2999.30)
**YLDs**						
0-4	70.85 (68.34, 73.45)	105.26 (100.84, 109.88)	105.80 (99.79, 112.18)	10.92 (9.59, 12.43)	4.71 (3.92, 5.67)	15.96 (13.90, 18.32)

Age group (years)	Total population					
	AFR	EMR	EUR	AMR	SEAR	WPR
5-9	1124.83 (1106.65, 1143.32)	1641.76 (1612.46, 1671.59)	1600.95 (1569.22, 1633.32)	168.19 (160.99, 175.71)	72.83 (68.33, 77.63)	232.03 (220.73, 243.92)
10-14	2972.95 (2930.91, 3015.58)	4290.27 (4224.63, 4356.94)	4068.93 (4006.01, 4132.84)	450.22 (435.34, 465.61)	200.78 (191.06, 210.99)	567.44 (544.89, 590.92)
15-19	4037.59 (3983.00, 4092.93)	5556.74 (5475.69, 5638.98)	5067.96 (4995.96, 5140.99)	641.14 (621.87, 661.01)	302.97 (289.58, 316.97)	623.83 (600.45, 648.12)
20-24	4390.22 (4332.28, 4448.94)	5719.38 (5638.02, 5801.91)	5051.25 (4982.87, 5120.58)	754.04 (732.66, 776.04)	373.30 (357.69, 389.60)	512.83 (494.16, 532.20)
25-29	4477.85 (4419.76, 4536.70)	5713.68 (5634.01, 5794.48)	4978.10 (4913.80, 5043.25)	829.70 (807.31, 852.72)	422.46 (405.53, 440.09)	451.89 (436.03, 468.32)
30-34	4466.81 (4409.58, 4524.78)	5701.94 (5623.74, 5781.24)	4957.93 (4896.90, 5019.71)	882.40 (859.69, 905.71)	457.47 (439.80, 475.84)	434.79 (420.06, 450.04)
35-39	4420.29 (4367.51, 4473.71)	5691.30 (5618.42, 5765.13)	4954.05 (4899.72, 5008.99)	920.26 (898.87, 942.15)	485.98 (468.85, 503.74)	437.69 (424.31, 451.50)
40-44	4344.85 (4292.88, 4397.45)	5680.70 (5608.42, 5753.91)	4969.39 (4916.54, 5022.80)	944.05 (923.02, 965.55)	509.14 (491.51, 527.41)	446.39 (433.14, 460.05)
45-49	4213.96 (4161.90, 4266.66)	5600.18 (5527.48, 5673.85)	5007.45 (4954.67, 5060.79)	928.86 (908.19, 950.00)	518.59 (500.49, 537.35)	448.27 (435.01, 461.94)
50-54	4051.14 (3997.74, 4105.25)	5481.84 (5406.94, 5557.77)	5062.02 (5007.85, 5116.78)	882.64 (862.08, 903.69)	517.28 (498.38, 536.90)	445.01 (431.54, 458.91)
55-59	3874.86 (3818.28, 3932.28)	5338.24 (5258.15, 5419.55)	5148.79 (5091.45, 5206.78)	815.18 (794.33, 836.58)	508.40 (488.37, 529.24)	438.37 (424.18, 453.04)
60-64	3723.22 (3660.99, 3786.51)	5193.24 (5104.39, 5283.63)	5260.78 (5198.11, 5324.20)	739.90 (718.56, 761.88)	493.91 (472.47, 516.33)	428.46 (413.42, 444.04)
65-69	3614.07 (3538.12, 3691.66)	5051.85 (4942.02, 5164.13)	5342.34 (5266.92, 5418.85)	673.68 (649.41, 698.87)	470.44 (445.05, 497.27)	420.42 (402.63, 438.99)
70-74	3529.14 (3440.22, 3620.35)	4908.42 (4779.72, 5040.58)	5400.93 (5316.27, 5486.95)	612.70 (586.61, 639.94)	441.79 (413.35, 472.19)	410.76 (390.84, 431.69)
75-79	3460.85 (3352.96, 3572.20)	4716.00 (4561.97, 4875.23)	5433.54 (5337.36, 5531.45)	553.30 (524.81, 583.34)	411.62 (378.68, 447.42)	402.42 (379.59, 426.61)
80-84	3390.98 (3251.23, 3536.73)	4449.91 (4257.72, 4650.77)	5397.70 (5286.05, 5511.71)	494.37 (462.65, 528.26)	382.40 (341.67, 427.99)	388.84 (361.58, 418.15)
85-89	3152.05 (2954.16, 3363.20)	4002.27 (3742.64, 4279.92)	5139.62 (5002.36, 5280.65)	429.26 (392.43, 469.55)	341.16 (286.36, 406.44)	346.99 (312.64, 385.12)
90-94	2761.86 (2437.70, 3129.14)	3386.03 (2988.41, 3836.55)	4655.37 (4463.65, 4855.32)	359.84 (312.40, 414.48)	289.15 (208.63, 400.74)	283.25 (235.41, 340.81)
95+	2310.85 (1742.81, 3064.04)	2701.57 (2062.42, 3538.80)	3960.81 (3648.78, 4299.53)	284.15 (218.12, 370.17)	232.40 (112.76, 478.98)	200.91 (133.67, 301.99)

Notes: YLDs, years lived with disability; CI, confidence interval; WHO, world health organization; AFR, Africa Region; AMR, Region of the Americas, EMR, Eastern Mediterranean Region; EUR, European Region; SEAR, South-East Asian Region; WPR, Western Pacific Region.

Source: Institute for Health Metrics and Evaluation. Used with permission. All rights reserved.

Table S10. Relative risk for anxiety disorders incidence, prevalence, and years lived with disability of each period compared with the reference (2002–2006) and the corresponding 95% CIs by sex and WHO region.

Periods	Total population					
	AFR	EMR	EUR	AMR	SEAR	WPR
**Incidence**						
1992-1996	1.0132 (0.9892, 1.0377)	1.0246 (0.9988, 1.0511)	0.9936 (0.9822, 1.0052)	0.9127 (0.8897, 0.9362)	1.0329 (0.9809, 1.0878)	1.0282(0.9838,1.0745)
1997-2001	1.0083 (0.9945, 1.0223)	1.0163 (1.0008, 1.0320)	0.9914 (0.9822, 1.0007)	0.9769 (0.9588, 0.9954)	0.9925 (0.9608, 1.0252)	1.051(1.0212,1.0816)
2002-2006	1.0000 (1.0000, 1.0000)	1.0000 (1.0000, 1.0000)	1.0000 (1.0000, 1.0000)	1.0000 (1.0000, 1.0000)	1.0000 (1.0000, 1.0000)	1.0000(1.0000,1.0000)
2007-2011	1.0073 (0.9938, 1.0210)	1.0195 (1.0043, 1.0349)	1.0215 (1.0120, 1.0310)	0.9889 (0.9709, 1.0072)	1.0351 (1.0028, 1.0684)	0.9875(0.9594,1.0165)
2012-2016	1.0100 (0.9863, 1.0343)	1.0347 (1.0088, 1.0613)	1.0157 (1.0038, 1.0277)	0.9348 (0.9115, 0.9588)	1.0324 (0.9807, 1.0868)	0.9721(0.9300,1.0161)
2017-2021	1.0680 (1.0316, 1.1058)	1.0983 (1.0588, 1.1394)	1.0868 (1.0707, 1.1032)	1.0231 (0.9892, 1.0582)	1.0918 (1.0147, 1.1749)	1.0232(0.9618,1.0884)
**Prevalence**						
1992-1996	1.0091 (0.9988, 1.0195)	1.0127 (1.0019, 1.0236)	1.0053 (0.9976, 1.0131)	0.8587 (0.8430, 0.8747)	1.0248 (0.9924, 1.0582)	1.0306 (1.0065, 1.0553)
1997-2001	1.0067 (0.9987, 1.0148)	1.0025 (0.9939, 1.0112)	0.9979 (0.9904, 1.0054)	0.9643 (0.9484, 0.9805)	0.9901 (0.9632, 1.0177)	1.0572 (1.0354, 1.0795)
2002-2006	1.0000 (1.0000, 1.0000)	1.0000 (1.0000, 1.0000)	1.0000 (1.0000, 1.0000)	1.0000 (1.0000, 1.0000)	1.0000 (1.0000, 1.0000)	1.0000 (1.0000, 1.0000)
2007-2011	1.0067 (0.9990, 1.0144)	1.0218 (1.0135, 1.0303)	1.0241 (1.0165, 1.0317)	0.9687 (0.9533, 0.9844)	1.0314 (1.0048, 1.0587)	0.9757 (0.9556, 0.9962)
2012-2016	1.0097 (0.9998, 1.0197)	1.0335 (1.0229, 1.0442)	1.0272 (1.0193, 1.0351)	0.8927 (0.8771, 0.9085)	1.0326 (1.0012, 1.0649)	0.9461 (0.9240, 0.9686)
2017-2021	1.0662 (1.0526, 1.0800)	1.0928 (1.0785, 1.1073)	1.0900 (1.0813, 1.0989)	0.9777 (0.9590, 0.9967)	1.0848 (1.0448, 1.1262)	0.9929 (0.9662, 1.0203)
**YLDs**						
1992-1996	1.0091 (0.9988, 1.0195)	1.0127 (1.0019, 1.0236)	1.0053 (0.9976, 1.0131)	0.8611 (0.8450, 0.8774)	1.0247 (0.9914, 1.0590)	1.0289 (1.0039, 1.0545)
1997-2001	1.0067 (0.9987, 1.0148)	1.0025 (0.9939, 1.0112)	0.9979 (0.9904, 1.0054)	0.9651 (0.9490, 0.9815)	0.9896 (0.9627, 1.0172)	1.0563 (1.0341, 1.0790)
2002-2006	1.0000 (1.0000, 1.0000)	1.0000 (1.0000, 1.0000)	1.0000 (1.0000, 1.0000)	1.0000 (1.0000, 1.0000)	1.0000 (1.0000, 1.0000)	1.0000 (1.0000, 1.0000)
2007-2011	1.0067 (0.9990, 1.0144)	1.0218 (1.0135, 1.0303)	1.0241 (1.0165, 1.0317)	0.9689 (0.9533, 0.9848)	1.0348 (1.0080, 1.0623)	0.9760 (0.9555, 0.9970)
2012-2016	1.0097 (0.9998, 1.0197)	1.0335 (1.0229, 1.0442)	1.0272 (1.0193, 1.0351)	0.8928 (0.8769, 0.9089)	1.0369 (1.0045, 1.0703)	0.9470 (0.9240, 0.9705)

Periods	Total population					
	AFR	EMR	EUR	AMR	SEAR	WPR
2017-2021	1.0662 (1.0526, 1.0800)	1.0928 (1.0785, 1.1073)	1.0900 (1.0813, 1.0989)	0.9757 (0.9563, 0.9954)	1.0895 (1.0472, 1.1334)	0.9917 (0.9634, 1.0208)

Notes: YLDs, years lived with disability; CI, confidence interval; WHO, world health organization; AFR, Africa Region; AMR, Region of the Americas, EMR, Eastern Mediterranean Region; EUR, European Region; SEAR, South-East Asian Region; WPR, Western Pacific Region.

Source: Institute for Health Metrics and Evaluation. Used with permission. *All rights reserved.*

Table S11. Relative risk for anxiety disorders incidence, prevalence, and years lived with disability of each birth cohort compared with the reference (cohort 1957-1961) and the corresponding 95% CIs by sex and WHO region.

Cohorts	Total population					
	AFR	EMR	EUR	AMR	SEAR	WPR
			Incidence			
1897-1901	0.92 (0.03, 27.32)	0.90 (0.03, 28.06)	0.91 (0.27, 3.10)	1.00 (0.06, 15.90)	0.97 (0.00, 1207.25)	1.07 (0, 472.75)
1902-1906	0.92 (0.38, 2.23)	0.91 (0.35, 2.36)	0.91 (0.66, 1.24)	1.00 (0.44, 2.24)	0.97 (0.16, 5.85)	1.05 (0.25, 4.37)
1907-1911	0.93 (0.65, 1.31)	0.91 (0.61, 1.35)	0.91 (0.80, 1.03)	0.99 (0.69, 1.41)	0.97 (0.48, 1.96)	1.04 (0.62, 1.75)
1912-1916	0.93 (0.79, 1.11)	0.92 (0.75, 1.14)	0.91 (0.85, 0.98)	0.98 (0.80, 1.20)	0.97 (0.67, 1.41)	1.03 (0.8, 1.33)
1917-1921	0.94 (0.85, 1.05)	0.94 (0.83, 1.06)	0.91 (0.87, 0.96)	0.97 (0.85, 1.11)	0.98 (0.78, 1.22)	1.05 (0.9, 1.23)
1922-1926	0.95 (0.89, 1.02)	0.95 (0.87, 1.03)	0.92 (0.89, 0.95)	0.97 (0.88, 1.06)	0.99 (0.85, 1.14)	1.07 (0.96, 1.19)
1927-1931	0.96 (0.91, 1.01)	0.95 (0.90, 1.01)	0.92 (0.89, 0.94)	0.96 (0.89, 1.04)	1.00 (0.90, 1.11)	1.07 (0.99, 1.16)
1932-1936	0.96 (0.93, 1.00)	0.96 (0.92, 1.01)	0.93 (0.90, 0.95)	0.96 (0.91, 1.02)	1.01 (0.93, 1.09)	1.08 (1.01, 1.15)
1937-1941	0.97 (0.94, 1.00)	0.96 (0.93, 1.00)	0.94 (0.92, 0.95)	0.96 (0.91, 1.01)	1.01 (0.95, 1.08)	1.06 (1.01, 1.13)
1942-1946	0.97 (0.95, 1.00)	0.96 (0.94, 0.99)	0.95 (0.94, 0.97)	0.96 (0.92, 1.00)	1.01 (0.96, 1.06)	1.04 (0.99, 1.09)
1947-1951	0.98 (0.96, 1.00)	0.97 (0.95, 1.00)	0.98 (0.96, 0.99)	0.96 (0.93, 1.00)	1.00 (0.95, 1.05)	1.03 (0.98, 1.07)
1952-1956	0.99 (0.97, 1.01)	0.98 (0.96, 1.00)	0.99 (0.97, 1.00)	0.98 (0.95, 1.01)	0.99 (0.95, 1.03)	1.01 (0.97, 1.05)
1957-1961	1.00 (1.00, 1.00)	1.00 (1.00, 1.00)	1.00 (1.00, 1.00)	1.00 (1.00, 1.00)	1.00 (1.00, 1.00)	1.00 (1.00, 1.00)
1962-1966	1.01 (0.99, 1.02)	1.02 (1.00, 1.04)	1.02 (1.01, 1.04)	1.02 (0.99, 1.05)	1.02 (0.98, 1.06)	1.00 (0.97, 1.04)
1967-1971	1.01 (1.00, 1.03)	1.04 (1.02, 1.06)	1.05 (1.03, 1.06)	1.03 (1.01, 1.06)	1.04 (1.01, 1.08)	1.00 (0.97, 1.03)
1972-1976	1.02 (1.01, 1.04)	1.05 (1.04, 1.07)	1.05 (1.04, 1.07)	1.04 (1.02, 1.07)	1.07 (1.03, 1.11)	0.99 (0.96, 1.03)
1977-1981	1.03 (1.01, 1.05)	1.07 (1.05, 1.09)	1.06 (1.04, 1.07)	1.07 (1.04, 1.10)	1.10 (1.06, 1.14)	0.97 (0.93, 1.01)
1982-1986	1.04 (1.02, 1.05)	1.10 (1.08, 1.12)	1.07 (1.05, 1.08)	1.11 (1.07, 1.14)	1.11 (1.07, 1.16)	0.93 (0.89, 0.97)
1987-1991	1.05 (1.03, 1.07)	1.12 (1.09, 1.14)	1.08 (1.07, 1.10)	1.14 (1.10, 1.17)	1.12 (1.07, 1.16)	0.92 (0.89, 0.96)
1992-1996	1.07 (1.05, 1.08)	1.12 (1.10, 1.14)	1.13 (1.11, 1.15)	1.16 (1.12, 1.19)	1.12 (1.07, 1.17)	0.94 (0.90, 0.98)

Cohorts	Total population					
	AFR	EMR	EUR	AMR	SEAR	WPR
1997-2001	1.07 (1.05, 1.09)	1.12 (1.10, 1.14)	1.18 (1.16, 1.20)	1.18 (1.14, 1.22)	1.17 (1.12, 1.22)	0.95 (0.91, 1.00)
2002-2006	1.07 (1.05, 1.09)	1.12 (1.10, 1.15)	1.22 (1.20, 1.25)	1.20 (1.16, 1.25)	1.25 (1.19, 1.31)	0.95 (0.91, 1.00)
2007-2011	1.08 (1.06, 1.10)	1.14 (1.11, 1.16)	1.24 (1.22, 1.27)	1.22 (1.17, 1.27)	1.32 (1.25, 1.39)	0.93 (0.88, 0.98)
2012-2016	1.10 (1.08, 1.12)	1.17 (1.14, 1.20)	1.28 (1.24, 1.31)	1.26 (1.20, 1.32)	1.35 (1.26, 1.45)	0.90 (0.85, 0.96)
2017-2021	1.15 (1.11, 1.19)	1.23 (1.17, 1.29)	1.35 (1.28, 1.43)	1.33 (1.19, 1.48)	1.43 (1.22, 1.67)	0.94 (0.82, 1.08)
**Prevalence**						
1897-1901	0.92 (0.39, 2.15)	0.88 (0.39, 1.99)	0.89 (0.68, 1.18)	1.07 (0.49, 2.32)	0.94 (0.09, 10.18)	1.16 (0.22, 6.10)
1902-1906	0.92 (0.66, 1.28)	0.85 (0.61, 1.20)	0.90 (0.81, 1.00)	1.04 (0.74, 1.45)	0.94 (0.38, 2.28)	1.13 (0.64, 2.00)
1907-1911	0.92 (0.78, 1.07)	0.83 (0.70, 0.99)	0.91 (0.86, 0.96)	1.01 (0.85, 1.21)	0.94 (0.62, 1.43)	1.14 (0.89, 1.47)
1912-1916	0.92 (0.84, 1.01)	0.84 (0.76, 0.92)	0.92 (0.89, 0.96)	1.00 (0.89, 1.12)	0.96 (0.75, 1.23)	1.15 (1.00, 1.33)
1917-1921	0.93 (0.88, 0.99)	0.87 (0.81, 0.92)	0.93 (0.90, 0.95)	0.97 (0.90, 1.06)	0.98 (0.84, 1.16)	1.17 (1.06, 1.29)
1922-1926	0.94 (0.90, 0.98)	0.90 (0.86, 0.94)	0.94 (0.92, 0.97)	0.96 (0.90, 1.03)	1.00 (0.89, 1.12)	1.16 (1.08, 1.25)
1927-1931	0.94 (0.91, 0.98)	0.92 (0.89, 0.95)	0.93 (0.91, 0.94)	0.95 (0.90, 1.01)	1.01 (0.92, 1.10)	1.13 (1.06, 1.20)
1932-1936	0.95 (0.93, 0.98)	0.93 (0.91, 0.96)	0.93 (0.92, 0.95)	0.95 (0.91, 1.00)	1.01 (0.95, 1.09)	1.10 (1.05, 1.16)
1937-1941	0.96 (0.94, 0.99)	0.94 (0.92, 0.96)	0.95 (0.93, 0.96)	0.95 (0.92, 0.99)	1.01 (0.96, 1.08)	1.07 (1.03, 1.12)
1942-1946	0.97 (0.95, 0.99)	0.95 (0.93, 0.96)	0.98 (0.96, 0.99)	0.96 (0.93, 0.99)	1.00 (0.95, 1.05)	1.04 (1.00, 1.09)
1947-1951	0.98 (0.96, 1.00)	0.96 (0.94, 0.98)	1.00 (0.98, 1.01)	0.97 (0.94, 1.00)	0.99 (0.95, 1.04)	1.03 (0.99, 1.06)
1952-1956	0.99 (0.98, 1.01)	0.98 (0.96, 0.99)	0.99 (0.98, 1.00)	0.99 (0.96, 1.01)	0.99 (0.95, 1.03)	1.01 (0.98, 1.05)
1957-1961	1.00 (1.00, 1.00)	1.00 (1.00, 1.00)	1.00 (1.00, 1.00)	1.00 (1.00, 1.00)	1.00 (1.00, 1.00)	1.00 (1.00, 1.00)
1962-1966	1.01 (0.99, 1.02)	1.02 (1.00, 1.03)	1.02 (1.01, 1.03)	1.01 (0.99, 1.04)	1.02 (0.98, 1.06)	0.99 (0.96, 1.03)
1967-1971	1.01 (1.00, 1.03)	1.03 (1.02, 1.05)	1.05 (1.04, 1.06)	1.02 (1.00, 1.05)	1.04 (1.00, 1.08)	0.98 (0.95, 1.01)
1972-1976	1.02 (1.01, 1.03)	1.05 (1.03, 1.06)	1.05 (1.04, 1.06)	1.03 (1.01, 1.06)	1.06 (1.02, 1.10)	0.95 (0.92, 0.98)
1977-1981	1.03 (1.01, 1.04)	1.06 (1.04, 1.07)	1.05 (1.04, 1.07)	1.06 (1.04, 1.09)	1.08 (1.04, 1.12)	0.92 (0.89, 0.95)

Cohorts	Total population					
	AFR	EMR	EUR	AMR	SEAR	WPR
1982-1986	1.04 (1.03, 1.05)	1.08 (1.07, 1.10)	1.07 (1.05, 1.08)	1.10 (1.07, 1.13)	1.09 (1.04, 1.13)	0.90 (0.87, 0.93)
1987-1991	1.05 (1.04, 1.07)	1.10 (1.08, 1.11)	1.08 (1.07, 1.10)	1.12 (1.09, 1.15)	1.10 (1.05, 1.15)	0.91 (0.88, 0.95)
1992-1996	1.06 (1.05, 1.08)	1.09 (1.07, 1.10)	1.13 (1.11, 1.14)	1.14 (1.10, 1.17)	1.13 (1.08, 1.19)	0.91 (0.87, 0.95)
1997-2001	1.06 (1.04, 1.08)	1.07 (1.06, 1.09)	1.18 (1.16, 1.20)	1.17 (1.13, 1.21)	1.20 (1.14, 1.26)	0.90 (0.86, 0.94)
2002-2006	1.06 (1.04, 1.07)	1.08 (1.06, 1.10)	1.23 (1.20, 1.25)	1.19 (1.14, 1.24)	1.27 (1.19, 1.34)	0.89 (0.84, 0.93)
2007-2011	1.07 (1.05, 1.09)	1.11 (1.09, 1.13)	1.25 (1.22, 1.28)	1.21 (1.15, 1.28)	1.31 (1.22, 1.41)	0.87 (0.82, 0.92)
2012-2016	1.09 (1.07, 1.12)	1.15 (1.11, 1.18)	1.28 (1.24, 1.33)	1.26 (1.16, 1.37)	1.36 (1.21, 1.52)	0.86 (0.78, 0.94)
2017-2021	1.14 (1.06, 1.22)	1.20 (1.10, 1.31)	1.35 (1.19, 1.54)	1.33 (0.98, 1.81)	1.44 (0.93, 2.24)	0.89 (0.64, 1.22)
YLDs						
1897-1901	0.92 (0.39, 2.15)	0.88 (0.39, 1.99)	0.89 (0.65, 1.21)	1.10 (0.46, 2.58)	0.93 (0.07, 12.94)	1.17 (0.18, 7.57)
1902-1906	0.92 (0.66, 1.28)	0.85 (0.61, 1.20)	0.89 (0.79, 1.00)	1.06 (0.73, 1.53)	0.92 (0.35, 2.45)	1.14 (0.60, 2.14)
1907-1911	0.92 (0.78, 1.07)	0.83 (0.70, 0.99)	0.91 (0.86, 0.96)	1.03 (0.85, 1.25)	0.93 (0.59, 1.46)	1.15 (0.87, 1.52)
1912-1916	0.92 (0.84, 1.01)	0.84 (0.76, 0.92)	0.92 (0.89, 0.96)	1.01 (0.89, 1.14)	0.95 (0.73, 1.24)	1.15 (0.99, 1.35)
1917-1921	0.93 (0.88, 0.99)	0.87 (0.81, 0.92)	0.92 (0.90, 0.95)	0.99 (0.90, 1.08)	0.97 (0.82, 1.15)	1.17 (1.05, 1.30)
1922-1926	0.94 (0.90, 0.98)	0.90 (0.86, 0.94)	0.94 (0.92, 0.96)	0.97 (0.90, 1.04)	0.99 (0.87, 1.11)	1.16 (1.07, 1.25)
1927-1931	0.94 (0.91, 0.98)	0.92 (0.89, 0.95)	0.93 (0.91, 0.94)	0.96 (0.91, 1.02)	1.00 (0.91, 1.09)	1.13 (1.06, 1.20)
1932-1936	0.95 (0.93, 0.98)	0.93 (0.91, 0.96)	0.93 (0.92, 0.95)	0.96 (0.91, 1.00)	1.01 (0.94, 1.08)	1.10 (1.05, 1.16)
1937-1941	0.96 (0.94, 0.99)	0.94 (0.92, 0.96)	0.95 (0.93, 0.96)	0.96 (0.92, 1.00)	1.01 (0.95, 1.07)	1.07 (1.03, 1.13)
1942-1946	0.97 (0.95, 0.99)	0.95 (0.93, 0.96)	0.97 (0.96, 0.99)	0.96 (0.93, 0.99)	1.00 (0.95, 1.05)	1.04 (1.00, 1.09)
1947-1951	0.98 (0.96, 1.00)	0.96 (0.94, 0.98)	1.00 (0.98, 1.01)	0.97 (0.94, 1.00)	0.99 (0.94, 1.03)	1.03 (0.99, 1.07)
1952-1956	0.99 (0.98, 1.01)	0.98 (0.96, 0.99)	0.99 (0.98, 1.00)	0.99 (0.96, 1.01)	0.99 (0.95, 1.03)	1.01 (0.98, 1.05)
1957-1961	1.00 (1.00, 1.00)	1.00 (1.00, 1.00)	1.00 (1.00, 1.00)	1.00 (1.00, 1.00)	1.00 (1.00, 1.00)	1.00 (1.00, 1.00)
1962-1966	1.01 (0.99, 1.02)	1.02 (1.00, 1.03)	1.02 (1.01, 1.03)	1.01 (0.99, 1.04)	1.02 (0.98, 1.06)	0.99 (0.96, 1.03)

Cohorts	Total population					
	**AFR**	**EMR**	**EUR**	**AMR**	**SEAR**	**WPR**
1967-1971	1.01 (1.00, 1.03)	1.03 (1.02, 1.05)	1.05 (1.04, 1.06)	1.02 (1.00, 1.04)	1.04 (1.00, 1.08)	0.98 (0.95, 1.01)
1972-1976	1.02 (1.01, 1.03)	1.05 (1.03, 1.06)	1.05 (1.04, 1.06)	1.03 (1.01, 1.06)	1.06 (1.02, 1.10)	0.95 (0.92, 0.98)
1977-1981	1.03 (1.01, 1.04)	1.06 (1.04, 1.07)	1.06 (1.04, 1.07)	1.06 (1.04, 1.09)	1.08 (1.04, 1.12)	0.92 (0.89, 0.96)
1982-1986	1.04 (1.03, 1.05)	1.08 (1.07, 1.10)	1.07 (1.06, 1.08)	1.10 (1.07, 1.13)	1.09 (1.05, 1.14)	0.90 (0.87, 0.94)
1987-1991	1.05 (1.04, 1.07)	1.10 (1.08, 1.11)	1.08 (1.07, 1.10)	1.12 (1.09, 1.15)	1.10 (1.06, 1.15)	0.91 (0.88, 0.95)
1992-1996	1.06 (1.05, 1.08)	1.09 (1.07, 1.10)	1.13 (1.11, 1.15)	1.14 (1.10, 1.17)	1.14 (1.09, 1.19)	0.91 (0.88, 0.95)
1997-2001	1.06 (1.04, 1.08)	1.07 (1.06, 1.09)	1.19 (1.17, 1.20)	1.16 (1.12, 1.21)	1.21 (1.15, 1.27)	0.90 (0.87, 0.95)
2002-2006	1.06 (1.04, 1.07)	1.08 (1.06, 1.10)	1.23 (1.21, 1.25)	1.19 (1.14, 1.24)	1.27 (1.20, 1.35)	0.89 (0.85, 0.94)
2007-2011	1.07 (1.05, 1.09)	1.11 (1.09, 1.13)	1.25 (1.23, 1.28)	1.21 (1.15, 1.28)	1.32 (1.23, 1.42)	0.88 (0.83, 0.93)
2012-2016	1.09 (1.07, 1.12)	1.15 (1.11, 1.18)	1.29 (1.24, 1.33)	1.27 (1.17, 1.37)	1.37 (1.22, 1.53)	0.86 (0.79, 0.94)
2017-2021	1.14 (1.06, 1.22)	1.20 (1.10, 1.31)	1.36 (1.20, 1.55)	1.34 (1.00, 1.80)	1.46 (0.96, 2.22)	0.89 (0.65, 1.23)

Notes: YLDs, years lived with disability; CI, confidence interval; WHO, world health organization; AFR, Africa Region; AMR, Region of the Americas, EMR, Eastern Mediterranean Region; EUR, European Region; SEAR, South-East Asian Region; WPR, Western Pacific Region.

Source: Institute for Health Metrics and Evaluation. Used with permission. All rights reserved.

(A)

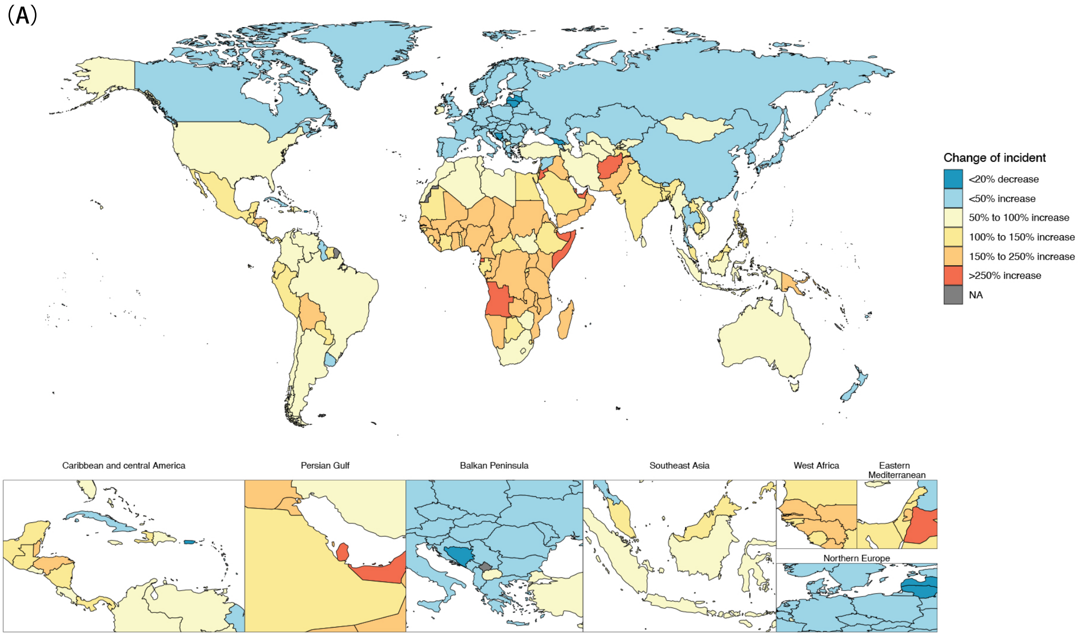

(B)

Age-standardized incidence rate in 1992 (per 100,000)	Leading countries in 1992	Leading countries in 2021	Age-standardized incidence rate in 2021 (per 100,000)	Percentage change in age-standardized incidence rate, 1992-2021	Percentage change in age-standardized YLD rate, 1992-2021	Percentage change in age-standardized prevalence rate, 1992-2021
978.76	1. Portugal	1. Portugal	1290.64	31.87	27.75	27.37
973.80	2. New Zealand	2. Brazil	1209.77	41.15	50.04	49.86
969.01	3. Norway	3. Lebanon	1168.05	42.77	36.15	35.92
921.74	4. Iran (Islamic Republic of)	4. Iran (Islamic Republic of)	1142.02	23.90	21.70	21.53
908.50	5. Switzerland	5. Paraguay	1123.41	29.30	25.66	25.90
882.70	6. Ireland	6. Ireland	1110.21	25.77	22.56	22.57
868.85	7. Paraguay	7. Bolivia (Plurinational State of)	1109.65	48.20	42.66	42.15
857.11	8. Brazil	8. France	1076.13	26.41	22.68	22.51
851.30	9. France	9. Germany	1075.74	31.22	26.12	25.99
835.62	10. Cyprus	10. Norway	1067.02	10.11	5.51	5.18
830.64	11. Malta	11. Peru	1065.08	43.55	39.04	71.23
830.51	12. Netherlands	12. Greece	1060.53	32.01	28.84	28.88
819.78	13. Germany	13. Netherlands	1058.90	27.50	20.27	20.44
818.12	14. Lebanon	14. Switzerland	1044.83	15.01	12.94	12.66
813.79	15. Austria	15. Austria	1029.45	26.50	23.60	22.95
805.67	16. Italy	16. Tunisia	1025.71	41.36	36.85	37.42
803.34	17. Greece	17. Italy	1016.76	26.20	22.03	21.52
796.01	18. San Marino	18. San Marino	1014.65	27.47	24.42	24.47
791.15	19. Australia	19. Monaco	1002.39	26.75	22.68	22.84
790.85	20. Monaco	20. New Zealand	1001.21	2.81	2.15	1.72

High SDI
High-middle SDI
Middle SDI
Low-middle SDI

Figure S1. Global distribution of relative changes in anxiety disorders incidence from 1992 to 2021 and the top 20 countries by age-standardized incidence in 1992 and 2021.

Notes: (A) Global map for the relative change of incidence from 1992 to 2021. (B) Ranking graphs for age-standardized incidence. Dashed lines indicate decreasing rankings, and solid lines indicate increasing rankings. YLDs, years lived with disability; SDI, socio-demographic index.

Source: Institute for Health Metrics and Evaluation. Used with permission. All rights reserved.

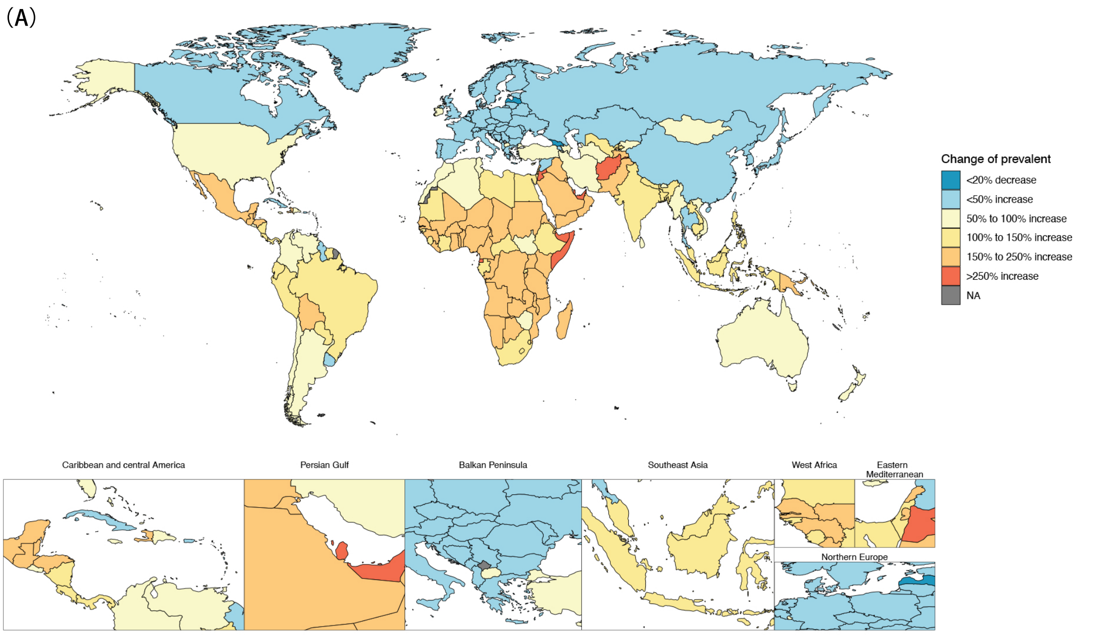

(B)

Age-standardized prevalence rate in 1992 (per 100,000)	Leading countries in 1992	Leading countries in 2021	Age-standardized prevalence rate in 1992 (per 100,000)	Percentage change in age-standardized prevalence rate, 1992-2021	Percentage change in age-standardized incidence rate, 1992-2021	Percentage change in age-standardized YLD rate, 1992-2021
7625.33	1. Portugal	1. Portugal	9712.44	27.37	31.87	27.75
7296.11	2. New Zealand	2. Brazil	9007.36	49.86	41.15	50.04
7173.44	3. Norway	3. Paraguay	8390.01	25.90	29.30	25.66
6754.19	4. Switzerland	4. Lebanon	8274.66	35.92	42.77	36.15
6746.03	5. Iran (Islamic Republic of)	5. Iran (Islamic Republic of)	8198.38	21.53	23.90	21.70
6664.04	6. Paraguay	6. Netherlands	7885.11	20.44	27.50	20.27
6546.93	7. Netherlands	7. Ireland	7883.66	22.57	25.77	22.56
6432.11	8. Ireland	8. France	7694.09	22.51	26.41	22.68
6280.16	9. France	9. Switzerland	7609.41	12.66	15.01	12.94
6088.11	10. Lebanon	10. Germany	7595.91	25.99	31.22	26.12
6029.06	11. Germany	11. Bolivia (Plurinational State of)	7571.02	42.15	48.20	42.66
6010.60	12. Brazil	12. Norway	7544.74	5.18	10.11	5.51
5940.58	13. Cyprus	13. New Zealand	7421.44	1.72	2.81	2.15
5907.93	14. Malta	14. Peru	7240.51	71.23	43.55	39.04
5742.41	15. Austria	15. Greece	7208.74	28.88	32.01	28.84
5608.02	16. Italy	16. Austria	7060.07	22.95	26.50	23.60
5593.37	17. Greece	17. Cyprus	6986.37	17.60	19.37	17.87
5550.95	18. Australia	18. Tunisia	6875.36	37.42	41.36	36.85
5507.02	29. San Marino	19. United States of America	6865.24	54.55	34.43	31.70
5486.91	20. Monaco	20. San Marino	6854.65	24.47	27.47	24.42

Legend: High SDI; High-middle SDI; Middle SDI; Low-middle SDI

Figure S2. Global distribution of relative changes in anxiety disorders prevalence from 1992 to 2021 and the top 20 countries by age-standardized prevalence in 1992 and 2021.

Notes: (A) Global map for the relative change of prevalence from 1992 to 2021. (B) Ranking graphs for age-standardized prevalence. Dashed lines indicate decreasing rankings, and solid lines indicate increasing rankings. YLDs, years lived with disability; SDI, socio-demographic index.

Source: Institute for Health Metrics and Evaluation. Used with permission. *All rights reserved.*

(A)

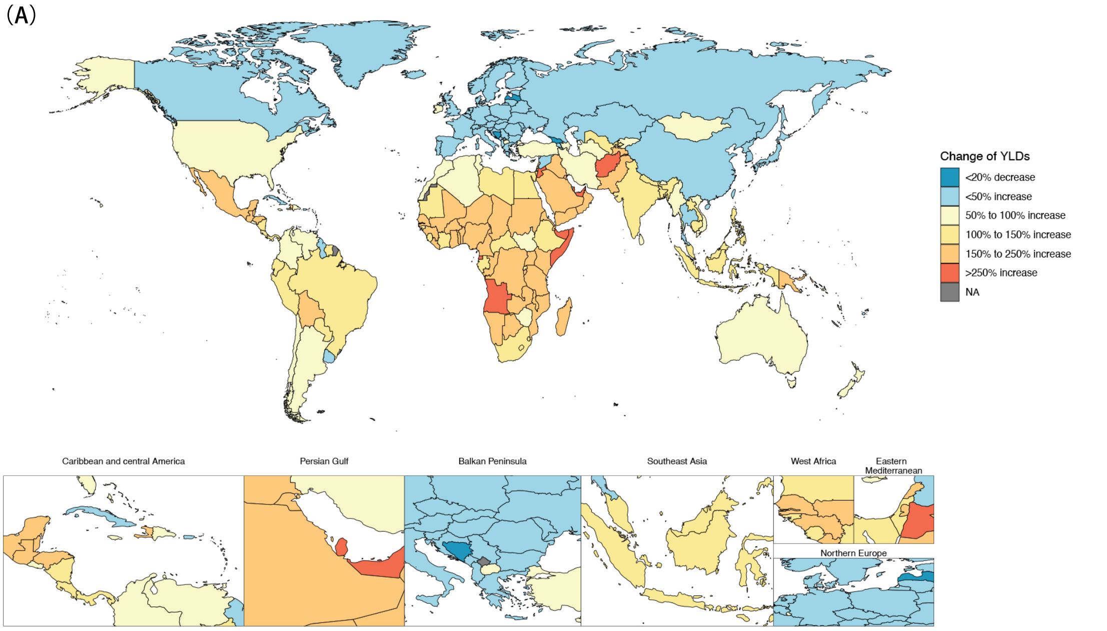

(B)

Age-standardized YLD rate in 1992 (per 100,000)	Leading countries in 1992
905.47	1. Portugal
862.42	2. New Zealand
854.62	3. Norway
802.10	4. Switzerland
800.05	5. Iran (Islamic Republic of)
789.53	6. Paraguay
783.95	7. Netherlands
766.33	8. Ireland
747.25	9. France
720.54	10. Lebanon
717.42	11. Germany
709.88	12. Cyprus
706.39	13. Malta
705.58	14. Brazil
684.77	15. Austria
667.65	16. Greece
667.04	17. Italy
659.00	18. Australia
658.89	19. San Marino
656.68	20. Monaco

Leading countries in 2021	Age-standardized YLD rate in 2021 (per 100,000)	Percentage change in age-standardized YLD rate, 1992-2021	Percentage change in age-standardized incidence rate, 1992-2021	Percentage change in age-standardized prevalence rate, 1992-2021
1. Portugal	1156.75	27.75	31.87	27.37
2. Brazil	1058.67	50.04	41.15	49.86
3. Paraguay	992.12	25.66	29.30	25.90
4. Lebanon	981.03	36.15	42.77	35.92
5. Iran (Islamic Republic of)	973.70	21.70	23.90	21.53
6. Netherlands	942.86	20.27	27.50	20.44
7. Ireland	939.19	22.56	25.77	22.57
8. France	916.74	22.68	26.41	22.51
9. Switzerland	905.93	12.94	15.01	12.66
10. Germany	904.80	26.12	31.22	25.99
11. Norway	901.75	5.51	10.11	5.18
12. Bolivia (Plurinational State of)	899.89	42.66	48.20	42.15
13. New Zealand	880.94	2.15	2.81	1.72
14. Peru	866.21	39.04	43.55	71.23
15. Greece	860.23	28.84	32.01	28.88
16. Austria	846.35	23.60	26.50	22.95
17. Cyprus	836.76	17.87	19.37	17.60
18. San Marino	819.76	24.42	27.47	24.47
19. Tunisia	816.84	36.85	41.36	37.42
20. Italy	813.97	22.03	26.20	21.52

High SDI
High-middle SDI
Middle SDI
Low-middle SDI

Figure S3. Global distribution of relative changes in anxiety disorders years lived with disability from 1992 to 2021 and the top 20 countries by age-standardized years lived with disability in 1992 and 2021.

Notes: (A) Global map for the relative change of YLDs from 1992 to 2021. (B) Ranking graphs for age-standardized YLDs. Dashed lines indicate decreasing rankings, and solid lines indicate increasing rankings. YLDs, years lived with disability; SDI, socio-demographic index.
Source: Institute for Health Metrics and Evaluation. Used with permission. *All rights reserved.*

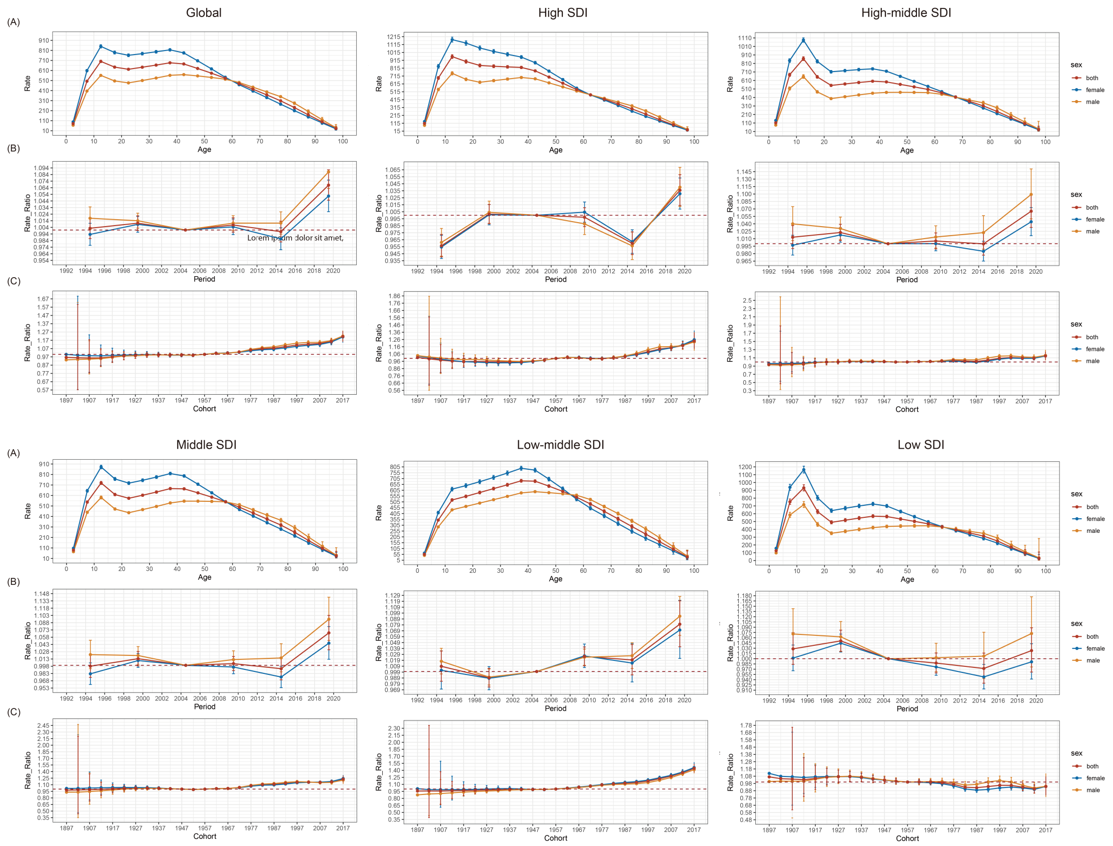

Figure S4. Parameter estimates of age, period, and cohort effects on the incidence of anxiety disorders stratified by sex, globally and across five SDI regions from 1992 and 2021.

Note: (A) Age effects are shown by the fitted longitudinal age curves of incidence rates (per 100,000 person-years) adjusted for period deviations. (B) Period effects are shown by the relative risk of incidence and computed as the ratio of age-specific rates from 1992–1996 to 2017–2021, with the reference period set at 2002–2006. (C) Cohort effects are shown by the relative risk of incidence and computed as the ratio of age specific rates from the 1897 birth cohort to the 2017 cohort, with the reference cohort set at 1957. The data points and error bars denote incidence rates or rate ratios and their corresponding 95% CIs. SDI, socio-demographic index. CI, confidence interval.

Source: Institute for Health Metrics and Evaluation. Used with permission. *All rights reserved.*

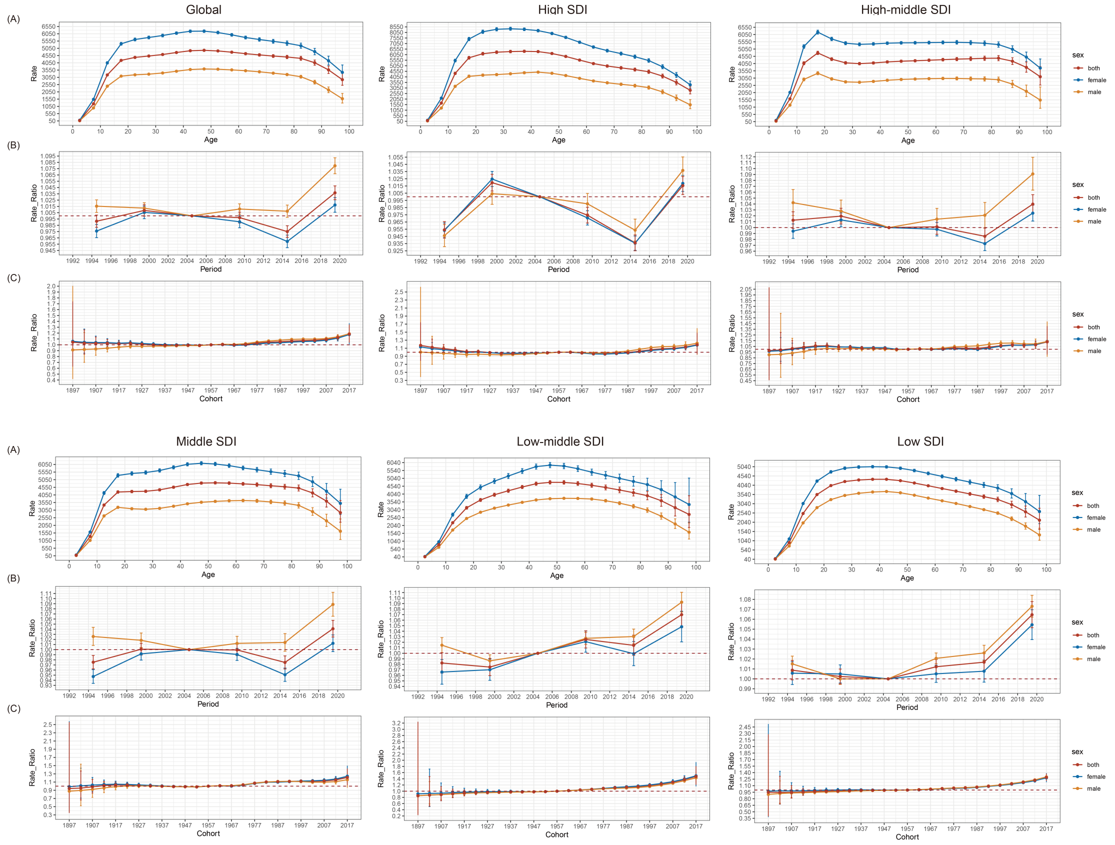

Figure S5. Parameter estimates of age, period, and cohort effects on the prevalence of anxiety disorders stratified by sex, globally and across five SDI regions from 1992 and 2021.

Note: (A) Age effects are shown by the fitted longitudinal age curves of prevalence rates (per 100,000 person-years) adjusted for period deviations. (B) Period effects are shown by the relative risk of prevalence and computed as the ratio of age-specific rates from 1992–1996 to 2017–2021, with the reference period set at 2002–2006. (C) Cohort effects are shown by the relative risk of prevalence and computed as the ratio of age specific rates from the 1897 birth cohort to the 2017 cohort, with the reference cohort set at 1957. The data points and error bars denote prevalence rates or rate ratios and their corresponding 95% CIs. SDI, socio-demographic index. CI, confidence interval.

Source: Institute for Health Metrics and Evaluation. Used with permission. All rights reserved.

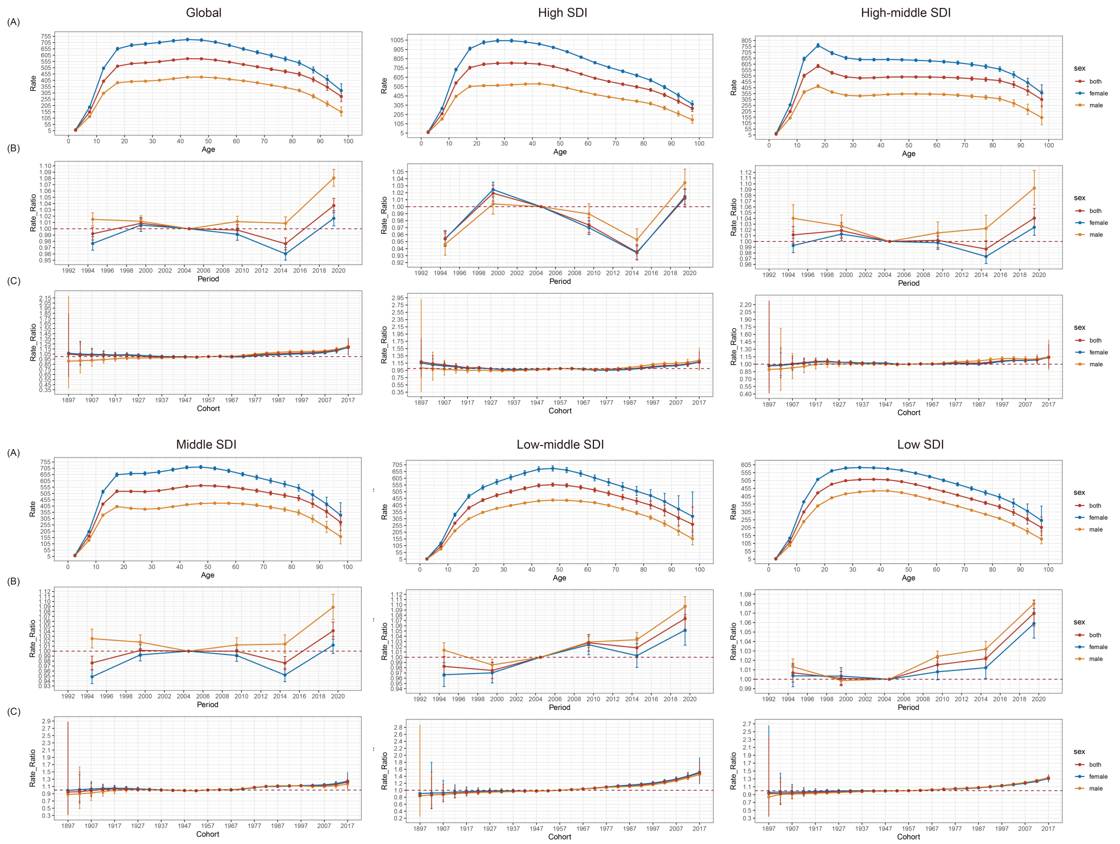

Figure S6. Parameter estimates of age, period, and cohort effects on the years lived with disability of anxiety disorders stratified by sex, globally and across five SDI regions from 1992 and 2021.

Note: (A) Age effects are shown by the fitted longitudinal age curves of YLDs rates (per 100,000 person-years) adjusted for period deviations. (B) Period effects are shown by the relative risk of YLDs and computed as the ratio of age-specific rates from 1992–1996 to 2017–2021, with the reference period set at 2002–2006. (C) Cohort effects are shown by the relative risk of YLDs and computed as the ratio of age specific rates from the 1897 birth cohort to the 2017 cohort, with the reference cohort set at 1957. The data points and error bars denote YLDs rates or rate ratios and their corresponding 95% CIs. SDI, socio-demographic index. YLDs, years lived with disability. CI, confidence interval.

Source: Institute for Health Metrics and Evaluation. Used with permission. *All rights reserved.*

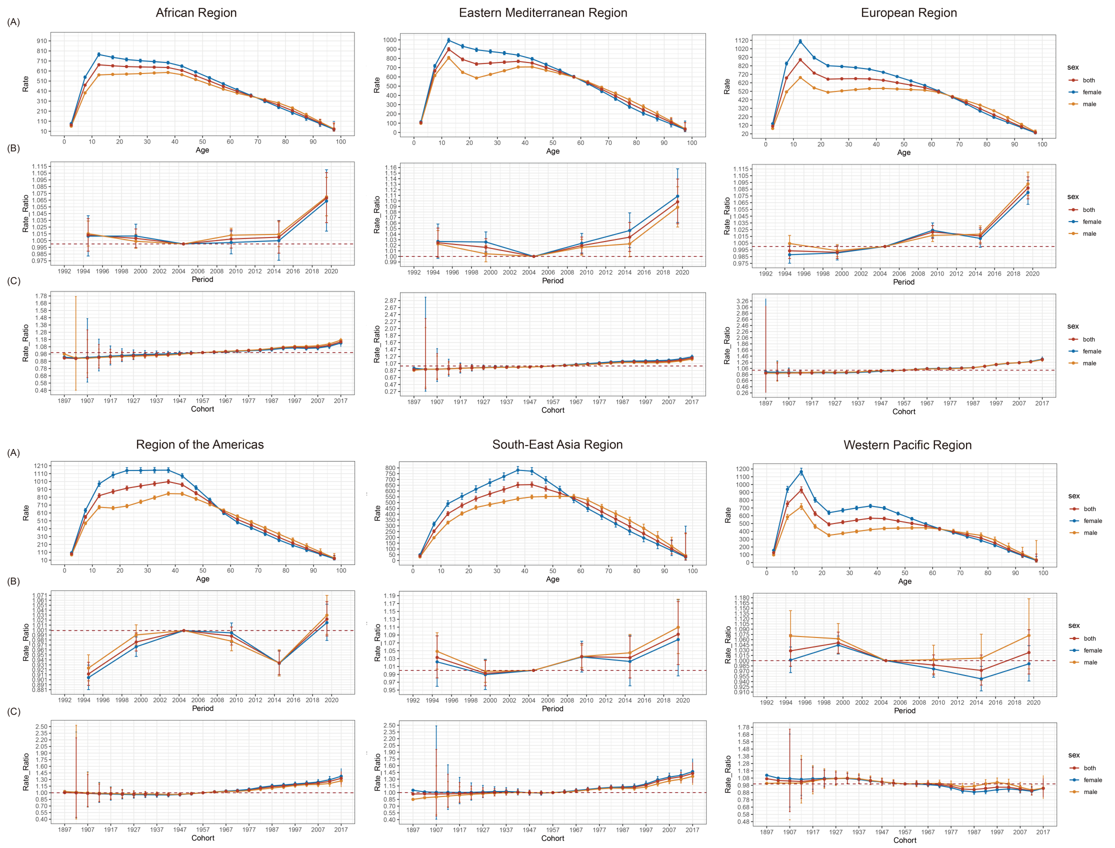

Figure S7. Parameter estimates of age, period, and cohort effects on the incidence of anxiety disorders, stratified by sex, across the six WHO regions from 1992 and 2021.

Note: (A) Age effects are shown by the fitted longitudinal age curves of incidence rates (per 100,000 person-years) adjusted for period deviations. (B) Period effects are shown by the relative risk of incidence and computed as the ratio of age-specific rates from 1992–1996 to 2017–2021, with the reference period set at 2002–2006. (C) Cohort effects are shown by the relative risk of incidence and computed as the ratio of age specific rates from the 1897 birth cohort to the 2017 cohort, with the reference cohort set at 1957. The data points and error bars denote incidence rates or rate ratios and their corresponding 95% CIs. WHO, world health organization. CI, confidence interval.
Source: Institute for Health Metrics and Evaluation. Used with permission. *All rights reserved.*

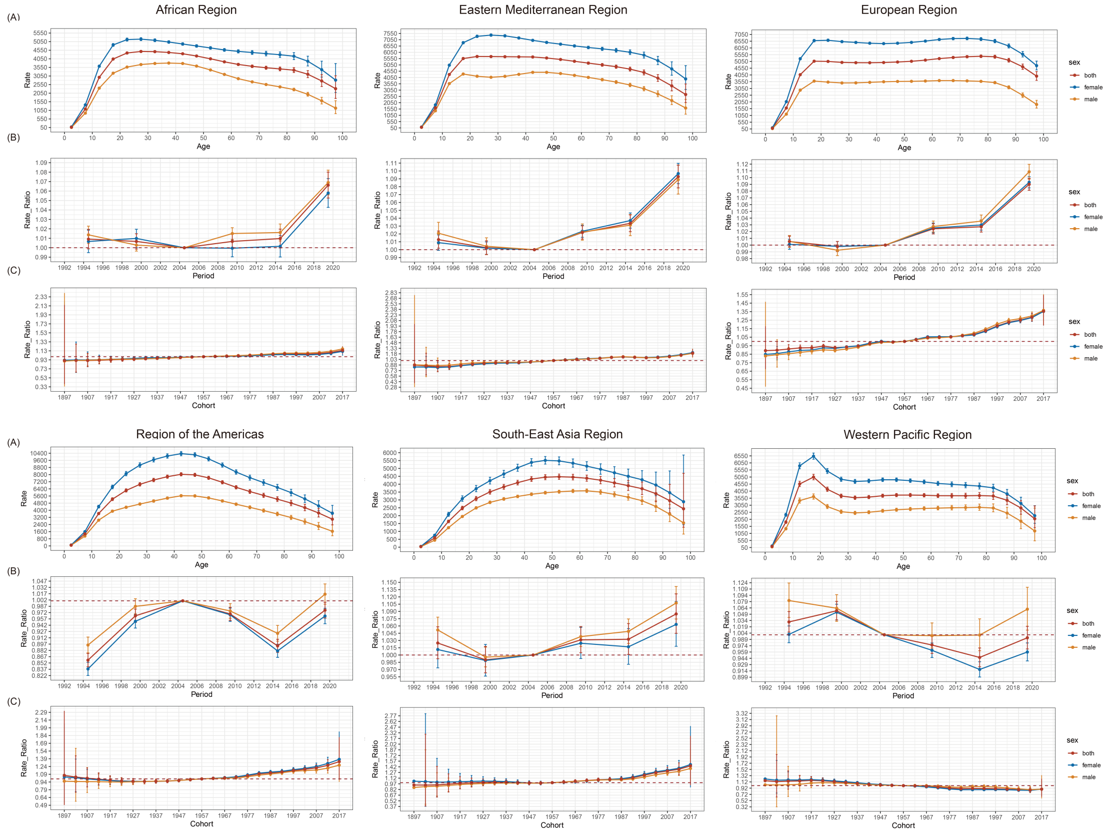

Figure S8. Parameter estimates of age, period, and cohort effects on the prevalence of anxiety disorders stratified by sex, across the six WHO regions from 1992 and 2021.

Note: (A) Age effects are shown by the fitted longitudinal age curves of prevalence rates (per 100,000 person-years) adjusted for period deviations. (B) Period effects are shown by the relative risk of prevalence and computed as the ratio of age-specific rates from 1992–1996 to 2017–2021, with the reference period set at 2002–2006. (C) Cohort effects are shown by the relative risk of prevalence and computed as the ratio of age specific rates from the 1897 birth cohort to the 2017 cohort, with the reference cohort set at 1957. The data points and error bars denote prevalence rates or rate ratios and their corresponding 95% CIs. WHO, world health organization. CI, confidence interval.

Source: Institute for Health Metrics and Evaluation. Used with permission. All rights reserved.

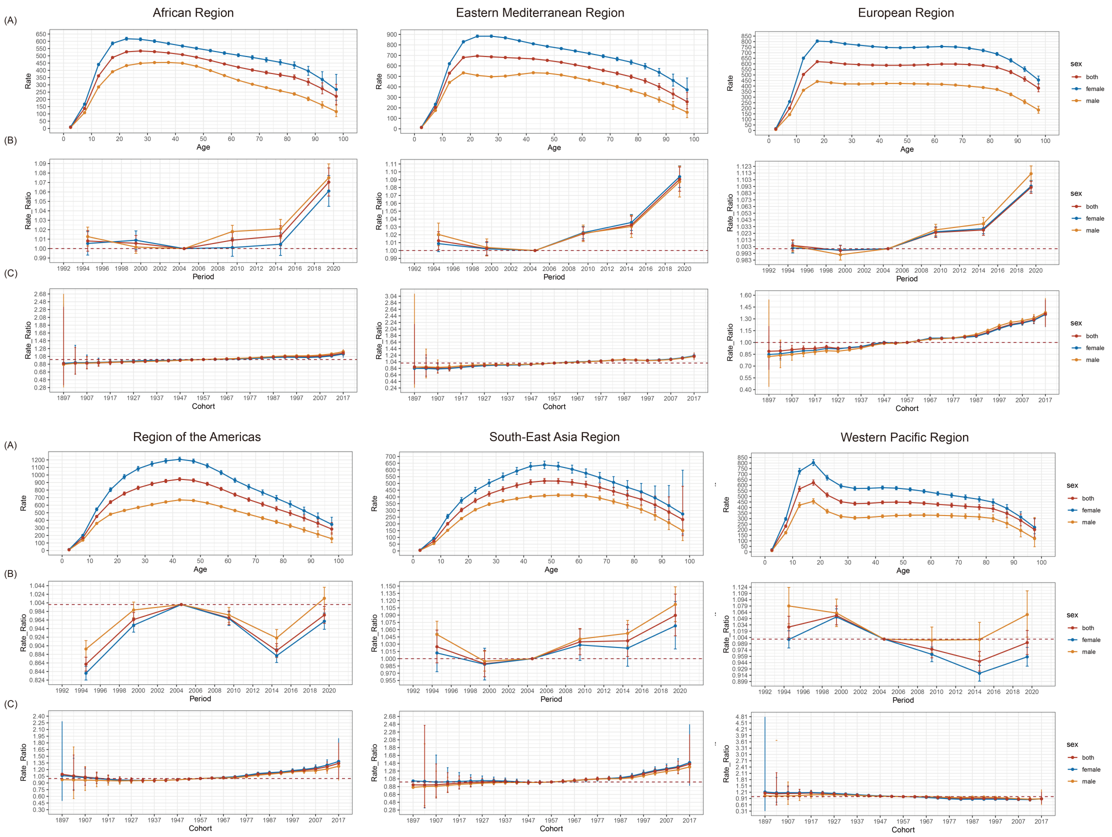

Figure S9. Parameter estimates of age, period, and cohort effects on the years lived with disability of anxiety disorders stratified by sex, across the six WHO regions from 1992 and 2021.

Notes: (A) Age effects are shown by the fitted longitudinal age curves of YLDs rates (per 100,000 person-years) adjusted for period deviations. (B) Period effects are shown by the relative risk of YLDs and computed as the ratio of age-specific rates from 1992–1996 to 2017–2021, with the reference period set at 2002–2006. (C) Cohort effects are shown by the relative risk of YLDs and computed as the ratio of age specific rates from the 1897 birth cohort to the 2017 cohort, with the reference cohort set at 1957. The data points and error bars denote YLDs rates or rate ratios and their corresponding 95% CIs. WHO, world health organization. YLDs, years lived with disability. CI, confidence interval.
Source: Institute for Health Metrics and Evaluation. Used with permission. All rights reserved.
